# Activation of Primary and Secondary Benzylic and Tertiary Alkyl (sp^*3*^)C-F Bonds Inside a Self-Assembled Molecular Container

**DOI:** 10.3389/fchem.2018.00639

**Published:** 2019-01-04

**Authors:** Jesper M. Köster, Daniel Häussinger, Konrad Tiefenbacher

**Affiliations:** ^1^Department of Chemistry, University of Basel, Basel, Switzerland; ^2^Department of Biosystems Science and Engineering, ETH Zürich, Basel, Switzerland

**Keywords:** supramolecular catalysis, molecular capsules, supramolecular chemistry, acid catalysis, elimination

## Abstract

Alkyl fluorides are generally regarded as chemically inert. However, several literature examples describe the activation of alkyl (sp3)C-F bonds via strong Brønsted or Lewis acids under harsh conditions. We here report that catalytic amounts of the self-assembled resorcinarene capsule are able to activate alkyl (sp3)C-F bonds under mild conditions (40°C, no strong Brønsted or Lewis acid present). Kinetic measurements display a sigmoidal reaction progress after an initial induction period. Control experiments indicate that the presence of the supramolecular capsule is required for an efficient reaction acceleration.

## Introduction

**Figure 1:**
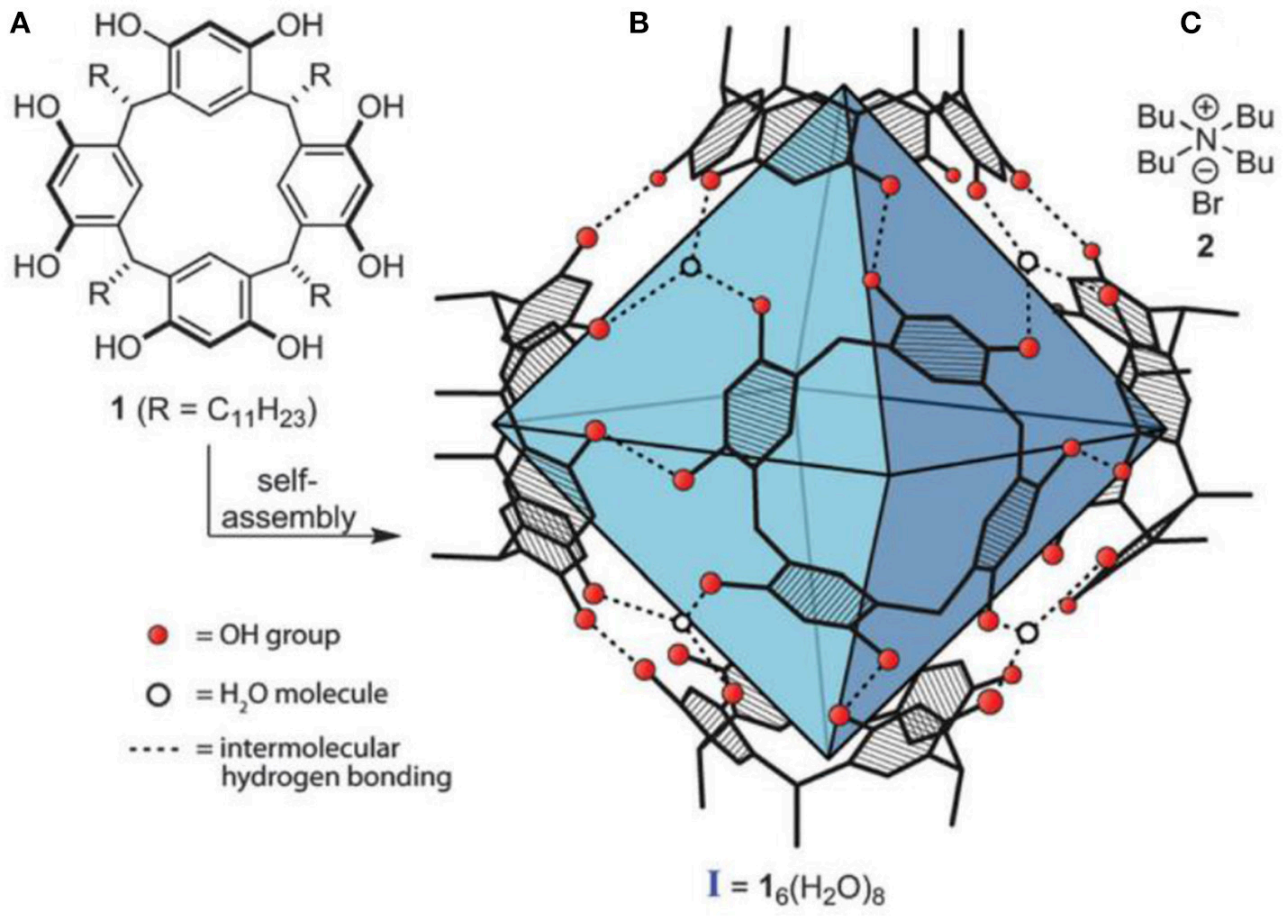
**(A)** C_11_-resorcinarene (**1**); **(B)** hexameric capsule **I**, C_11_-feet omitted; **(C)** competitive guest TBAB (**2**).

Catalysis inside closed binding pockets, much like inside enzymes, has been a challenging research area but in recent decades active supramolecular systems have been reported (Marchetti and Levine, 2011; Wiester et al., 2011; Raynal et al., 2014; Brown et al., 2015; Leenders et al., 2015; Zarra et al., 2015; Catti et al., 2016a,b; Cullen et al., 2016; Jans et al., 2016; Kuijpers et al., 2016; Levin et al., 2016; Otte, 2016; Wang et al., 2016, 2017; Bräuer et al., 2017; Ueda et al., 2017; Catti and Tiefenbacher, 2018; La Manna et al., 2018a). Drawing upon a multitude of structural classes such as coordination cages, metal-organic frameworks, and non-covalent assemblies, a whole series of reactions were successfully catalyzed. One of the catalytically active supramolecular containers is the hexameric resorcinarene capsule **I** (see Figure 1B), which self-assembles in non-polar solvents (e.g., chloroform or benzene) from six molecules of resorcinarene (**1**, Figure 1A) and eight water molecules (MacGillivray and Atwood, 1997; Avram and Cohen, 2002). It possesses an internal volume of ~1,400 A3 and is readily accessible in a one-step condensation from resorcinol and dodecanal. It was demonstrated to serve as a mild Brønsted acid (pK_A_ ~ 5.5–6) and functions as a non-nucleophilic counterion due to the extensive delocalization of the negative charge (Zhang and Tiefenbacher, 2013).

The supramolecular capsule **I** was used as a catalyst in the hydrolysis of acetals (Zhang and Tiefenbacher, 2013), the tail-to-head cyclization of terpenes (Zhang and Tiefenbacher, 2015), the hydroalkoxylation of alkenes (Catti and Tiefenbacher, 2015), the cyclodehydration of alcohols (Catti et al., 2017), the carbonyl-olefin metathesis (Catti and Tiefenbacher, 2018), the hydration of isonitriles (Bianchini et al., 2013), [2+3]-cycloaddition reactions (Giust et al., 2015; La Manna et al., 2018a), the Meinwald rearrangement of epoxides (Caneva et al., 2016), the hydration of alkynes (La Sorella et al., 2016a), the oxidation of thioethers (La Sorella et al., 2016b), and Friedel-Crafts reactions (La Manna et al., 2018b).

Recently, we investigated “inert” fluorinated acyclic terpenes as probes to quantify substrate encapsulation via ^19^F-NMR. Surprisingly, the C-F bond of tertiary fluoride **10** (Table 1) was activated in the presence of catalytic amounts of capsule (10 mol%). This observation prompted us to investigate this reaction more closely and here we present the results.

**Table 1:** Substrate overview with yields; product denominations: **a**, alcohol; **e**, cyclic ether; **i**, internal alkene; **t**, terminal alkene; all reactions run in filtered CDCl_3_.

**Entry**	**Substrate**	**Products**		**Background**
1	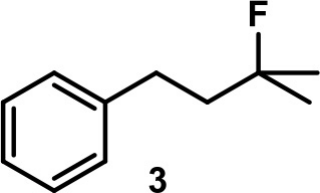	**3i** 69%^a^ (16 h) **3t** 13%^a^		0^b^ 0^c^
2	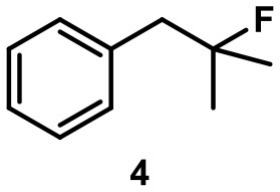	**4i** 45%^a^ (16 h) **4a** 35%^a^ **4t** 16%^a^		0^b^ 0^c^
3	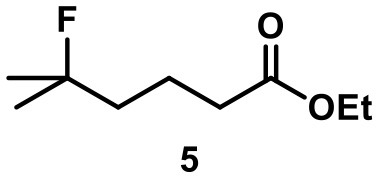	**5I** 60%^a^ (20 h) **5i** 29%^a^ **5t** 3%^a^	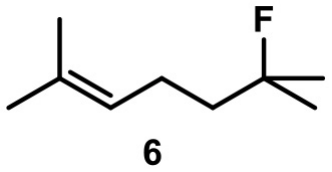	0^b^ 0^c^
4	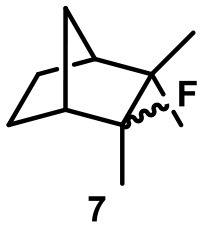	**6e** 81%^a^ (21 h) **6i** 11%^a^	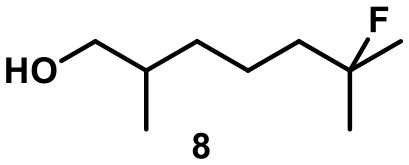	0^b^ 0^c^
5	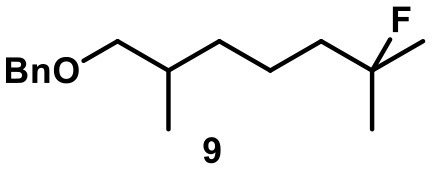	**7t** 98%^a^ (2 h)		**7t** 98%^b^ (20 h) 0^c^
6	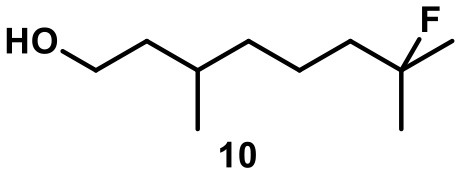	**8e** 72%^a^ (4 h)	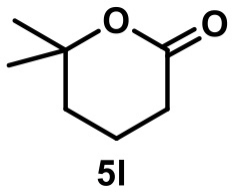	**8e** 71%^b^ (7d) 0^c^
7	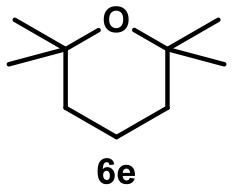	**9i** 65%^a^(4 h) **9t** 6%^a^		**9i** 5%^b^(7d) 0^c^
8	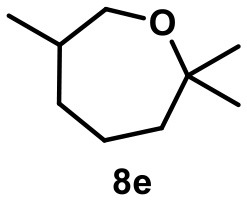	**10i** 60%^a^ (4 h) **10a** 6%^a^ **10t** 16%^a^		**10i** 52%^b^ (7d) **10a** 6%^b^ **10t** 13%^a^ 0^c^

*All reactions run in CDCl_3_ (filtered over basic Al_2_O_3_ prior to use) at 40°C; ^a^determined via ^1^H NMR ^b^addition of TBAB (1.5 eq. relative to capsule **I**), reaction run for 7 d at 40°C ^c^ addition of HOAc (10 mol%), no hexamer present, 7d, 40°C*.

Alkyl halides, known for their broad spectrum of reactivity (substitution, elimination, metalation), are a cornerstone of organic chemistry. In contrast to iodides, bromides, and chlorides alkyl fluorides display a general lack of reactivity under typical conditions for halide chemistry. Contrary to intuition, the exceptionally high electronegativity of fluorine does not facilitate nucleophilic attacks on the adjacent carbon but instead strengthens the (sp^3^)C-F bond (105.4 kcal/mol, C-H: 98.8 kcal/mol, C-Cl: 78.5 kcal/mol), due to substantial electrostatic attraction between the highly polarized bonding partners (O'Hagan, 2008). Strategies that work for alkyl chlorides, bromides, or iodides seem to fail to sufficiently activate fluorides for further reactions. Nevertheless, some successful approaches for C-F bond activation have been developed (Burdeniuc et al., 1997; Amii and Uneyama, 2009; Kuehnel et al., 2013; Shen et al., 2015). Exploiting fluorine's high affinity toward boron, and aluminum, most examples cover activation by Lewis acids. Activation by Brønsted acids, known since 1949, when Le Fave found that heating trifluorotoluene in concentrated sulfuric acid facilitated the hydrolysis to benzoic acid (Fave, 1949), has received relatively little attention by contrast. Wang et al. recently published an improved take on this approach to activate trifluorotoluyl substrates by employing an excess of triflic acid at ambient temperature (Wang and Hu, 2009). The benzyl acylium ions generated by this method were then converted to benzophenone derivatives by adding arene nucleophiles to promote a Friedel-Crafts acylation. The inert nature of the CF_3_ group requires the use of superstoichiometric amounts of strong acids, which limits the functional group tolerance considerably.

Although the topic of organic fluorides as hydrogen bond acceptors is controversial, increasing evidence indicates a weak interaction (Champagne et al., 2015a). A mild method of activation for benzylic fluorides via hydrogen bonding was recently developed by Paquin et al. (Champagne et al., 2014, 2015b). Hexafluoroisopropanol (HFIP) serves as hydrogen bond donor to the fluorine atom, which destabilizes the C-F bond to then generate a benzylic cation, which can be intercepted by an arene nucleophile in a Friedel-Crafts reaction. HF is liberated after the conversion and replaces HFIP as the catalytically active species because of its higher hydrogen bond donating ability (Rosenberg, 2012; Champagne et al., 2014).

**Figure 2:**
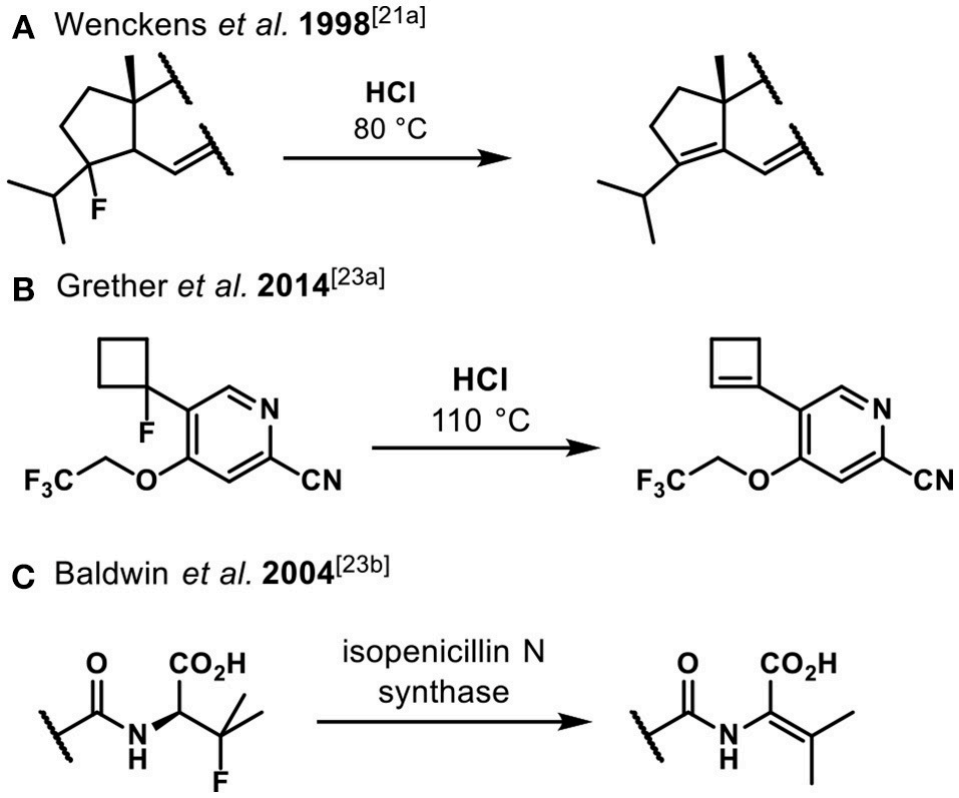
Examples of Brønsted acid- and enzyme-promoted elimination of tertiary fluorides **(A)** Wenckens et al. (1998), **(B)** Grether et al. (2013) and **(C)** Grummitt et al. (2004).

Activation of non-benzylic fluorides remains challenging under Brønsted acidic conditions. Under harsh Brønsted acidic conditions (HCl, 80–110°C) elimination of tertiary alkyl fluorides was reported (Figures 2A,B). Primary and secondary fluorides show no reactivity due to the poor stabilization of the respective intermediate carbocations (Wenckens et al., 1998; Bellezza et al., 2005). Interestingly, the enzyme pocket of isopenicillin N synthase facilitated the elimination of a tertiary fluoride under mild conditions (Figure 2C).

## Results and Discussion

Similarly to the enzyme pocket (see above), catalytic amounts of capsule **I** (10 mol%) also facilitated the activation of tertiary alkyl fluorides. In light of our recent investigations (Köster and Tiefenbacher, 2018) concerning the role of HCl as a cocatalyst for some reactions catalyzed by capsule **I**, it is important to note that the activation of tertiary fluorides reported here does not require an acid cocatalyst.

The aryl-substituted substrates **3** and **4** (Table 1, entries 1 and 2) were mainly transformed to the corresponding homostyrene **3i** and styrene **4i**, respectively. In both cases the formation of the trisubstituted alkene was favored and the terminal alkenes **3t** and **4t** were observed to a smaller degree. Brønsted-acid catalyzed isomerization of homostyrene **3i** to the corresponding styrene was not observed. Furthermore, an intramolecular Friedel-Crafts alkylation could not be observed. Considerable amounts of homobenzylic alcohol **4a** were formed as a side product. It is important to note that no background reaction was observed when the capsule was blocked with inhibitor **2** (see Figure 1C, 1.5 eq. tetrabutylammonium bromide per capsule) or when the capsule was omitted and replaced with an acid of similar acidity (acetic acid). These control experiments indicate that the reaction takes place inside the binding pocket of capsule **I**.

The main product of the reaction of ester **5** was found to be lactone **5l**, presumably resulting from a nucleophilic attack of the ester carbonyl oxygen on the tertiary carbocation. The elimination pathway also led to the formation of the tri-substituted alkene **5i** and of terminal alkene **5t** in trace amounts. Again, no background reactions were observed.

When the unsaturated fluoride **6** was investigated, the main product was found to be cyclic ether **6e** (see Table 1). Nucleophilic interception of the initially formed tertiary carbocation by water (from the hexameric assembly or the bulk solution) presumably leads to the formation of an alkenol. The ensuing cyclization is known to be catalyzed by capsule **I** (Catti and Tiefenbacher, 2015). As a side reaction, elimination to the trisubstituted alkene was observed.

Camphene-derived fluoride **7** was converted to the alkene **7t** within 2 h quantitatively. The addition of inihibitor **2** did not result in suppression of the reaction but only slowed down the reaction (completion after 20 h). The complete selectivity toward elimination may be explained by the steric constrains of the carbocation, with a quaternary center and a bridgehead atom in close proximity, which effectively shield the cation from the attack of any nucleophiles.

The fluoroalcohol **8** was converted to the seven-membered cyclic ether with good selectivity. The addition of inhibitor **2** did not prevent the reaction but slowed it down significantly (7 d instead of 4 h). The benzyl-protected analog **9** was transformed mainly to the corresponding trisubstituted alkene **9i** because the cyclization pathway was blocked. Experiments with a blocked cavity revealed only marginal conversion after 7 d. The β-Citronellol-derived fluoride **10** showed complete conversion within 4 h with the major product being the trisubstituted alkene. The formation of the corresponding eight-membered cyclic ether could not be observed. As observed with **8**, substantial conversion could be observed in presence of **2**, although at reduced rates (7 d instead of 4 h).

A possible explanation for the conversion of substrates in the presence of inhibitor may lie in the ability of capsule **I** to bind substrates via H-bonds from the outside. However, the significantly reduced reaction rates point toward an acceleration of the reaction on the inside of **I**. All substrates remained intact upon prolonged exposure to 10 mol% acetic acid (pK_A_ = 4.75), indicating that the hexamer's (pK_A_ ~ 5.5–6) mode of activation is likely based on hydrogen bonding to the substrate and stabilization of the resulting cation via cation-π interactions instead of Brønsted acidity alone.

**Scheme 1:**
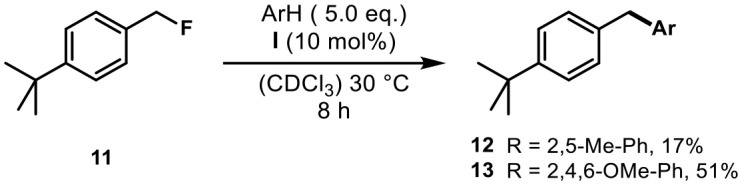
Friedel-Crafts benzylation with benzyl fluoride **11** inside the cavity.

After having demonstrated the ability of capsule **I** to activate tertiary alkyl fluorides, we wondered whether the Friedel-Crafts alkylation with benzylic fluoride electrophiles was also possible inside the hexameric capsule **I**. Starting with 4-tert-butyl benzyl fluoride (**11**) and *p*-xylene (Scheme 1), a reaction to the expected product could be observed, albeit in low yield (17%). Since it is known that unfunctionalized guests are taken up poorly by container **I**, we employed 1,3,5-trimethoxybenzene as arene nucleophile to take advantage of the efficient uptake of oxygenated species by capsule **I**. This indeed resulted in an increase in yield to 51%. These results clearly indicate that the activation of benzylic fluorides is also possible and further corroborate capsule **I**‘s ability to accelerate Friedel-Crafts reactions as was recently demonstrated by La Manna et al. (2018b). In the absence of suitable nucleophiles, oligomerization was observed with primary and secondary benzyl fluorides. When benzylic di- and trifluorides were investigated, no reaction could be observed. Attempts to use tertiary alkyl fluorides as electrophiles for Friedel-Crafts reactions were unsuccessful, leading only to the respective elimination products.

**Scheme 2:**
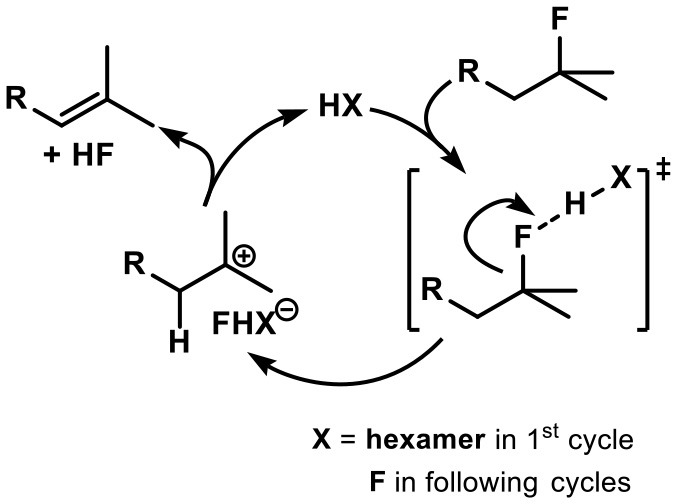
Proposed mechanism of the HF elimination inside container **I**.

Following the findings of Paquin et al. (Champagne et al., 2014, 2015a,b) we propose an autocatalytic cycle starting with the encapsulation of a substrate molecule (see Scheme 2). The fluorine substituent likely is interacting *via* a hydrogen bond to a water molecule of the assembly. Subsequent cleavage of the activated C-F bond then leads to formation of a carbocation and hydrogen bonded fluoride^1^. After elimination of a proton and subsequent recombination with fluoride to form HF, the alkene is released from the inner cavity. With its superior hydrogen bond donating ability (Checinska and Grabowski, 2006; Champagne et al., 2014) hydrogen fluoride acts as catalytically active species in ensuing cycles.

**Figure 3:**
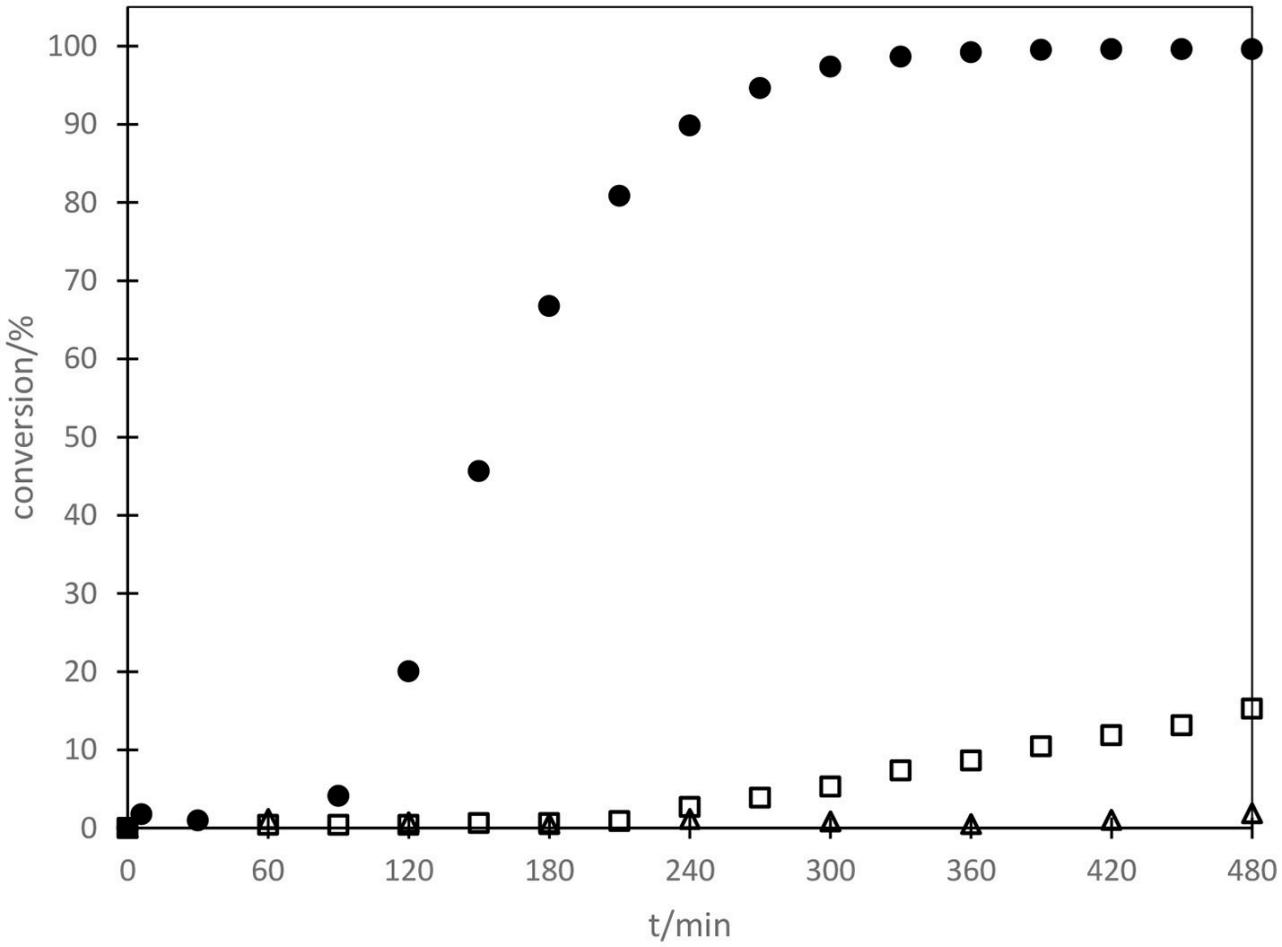
Reaction progress for the elimination of HF from **3** in presence of capsule **I**; 30 °C (□); 40 °C (•), 10 mol% HF, no capsule present, 40°C (Δ).

This mechanistic proposal was underlined by kinetic measurements of the reaction of **3** inside container **I**. After an induction period a sigmoidal reaction progress can be observed at 40°C, a typical indication of an autocatalytic reaction (see Figure 3) (Anslyn and Dougherty, 2006).

Interestingly, the addition of HF did not initiate a rapid autocatalytic conversion in the absence of capsule **I**. Conversion of the starting fluoride after addition of catalytic amounts of hydrogen fluoride was sluggish, reaching only 2% after 8 h. A probable cause of this observation might be the fast equilibration of hydrogen fluoride with silicon of the glass reaction vessel walls, which greatly diminishes the local concentration of the catalytically active species. Although HF has been shown to exist in equilibrium with glass walls (Chapman and Levy, 1952), only minuscule amounts of HF may exist at any given time in solution. Interestingly, experiments in plastic vials (see SI chapter 5.2) indicated that this is not true. Addition of HF in the absence of hexamer **I** did not lead to a significant conversion even when the reaction was run in plastic vials. However, in the presence of hexamer **I** the addition of HF initiated the reaction immediately (no induction period observed). This strongly suggests a synergistic catalysis mediated by HF and capsule **I**. Control reactions with a large substrate (see SI chapter 5.5) that is unable to enter the cavity of **I**, showed no conversion within 4 days under standard reaction conditions (40°C, acid-free CDCl_3_, 10 mol% **I**). However, a competition experiment together with benzyl ether **9** resulted in the full conversion of both substrates within 4 h (see SI-Figure 6). It is clearly visible that the smaller substrate **9** initiated the reaction and subsequently the larger substrate reacted, obviously outside of capsule **I**. In summary, this suggests that: (1) capsule **I** is necessary to activate the substrate, (2) HF is only catalytically active in conjunction with capsule **I** (3) if HF has been liberated large substrates can also react outside of capsule **I**, in accordance with the proposed mechanism (see Scheme 2).

Kinetic experiments with monodeuterated substrate **3d** (see SI chapter 5.3) to elucidate the reaction mechanism resulted in a KIE of 1.16 ± 0.03. This is indicative of a secondary deuterium KIE arising from hyperconjugative stabilization of the formed cation, thereby indicating fluoride abstraction as rate-limiting (Anslyn and Dougherty, 2006). This result is in agreement with earlier observations made in the acid-catalyzed solvolysis of tertiary alkyl fluorides (Chapman and Levy, 1952).

In conclusion, we have demonstrated the ability of **I** to activate tertiary alkyl fluorides and primary and secondary benzylic fluorides. The hexameric assembly **I** presumably not only functions as a hydrogen bond donor to activate the C-F bond but is also able to stabilize the intermediate carbocations *via* cation-π interactions. The stabilized intermediates underwent several transformations inside the cavity, ranging from elimination, cyclization to intermolecular nucleophilic attack in the case of benzyl fluorides. The displayed ability of hexamer **I** to activate aliphatic C-F bonds is noteworthy since the reaction conditions are unusually mild: 40°; no strong Brønsted or Lewis acids present.

## Author Contributions

KT conceived and supervised the project. KT and JK planned the project. JK carried out all the experiments. DH performed kinetic NMR measurements. JK and KT compiled the first draft of the manuscript. All authors contributed to the final version of the manuscript.

### Conflict of Interest Statement

The authors declare that the research was conducted in the absence of any commercial or financial relationships that could be construed as a potential conflict of interest.

## Footnotes

^1^If X = F, the ion is known as bifluoride, however, more equivalents of HF could be involved.
