# Supplementary material for: Activation of Primary and Secondary Benzylic and Tertiary Alkyl (sp3)C-F Bonds Inside a Self-Assembled Molecular Container
Source: Front Chem. 2019 Jan 4;6:639. doi: 10.3389/fchem.2018.00639 (PMC6328483; doi:10.3389/fchem.2018.00639)
Supplement: Supplementary file 1 [file Data_Sheet_1.PDF]

Electronic Supplementary Information

Activation of tertiary alkyl and primary benzylic (sp^3)C-F bonds inside a self-assembled molecular container

Jesper M. Köster[a], Daniel Häussinger[a], and Konrad Tiefenbacher[a,b]*

[a]*Department of Chemistry, BPR 1096, P.O. box 3350, Mattenstrasse 24a, University of Basel, CH-4058 Basel, Switzerland*

and

[b]*Department of Biosystems Science and Engineering, Mattenstrasse 24, ETH Zürich, CH-4058 Basel, Switzerland*

**Correspondence to:* konrad.tiefenbacher@unibas.ch / tkonrad@ethz.ch

Table of contents

1. General information 1
2. Synthesis of *C*-Undecylcalix[4]resorcinarene (**1**) 2
3. Substrate syntheses 3
   - 3.1 Unreactive substrates 12
   - 3.2 Unselective substrates 15
   - 3.3 Synthesis of deuterated substrate 3d 16
   - 3.4 Synthesis of products unknown to literature 17
   - 3.5 Synthesis of large substrate **28** 18
4. Elimination of HF: determination of yields 24
5. Mechanistic investigations 27
   - 5.1 Reaction kinetics 27
   - 5.2 Experiments in plastic vials 28
   - 5.3 KIE experiments 29
   - 5.4 Substrate uptake 30
   - 5.5 Reaction of large substrate **28** 32
   - 5.6 Influence of reaction vessel 33
6. Control experiments 34
   - 6.1 Blocked cavity 34
   - 6.2 Blocked cavity after induction period 35
   - 6.3 Without catalyst 36
   - 6.4 With catalytic amounts of acetic acid in solution 36
7. References 37
8. NMR spectra 38

1. General information

Experimental: Reactions were carried out under an atmosphere of argon unless otherwise indicated. Analytical thin-layer chromatography (TLC) was performed on *Merck* silica gel 60 F_{254} glass-baked plates, which were analyzed after exposure to standard staining solutions (basic $KMnO_4$ or cerium ammonium molybdate). ^{1}H NMR spectra were recorded at 250 MHz, 400 MHz or 500 MHz, using a *Bruker* AV 250, AV 400 and AV 500 spectrometer respectively. ^{13}C NMR spectra were recorded at 101 MHz on a *Bruker* 400 MHz spectrometer. ^{19}F NMR spectra were recorded at 235 MHz on a *Bruker* 250 MHz and at 376 MHz on a *Bruker* 400 MHz spectrometer. Chemical shifts of ^{1}H NMR, ^{13}C NMR and ^{19}F NMR (measured at 298 K) are given in ppm by using $CHCl_3$, $CDCl_3$, and $CFCl_3$ as references (7.26 ppm, 77.16 ppm, and 0.00 ppm respectively). Coupling constants (*J*) are reported in Hertz (Hz). Standard abbreviations indicating multiplicity were used as follows: s(singlet), d (doublet), dd (doublet of doublets), t (triplet), m (multiplet). Infrared spectra were recorded on a *Varian* 800 FT-IR spectrometer. High-resolution mass spectra were obtained using the electron impact ionization (EI) technique on a *Bruker maXis 4G* mass spectrometer. Sonication was performed in a *VWR* Ultrasonic Cleaner USC TH. Reactions under microwave irradiation were performed in an *Anton Paar Monowave 400* reactor.

Source of chemicals: Anhydrous CH_2Cl_2, Et_2O, and THF were taken from a solvent drying system (*MBraun* SPS-800). $CDCl_3$ (99.8%) was purchased from *Deutero GmbH*, *Sigma-Aldrich*, and *Cambridge Isotope Laboratories*. Acetone-d_6 (99.9%) was purchased from Cambridge Isotope Laboratories. Iron(III) oxalate hexahydrate, sodium borohydride, sodium borodeuteride, diethylaminosulfur trifluoride, anhydrous acetonitrile, 2,6-dimethyl-5-heptenal, benzyl bromide, camphene, 2-methyl-4-phenylbutan-2-ol, dimethoxyethane, ethyl 4-acetylbutyrate, methyllithium solution in Et_2O, *n*-butyllithium solution in hexane, titanium tetrachloride, 6-methylhept-5-en-2-on, methylmagnesium bromide solution in Et_2O, 5-hexenol, acetophenone, and 4-biphenylmethanol were purchased from *Sigma-Aldrich*. 2-(3-hydroxy-3-methylbutyl)phenol and Selectfluor were purchased from *Apollo Scientific.* 2-methyl-1-phenyl-2-propanol was purchased from *J & K Scientific.* Sodium hydride, sodium sulfate, sodium bicarbonate, ammonium chloride, CH_2Cl_2 (HPLC), and EtOAc (HPLC) were purchased from *VWR*. Ammonia solution in water and EtOH were purchased from *J.T. Baker*. Acetonitrile (HPLC) was purchased from *Macron*. Pentane and Et_2O were purchased from *Biosolve* and distilled prior to use. Silica gel (0.040-0.063 mm, 230-400 mesh ASTM) was purchased from *Silicycle*. Chemicals were used without further purification, unless stated otherwise.

General: Transfer of liquids with a volume ranging from 1 to 10 µL or from 10 to 100 µL was performed with a *Microman M1* pipette (*Gilson*, systematic error: 1.40% - 1.60%) equipped with 10 µL or 100 µL pipette tips respectively. The weighing of tetrabutylammonium bromide, hexafluorobenzene, solid substrates, and substrates with unknown density for the preparation of stock solutions was performed using a AB135-S/FACT *Mettler-Toledo* microbalance.

2. Synthesis of *C*-Undecylcalix[4]resorcinarene (**1**)

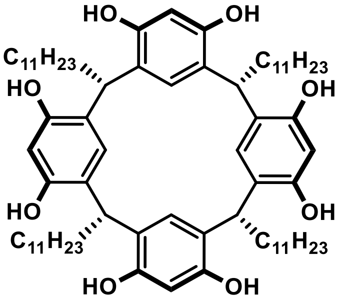

$C_{72}H_{112}O_8$
1105.68 g/mol

Resorcin[4]arene **1** was synthesized according to a modified literature procedure.[1] To a stirred solution of 99.9% ethanol (270 mL) and 37% aqueous HCl (90 mL), resorcinol (70.9 g, 644 mmol, 1.0 eq.) was added. After complete dissolution and cooling to 0 °C, a solution of dodecanal (143 mL, 119 g, 644 mmol, 1.0 eq.) in 99.9% ethanol (180 mL) was added dropwise into the reaction mixture over the course of 40 min. The resulting solution was allowed to warm to r.t. and subsequently refluxed at 100 °C for 18 h. Upon cooling to r.t. a yellow precipitate formed from the dark red solution. The precipitate was dispersed in cold methanol, filtered, and subsequently washed with cold methanol until the washings were light yellow. The solid was recrystallized from methanol (150 mL). To remove remaining yellow impurities the solid was washed extensively with a mixture of methanol/water (50/50, 8 × 100 mL). The crystalline material was dried under reduced pressure (15 mbar) at 55 °C using a rotary evaporator. The drying process was continued until the residual methanol was completely removed. In order to obtain a satisfying water content the material was moistened with cold methanol, washed with water (8 × 100 mL), and dried under reduced pressure at 55 °C. Compound **1** (109 g, 98.5 mmol, 61%) was obtained as a white to slightly yellowish powder. After dissolving **1** (11.0 mg) in $CDCl_3$ (0.5 mL), a water content of 13.5 eq. H_2O/hexamer was determined *via* integration of the ^1H NMR spectrum. The spectroscopic data matched those reported in the literature.[1]

3. Substrate syntheses

Compounds **10** and **17** were prepared according to published procedures.[2] The spectroscopic data were in accordance with values found in literature.[2]

General procedure A for the deoxyfluorination of secondary and tertiary alcohols:

According to a modified literature procedure,[3] diethylaminosulfur trifluoride (1.1 eq.) was dissolved in anhydrous CH_2Cl_2 (0.15 M) and cooled to – 78 °C. A solution of the alcohol (1.0 eq.) in CH_2Cl_2 (0.4 M) was then added dropwise and the reaction mixture was stirred for 1 h at – 78 °C. The reaction was quenched by the addition of saturated aqueous $NaHCO_3$ and extracted with CH_2Cl_2 (3 ×). The combined organic phases were dried over Na_2SO_4, filtered and concentrated under reduced pressure. The crude product was subsequently purified *via* flash column chromatography (pentane/Et_2O).

General procedure B for the hydrofluorination of alkenes:

According to a modified literature procedure,[2a] iron oxalate hexahydrate (2.0 eq.) was dissolved in water (0.025 M) over the course of 3-4 h, cooled to 0 °C and degassed by bubbling Argon through the solution. A solution of Selectfluor (2.0 eq.) and substrate in MeCN (0.025 M) was added to the reaction mixture and stirred for 10 min. $NaBH_4$ (6.4 eq.) was added in two portions over the course of 5 min. After additional stirring (25 min) the reaction mixture was quenched by the addition of aqueous NH_3 (25%) and extracted with CH_2Cl_2 (3 ×). The combined organic phases were dried over Na_2SO_4, filtered, and concentrated under reduced pressure. The crude product was subsequently purified *via* flash column chromatography (pentane/Et_2O).

(3-Fluoro-3-methylbutyl)benzene (3)

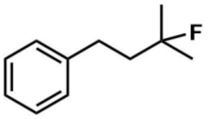

$C_{11}H_{15}F$
166.24 g/mol

According to general procedure A, 2-methyl-4-phenylbutan-2-ol (250 mg, 1.52 mmol, 1.0 eq.) was reacted with diethylaminosulfur trifluoride (220 µL, 269 mg, 1.67 mmol, 1.1 eq.) at – 78 °C for 1 h. After flash column chromatography [SiO_2, pentane/Et_2O = 50/1] fluoride **6** (250 mg, 1.50 mmol, *99%*) was obtained as a colorless oil.

TLC: R_f = 0.56 (pentane) [$KMnO_4$].

^{1}H NMR (500 MHz, $CDCl_3$): δ[ppm] = 7.34 – 7.26 (m, 2H), 7.24 – 7.16 (m, 3H), 2.79 – 2.69 (m, 2H), 2.01 – 1.85 (m, 2H), 1.42 (d, ^3J = 21.5 Hz, 6H).

^{13}C NMR (101 MHz, $CDCl_3$): δ[ppm] = 142.2, 128.6, 128.4, 126.0, 95.5 (d, ^1J = 165.6 Hz), 43.5 (d, ^2J = 22.9 Hz), 30.4 (d, ^3J = 5.3 Hz), 26.8 (d, ^2J = 24.8 Hz).

^{19}F NMR (235 MHz, $CDCl_3$): δ[ppm] = – 139.4.

HRMS (EI): 166.1158, found: 166.1147.

IR (ATR): $\tilde{\nu}$ [cm^{-1}] = 3028, 2981, 2938, 1605, 1495, 1455, 1373, 1254, 1211, 1181, 1137, 1073, 884, 765, 738.

(2-Fluoro-2-methylpropyl)benzene (4)

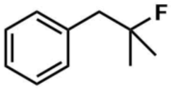

$C_{10}H_{13}F$
152.21 g/mol

According to general procedure A, 2-methyl-1-phenylpropan-2-ol (200 mg, 1.33 mmol, 1.0 eq.) was reacted with diethylaminosulfur trifluoride (193 µL, 235 mg, 1.46 mmol, 1.1 eq.). After flash column chromatography [SiO_2, pentane/Et_2O = 50/1] fluoride **7** (153 mg, 1.01 mmol, *76%*) was obtained as a colorless oil.

TLC: R_f = 0.39 (pentane) [UV, $KMnO_4$].

^{1}H NMR (400 MHz, $CDCl_3$): δ[ppm] = 7.37 – 7.16 (m, 5H), 2.92 (d, ^3J = 20.5 Hz, 2H), 1.34 (d, ^3J = 21.3 Hz, 6H).

^{19}F NMR (376 MHz, $CDCl_3$): δ[ppm] = – 137.0.

Spectroscopic data were in accordance with values found in literature.[4]

Synthesis of ester 5:

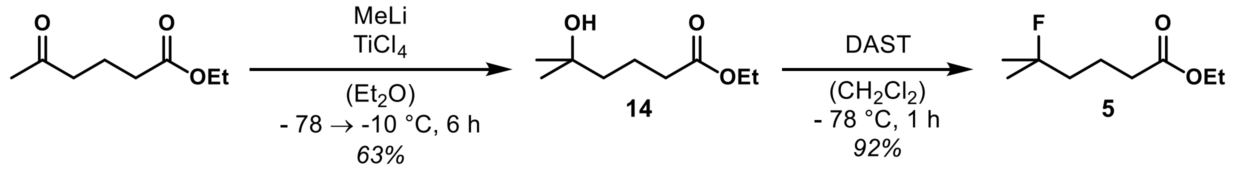

SI-Scheme 1. Synthesis of ester **5**.

Ethyl 5-hydroxy-5-methylhexanoate (14)

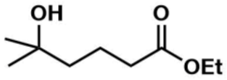

$C_9H_{18}O_3$
174.24 g/mol

According to a modified literature procedure,[5] methyllithium (1.6 M, 2.17 mL, 3.48 mmol, 1.1 eq.) was added slowly to a suspension of $TiCl_4$ (659 mg, 3.48 mmol, 1.1 eq.) in Et_2O (16 mL) at – 78 °C. After warming the suspension to – 30 °C, ethyl 4-acetylbutyrate (500 mg, 3.16 mmol, 1.0 eq.) was added dropwise. The reaction mixture was allowed to warm to – 10 °C and stirred for 6 h. The reaction was quenched by pouring the mixture onto water (30 mL), followed by extraction with Et_2O (3 ×). The combined organic phases were dried over Na_2SO_4, filtered and concentrated under reduced pressure. After flash column chromatography [SiO_2, pentane/EtOAc = 3/2] ester **14** (349 mg, 2.01 mmol, *63%*) was obtained as a colorless oil.

TLC: R_f = 0.36 (pentane/EtOAc = 2/1) [$KMnO_4$].

^{1}H NMR (400 MHz, $CDCl_3$): δ[ppm] = 4.13 (q, ^3J = 7.1 Hz, 2H), 2.32 (t, ^3J = 7.4 Hz, 2H), 1.80 – 1.63 (m, 2H), 1.52 – 1.45 (m, 2H), 1.32 (s, 1H), 1.25 (t, ^3J = 7.1 Hz, 3H), 1.22 (s, 6H).

^{13}C NMR (101 MHz, $CDCl_3$): δ[ppm] = 173.8, 70.9, 60.4, 43.3, 34.7, 29.4 (2 × CH_3), 20.0, 14.4.

HRMS (EI): calc. for $C_8H_{15}O_3^+$ [$(M-CH_3)^+$]: 159.1021, found: 159.1013.

IR (ATR): $\tilde{\nu}$ [cm^{-1}] = 3445, 2970, 1733, 1466, 1373, 1259, 1184, 1128, 1028, 944, 905.

Ethyl 5-fluoro-5-methylhexanoate (5)

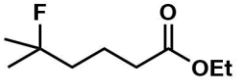

$C_9H_{17}FO_2$
176.23 g/mol

According to general procedure A, ester **14** (155 mg, 0.89 mmol, 1.0 eq.) was reacted with diethylaminosulfur trifluoride (129 µL, 157 mg, 0.98 mmol, 1.1 eq.) at – 78 °C for 1 h. After flash column chromatography [SiO_2, pentane/EtOAc = 20/1], fluoride **5** (143 mg, 0.81 mmol, *91%*) was obtained as a colorless oil.

TLC: R_f = 0.22 (pentane/EtOAc = 20/1) [$KMnO_4$].

^{1}H NMR (400 MHz, $CDCl_3$): δ[ppm] = 4.13 (q, ^3J = 7.1 Hz, 2H), 2.32 (t, ^3J = 7.2 Hz, 2H), 1.80 – 1.55 (m, 4H), 1.35 (d, ^3J = 21.4 Hz, 6H), 1.25 (t, ^3J = 7.1 Hz, 3H).

^{13}C NMR (101 MHz, $CDCl_3$): δ[ppm] = 173.4, 95.4 (d, ^1J = 165 Hz), 60.3, 40.7 (d, ^2J = 23.1 Hz), 34.4, 26.6 (d, ^2J = 24.8 Hz, 2 × CH_3), 19.5 (d, ^3J = 5.3 Hz), 14.3.

^{19}F NMR (376 MHz, $CDCl_3$): δ[ppm] = – 138.2

HRMS (EI): calc. for $C_9H_{16}O_2^+$ [$(M\text{-}HF)^+$]: 156.1150, found: 156.1142.

IR (ATR): $\tilde{\nu}$ [cm^{-1}] = 2982, 2939, 1734, 1462, 1374, 1262, 1185, 1129, 1029, 864, 763.

Synthesis of alkene 6:

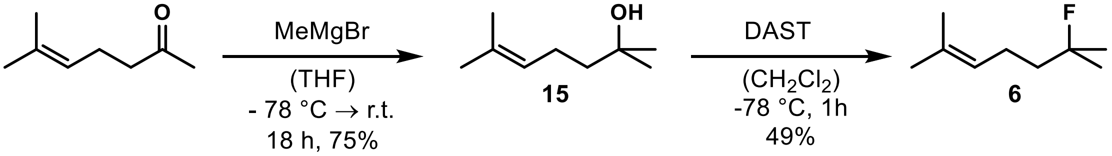

SI-Scheme 2. synthesis of alkene **6**.

2,6-Dimethylhept-5-en-2-ol (15)

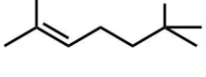

$C_9H_{18}O$
142.24 g/mol

According to a modified literature procedure,[6] 6-methylhept-5-en-2-one (626 mg, 4.97 mmol, 1.0 eq.) was dissolved in THF (13 mL) and cooled to – 78 °C. MeMgBr solution (3.0 M, 3.70 mL, 3.85 g, 10.9 mmol, 2.2 eq.) was added dropwise to the solution. After warming to r.t. over 16 h,

the reaction mixture was carefully quenched by addition of saturated aqueous NH_4Cl and extracted with EtOAc (3 ×). The combined organic phases were dried over Na_2SO_4, filtered, and concentrated under reduced pressure. Without further purification alcohol **15** (530 mg, 3.73 mmol, 75%) was obtained as a colorless oil.

TLC: R_f = 0.34 (pentane/EtOAc = 10/1) [$KMnO_4$].

^{1}H NMR (400 MHz, $CDCl_3$): δ[ppm] = 5.13 (m, 1H), 2.12 – 2.01 (m, 2H), 1.69 (s, 3H), 1.62 (s, 3H), 1.55 – 1.44 (m, 2H), 1.22 (s, 6H).

Spectroscopic data were in accordance with values found in literature.[7]

6-fluoro-2,6-dimethylhept-2-ene (6)

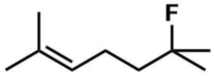

$C_9H_{17}F$
144.23 g/mol

According to general procedure A, alcohol **15** (300 mg, 2.11 mmol, 1.0 eq.) was reacted with diethylaminosulfur trifluoride (307 µL, 374 mg, 2.32 mmol, 1.1 eq.) at – 78 °C for 1 h. After column chromatography [SiO_2, pentane] fluoride **6** (149 mg, 1.03 mmol, 49%) was obtained as a colorless oil.

TLC: R_f = 0.66 (pentane) [$KMnO_4$].

^{1}H NMR (400 MHz, $CDCl_3$): δ[ppm] = 5.16 – 5.04 (m, 1H), 2.14 – 2.00 (m, 2H), 1.69 (s, 3H), 1.61 (s, 3H), 1.71 – 1.51 (m, 2H), 1.35 (d, ^3J = 21.5 Hz, 6H).

^{13}C NMR (101 MHz, $CDCl_3$): δ[ppm] = 131.9, 124.1, 95.8 (d, ^1J = 165 Hz), 41.5 (d, ^2J = 22.7 Hz), 26.8 (d, ^2J = 24.8 Hz, 2 × CH_3), 25.8, 22.8 (d, ^3J = 5.7 Hz), 17.7.

^{19}F NMR (235 MHz, $CDCl_3$): δ[ppm] = – 138.3.

HRMS (EI): 144.1314, found: 144.1308.

IR (ATR): $\tilde{\nu}$ [cm^{-1}] = 2979, 2928, 2861, 1453, 1374, 1259, 1203, 1181, 1141, 888, 844, 760.

2-Fluoro-2,3,3-trimethylbicyclo[2.2.1]heptane (7)

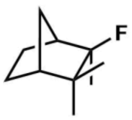

$C_{10}H_{17}F$
156.24 g/mol

According to general procedure B, camphene (200 mg, 1.47 mmol, 1.0 eq.) was reacted with iron(III) oxalate hexahydrate (1422 mg, 2.94 mmol, 2.0 eq.), Selectfluor (1042 mg, 2.94 mmol, 2.0 eq.), and $NaBH_4$ (356 mg, 9.41 mmol, 6.4 eq.). The reaction mixture was quenched with aqueous NH_3 (25%) and extracted with pentane (3 ×). The combined organic phases were dried over Na_2SO_4, filtered and concentrated under reduced pressure. Fluoride **7** (167 mg, 1.07 mol, 73%) was obtained without further purification as colorless needles.

TLC: R_f = 0.57 (pentane) [CAM].

^{1}H NMR (500 MHz, $CDCl_3$): δ[ppm] = 2.17 (ddt, J = 7.2, 3.2, 1.6 Hz, 1H), 2.09 – 1.98 (m, 1H), 1.77 (dq, J = 2.6, 1.5 Hz, 1H), 1.54 – 1.44 (m, 2H), 1.26 (d, J = 24.5 Hz, 3H), 1.29 – 1.19 (m, 2H), 1.10 (dt, J = 10.0, 1.5 Hz, 1H), 0.99 (d, J = 6.4 Hz, 3H), 0.89 (d, J = 1.0 Hz, 3H).

^{13}C NMR (126 MHz, $CDCl_3$): δ[ppm] = 105.1 (d, ^1J = 181.3 Hz), 49.5 (d, ^2J = 23.5 Hz), 49.0, 34.5, 24.1, 23.9 (2 × CH_3), 23.6 (d, ^3J = 2.6 Hz), 22.9 (d, ^3J = 9.0 Hz), 18.1 (d, ^2J = 29.7 Hz).

^{19}F NMR (376 MHz, CDCl3): δ[ppm] = – 134.1.

HRMS (EI): 156.1314, found: 156.1312.

IR (ATR): $\tilde{\nu}$ [cm^{-1}] = 2963, 2883, 1463, 1377, 1320, 1171, 1118, 1073, 1035, 900, 874, 718.

6-Fluoro-2,6-dimethylheptan-1-ol (8)

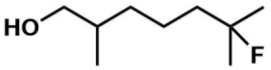

$C_9H_{19}FO$
162.25 g/mol

According to general procedure B, 2,6-dimethyl-5-heptenal (200 mg, 1.43 mmol, 1.0 eq.) was reacted with iron(III) oxalate hexahydrate (1384 mg, 2.86 mmol, 2.0 eq.), Selectfluor (1013 mg, 2.86 mmol, 2.0 eq.) and $NaBH_4$ (346 mg, 9.15 mmol, 6.4 eq.). After flash column chromatography [SiO_2, pentane/Et2O = 3/1], alcohol **8** (95.9 mg, 0.59 mmol, 41%) was obtained as a colorless oil.

TLC: R_f = 0.30 (pentane/EtOAc = 10/1) [$KMnO_4$].

^{1}H NMR (500 MHz, $CDCl_3$): δ[ppm] = 3.55 – 3.39 (m, 2H), 1.70 – 1.54 (m, 3H), 1.52 – 1.39 (m, 3H), 1.34 (d, ^3J = 21.5 Hz, 6H), 1.17 – 1.07 (m, 1H), 0.93 (d, ^3J = 6.7 Hz, 3H).

^{13}C NMR (101 MHz, $CDCl_3$): δ[ppm] = 95.9 (d, ^1J = 164.3 Hz), 68.4, 41.8 (d, ^2J = 22.8 Hz), 35.9, 33.5, 26.9 (d, ^2J = 10.4 Hz), 26.7 (d, ^2J = 10.4 Hz), 21.5 (d, ^3J = 5.1 Hz), 16.7.

^{19}F NMR (376 MHz, $CDCl_3$): δ[ppm] = -138.0.

HRMS (EI): calc. for $C_9H_{18}O^+$ [$(M\text{-}HF)^+$]: 142.1358, found: 142.1355.

IR (ATR): $\tilde{\nu}$ [cm^{-1}] = 3570, 3355, 2978, 2942, 2874, 1464, 1374, 1255, 1212, 1146, 1038, 935, 884, 758.

Synthesis of ether 9:

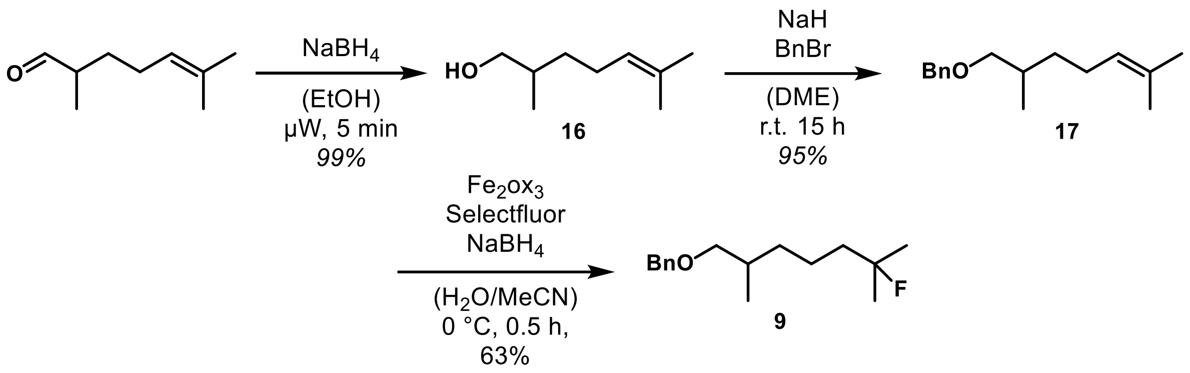

SI-Scheme 3. Synthesis of ether **9**.

2,6-Dimethylhept-5-en-1-ol (16)

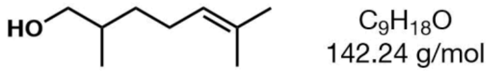

To a suspension of $NaBH_4$ (27.0 mg, 0.72 mmol, 0.5 eq.) in 4.6 mL EtOH 2,6-dimethyl-5-heptenal (260 mg, 1.85 mmol, 1.0 eq.) was added. The suspension was then heated to 100 °C in a microwave reactor and stirred for 5 min. After cooling to r.t. the reaction mixture was quenched by addition of saturated aqueous $NaHCO_3$ and extracted with CH_2Cl_2 (3 ×). The combined organic phases were dried over Na_2SO_4, filtered, and concentrated under reduced pressure. Alcohol **16** (261 mg, 1.83 mmol, *99%*) was obtained without further purification as a colorless oil.

TLC: R_f = 0.69 (pentane/EtOAc = 3/1) [$KMnO_4$].

^{1}H NMR (400 MHz, $CDCl_3$): δ[ppm] = 5.10 (m, 1H), 3.55 – 3.38 (m, 2H), 2-14 – 1.89 (m, 2H), 1.68 (s, 3H), 1.66 – 1.62 (m, 1H), 1.61 (s, 3H), 1.52 – 1.38 (m, 1H), 1.17 – 1.09 (m, 1H), 0.93 (d, ^3J = 6.8 Hz, 3H).
Spectroscopic data were in accordance with values found in literature.[8]

(((2.6-dimethylhept-5-en-1-yl)oxy)methyl)benzene (17)

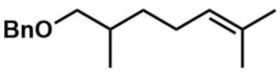

$C_{16}H_{24}O$
232.18 6/mol

According to a literature procedure,[2b] NaH (60% suspension in mineral oil, 73.3 mg, 1.83 mmol, 1.3 eq.) was suspended in 1.5 mL Dimethoxyethane (DME). Alcohol **16** (200 mg, 1.41 mmol, 1.0 eq.) was added dropwise as a solution in 1.5 mL DME. The reaction mixture was stirred for 10 min at r,t,, then benzyl bromide (289 mg, 1.69 mmol, 1.2 eq.) was added. As soon as gas formation ceased the mixture was heated to reflux for three hours and subsequently stirred at r.t. for 15 hours. The reaction was quenched by addition of aq. HCl (1M, 5 mL). After separation of the layers the aqueous phase was washed with Et_2O (3 × 10 mL) and the combined organic phases were dried over Na_2SO_4, filtered and concentrated under reduced pressure. After flash column chromatography [SiO_2, pentane/EtOAc = 70/1] ether **17** (310 mg, 1.34 mmol, 95%] was obtained as a colorless oil.

TLC: R_f = 0.97 (pentane/EtOAc = 10/1) [$KMnO_4$].

^{1}H NMR (400 MHz, $CDCl_3$): δ[ppm] = 7.40 – 7.32 (m, 4H), 7.31 – 7.27 (m, 1H), 5.14 – 5.07 (m, 1H), 4.50 (s, 2H), 3.37 – 3.22 (m, 2H), 2.11 – 1.90 (m, 2H), 1.85 – 1.73 (m, 1H), 1.68 (s, 3H), 1.60 (s, 3H), 1.53 – 1.41 (m, 1H), 1.22 – 1.09 (m, 1H), 0.95 (d, ^3J = 6.7 Hz, 3H).

Spectroscopic data were in accordance with values found in literature.[2b]

(((6-Fluoro-2,6-dimethylheptyl)oxy)methyl)benzene (9)

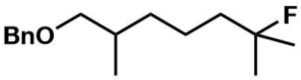

$C_{16}H_{25}FO$
252.37 g/mol

According to general procedure B, ether **17** (100 mg, 0.43 mmol, 1.0 eq.) was reacted with iron(III) oxalate hexahydrate (416 mg, 0.86 mmol, 2.0 eq.), Selectfluor (321 mg, 0.86 mmol, 2.0 eq.) and $NaBH_4$ (104 mg, 2.75 mmol, 6.4 eq.). After flash column chromatography [SiO_2, pentane/EtOAc = 50/1] fluoride **9** (68.9 mg, 0.273 mmol, *63%*) was obtained as a colorless oil.

TLC: R_f = 0.32 (pentane/EtOAc = 50/1) [UV, $KMnO_4$].

^{1}H NMR (400 MHz, $CDCl_3$): δ[ppm] = 7.40 – 7.26 (m, 5H), 4.50 (s, 2H), 3.37 – 3.22 (m, 2H), 1.86 – 1.71 (m, 1H), 1.67 – 1.51 (m, 3H), 1.51 – 1.39 (m, 2H), 1.33 (d, ^{3}J = 21.5 Hz, 6H), 1.18 – 1.08 (m, 1H), 0.94 (d, ^{3}J = 6.7 Hz, 3H).

^{13}C NMR (101 MHz, $CDCl_3$): δ[ppm] = 138.9, 128.5, 127.7, 127.6, 95.9 (d, ^{1}J = 164.0 Hz), 76.0, 73.1, 41.8 (d, ^{2}J = 22.8 Hz), 34.1, 33.6, 26.9 (d, ^{2}J = 10.6 Hz), 26.7 (d, ^{2}J = 10.5 Hz), 21.5 (d, ^{3}J = 5.3 Hz), 17.2.

^{19}F NMR (376 MHz, $CDCl_3$): δ[ppm] = – 137.8.

HRMS (EI): calc. for $C_{16}H_{24}O^+$ [$(M\text{-}HF)^+$]: 232.1827, found: 232.1816.

IR (ATR): $\tilde{\nu}$ [cm^{-1}] = 2941, 2855, 1455, 1373, 1255, 1208, 1097,886, 735, 697.

7-Fluoro-3,7-dimethyloctan-1-ol (**10**)

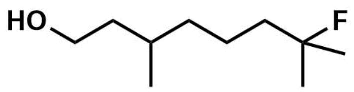

$C_{10}H_{21}FO$
176.16 g/mol

According to general procedure B, β-citronellol (300 mg, 1.92 mmol, 1.0 eq.) was reacted with iron(III) oxalate hexahydrate (1.86 g, 3.84 mmol, 2.0 eq.), Selectfluor (1.43 g, 3.84 mmol, 2.0 eq.) and $NaBH_4$ (484 mg, 12.3 mmol, 6.4 eq.). After flash column chromatography [SiO_2, pentane/EtOAc = 3/1] fluoride **10** (78.1 mg, 0.44 mmol, *23%*) was obtained as a colorless oil.

TLC: R_f = 0.46 (pentane/EtOAc = 3/1) [UV, $KMnO_4$].

^{1}H NMR (400 MHz, $CDCl_3$): δ[ppm] = 3.77 – 3.63 (m, 2H), 1.66 – 1.49 (m, 4H), 1.50 – 1.20 (m, 5H), 1.36 (s, 3H), 1.31 (s, 3H), 1.27 – 1.10 (m, 1H), 0.91 (d, ^{3}J = 6.5 Hz, 3H).

Spectroscopic data were in accordance with values found in literature.[2a]

1-(*tert*-butyl)-4-(fluoromethyl)benzene (11)

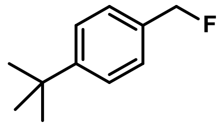

$C_{11}H_{15}F$
166.12 g/mol

According to general procedure A, (4-(*tert*-butyl)phenyl)methanol (500 mg, 3.04 mmol, 1.0 eq.) was reacted with diethylaminosulfur trifluoride (539 mg, 3.34 mmol, 1.1 eq.) at – 78 °C for 3 h.

After column chromatography [SiO_2, pentane] fluoride **3** (420 mg, 2.53 mmol, 83%) was obtained as a colorless oil.

TLC: R_f = 0.46 (pentane/EtOAc = 3/1) [UV, $KMnO_4$].

^{1}H NMR (400 MHz, $CDCl_3$): δ[ppm] = 7.47 – 7.39 (m, 2H), 7.37 – 7.29 (m, 2H), 5.35 (d, ^2J = 48.1 Hz, 2H), 1.34 (s, 9H).

Spectroscopic data were in accordance with values found in literature.[9]

3.1 Unreactive substrates

5-Fluorohexanol (18)

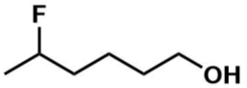

$C_6H_{13}FO$
120.17 g/mol

According to general procedure B, 5-hexenol (200 mg, 2.00 mmol, 1.0 eq.) was reacted with iron(III) oxalate hexahydrate (1.50 g, 3.99 mmol, 2.0 eq.), Selectfluor (1.41 g, 3.99 mmol, 2.0 eq.), and $NaBH_4$ (483 mg, 12.8 mmol, 6.4 eq.) in water/MeCN (12.5 mM) at 0 °C for 30 min. After flash column chromatography [SiO_2, pentane/Et_2O = 2/1] 5-fluorohexanol (**18**, 136 mg, 1.13 mmol, 57%) was obtained as a colorless oil.

TLC: R_f = 0.29 (pentane/EtOAc = 1/1) [$KMnO_4$].

^{1}H NMR (400 MHz, $CDCl_3$): δ[ppm] = 4.78 – 4.54 (m, 1H), 3.66 (t, ^3J = 6.4 Hz, 2H), 1.78 – 1.37 (m, 6H), 1.32 (dd, ^3J = 24.0, 6.2 Hz, 3H).

Spectroscopic data were in accordance with values found in literature.[10]

2-(3-fluoro-3-methylbutyl)phenol (19)

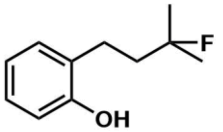

$C_{11}H_{15}FO$
182.24 g/mol

According to general procedure A, 2-(3-hydroxy-3-methylbutyl)phenol (100 mg, 0.56 mmol, 1.0 eq.) was reacted with diethylaminosulfur trifluoride (80.7 µL, 98.5 mg, 0.61 mmol, 1.1 eq.) in

CH_2Cl_2 (0.1 M) at – 78 °C for 1 h. After flash column chromatography [SiO_2, pentane/Et_2O = 5/1], phenol **19** (50.9 mg, 0.28 mmol, 50%) was obtained as a colorless oil.

TLC: R_f = 0.94 (pentane/EtOAc = 2/1) [$KMnO_4$].

^{1}H NMR (400 MHz, $CDCl_3$): δ[ppm] = 7.17 – 7.05 (m, 2H), 6.87 (td, ^3J = 7.4 Hz, ^4J = 1.2 Hz, 1H), 6.76 (dd, ^3J = 8.0 Hz, ^4J = 1.2 Hz, 1H), 4.82 (s, 1H), 2.77 – 2.67 (m, 2H), 1.99 – 1.85 (m, 2H), 1.43 (d, ^3J = 21.6 Hz, 6H).

^{13}C NMR (101 MHz, $CDCl_3$): δ[ppm] = 153.6, 130.2, 128.1, 127.5, 121.0, 115.5, 96.0 (d, ^1J = 165.0 Hz), 41.5 (d, ^2J = 22.8 Hz), 26.8 (d, ^2J = 24.7 Hz, 2 × CH_3), 24.6 (d, ^3J = 5.4 Hz).

^{19}F NMR (376 MHz, $CDCl_3$): δ[ppm] = – 139.5.

HRMS (EI): 182.1107, found: 182.1107.

IR (ATR): $\tilde{\nu}$ [cm^{-1}] = 2975, 2928, 2853, 1747, 1581, 1488, 1454, 1369, 1304, 1254, 1219, 1155, 1122, 1037, 947, 883, 833, 752.

Synthesis of nitrile 21:

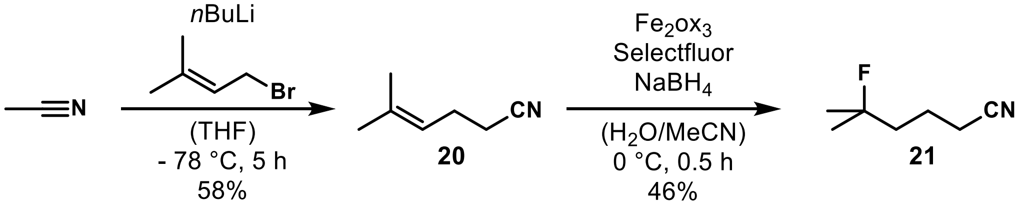

SI-Scheme 4. Synthesis of nitrile **20**.

5-methylhex-4-enenitrile (20)

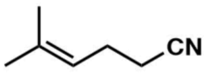

$C_7H_{11}N$
109.17 g/mol

According to a published procedure,[11] *n*-Butyllithium (2.5 M, 3.00 mL, 7.05 mmol, 1.05 eq.) was added dropwise to a solution of acetonitrile (289 mg, 7.05 mmol, 1.05 eq.) in THF (0.33 M) at – 78 °C. The solution was stirred for 10 min, then prenyl bromide (775 µL, 1000 mg, 6.71 mmol, 1.0 eq.) was added dropwise. After additional stirring for 4.5 h at – 78 °C, the reaction mixture was quenched by addition of saturated aqueous NH_4Cl, then extracted with Et_2O (3 ×). The combined

organic phases were dried over Na_2SO_4, filtered, and concentrated under reduced pressure. After flash column chromatography [SiO_2, pentane/Et_2O = 10/1], nitrile **20** (423 mg, 3.88 mmol, 58%) was obtained as a colorless liquid.

TLC: R_f = 0.63 (pentane/EtOAc = 10/1) [UV, $KMnO_4$].

^{1}H NMR (400 MHz, $CDCl_3$): δ[ppm] = 5.19 – 5.09 (m, 1H), 2.39 – 2.28 (m, 4H), 1.72 (d, ^3J = 1.3 Hz, 3H), 1.65 (d, ^4J = 1.3 Hz, 3H).

Spectroscopic data were in accordance with values found in literature.[11]

5-fluoro-5-methylhexanenitrile (21)

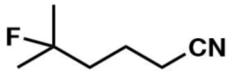

$C_7H_{12}FN$
129.18 g/mol

According to general procedure B, nitrile **20** (100 mg, 0.92 mmol, 1.0 eq.) was reacted with iron(III) oxalate hexahydrate (886 mg, 1.84 mmol, 2.0 eq.), Selectfluor (649 mg, 1.84 mmol, 2.0 eq.), and $NaBH_4$ (222 mg, 5.89 mmol, 6.4 eq.) in water/MeCN (74 mL) at 0 °C for 30 min. After flash column chromatography [SiO_2, pentane/EtOAc = 20/1], fluoride **21** (54.1 mg, 0.42 mmol, 46%) was obtained as colorless oil.

^{1}H NMR (400 MHz, $CDCl_3$): δ[ppm] = 2.44 – 2.34 (m, 2H), 1.91 – 1.68 (m, 4H), 1.37 (d, ^3J = 21.3 Hz, 6H).

^{13}C NMR (101 MHz, $CDCl_3$): δ[ppm] = 119.6, 95.0 (d, ^1J = 166.2 Hz), 40.1 (d, ^2J = 23.2 Hz), 26.8 (d, ^2J = 24.8 Hz, 2 × CH_3), 20.3 (d, ^3J = 4.4 Hz), 17.6.

Spectroscopic data were in accordance with values found in literature.[12]

(1,1-difluoroethyl)benzene (22)

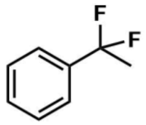

$C_8H_8F_2$
142.15 g/mol

According to a modified literature procedure,[13] bis(2-methoxyethyl)aminosulfur trifluoride (1.11 g, 5.00 mmol, 1.5 eq.) was added to neat acetophenone (400 mg, 3.33 mmol, 1.0 eq.), heated to 85 °C and stirred for 18 h. The reaction mixture was diluted with CH_2Cl_2, poured onto saturated aqueous $NaHCO_3$ and extracted with CH_2Cl_2 (3 ×). The combined organic phases were dried over Na_2SO_4, filtered, and concentrated under reduced pressure. After flash column chromatography [SiO_2, pentane/Et_2O = 10/1], difluoride **22** (72.4 mg, 0.51 mmol, 15%) was obtained as colorless liquid.

^{1}H NMR (400 MHz, $CDCl_3$): δ[ppm] = 7.56 – 7.48 (m, 2H), 7.48 – 7.31 (m, 3H), 1.93 (t, ^3J = 18.1 Hz, 3H).

^{19}F NMR (376 MHz, $CDCl_3$): δ[ppm] = -87.7.

Spectroscopic data were in accordance with values found in literature.[14]

3.2 Unselective substrates

4-(fluoromethyl)-1,1'-biphenyl (23)

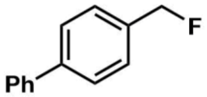

$C_{13}H_{11}F$
186.23 g/mol

According to a modified literature procedure,[13] bis(2-methoxyethyl)aminosulfur trifluoride (664 µL, 684 mg, 1.79 mmol, 1.1 eq.) was dissolved in CH_2Cl_2 (9 mL) and cooled to – 78 °C. (1,1'-biphenyl)-4-ylmethanol (300 mg, 1.63 mmol, 1.0 eq.) was dissolved in CH_2Cl_2 (8 mL) and added to the reaction mixture. The mixture was stirred for 3 h, then warmed to r.t. and quenched by addition of saturated aqueous $NaHCO_3$, and extracted with CH_2Cl_2 (3 ×). The combined organic phases were dried over Na_2SO_4, filtered and concentrated under reduced pressure. After flash column chromatography [SiO_2, pentane/EtOAc = 98/2], fluoride **23** (199 mg, 1.07 mmol, 65%) was obtained as a colorless solid.

TLC: R_f = 0.57 (pentane/EtOAc = 40/1) [UV, $KMnO_4$].

^{1}H NMR (400 MHz, $CDCl_3$): δ[ppm] = 7.66 – 7.57 (m, 4H), 7.49 (m, 4H), 7.40 – 7.34 (m, 1H), 5.43 (d, ^3J = 47.9 Hz, 2H).

^{19}F NMR (376 MHz, $CDCl_3$): δ[ppm] = – 206.2.

Spectroscopic data were in accordance with values found in literature.[15]

3.3 Synthesis of deuterated substrate 3d

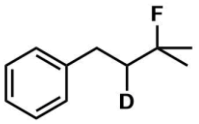

$C_{11}H_{14}DF$
167.12 g/mol

According to general procedure B, 3-Methyl-1-phenyl-but-2-ene (100 mg, 687 µmol, 1.0 eq.) was reacted with iron(III) oxalate hexahydrate (665 mg, 1.37 mmol, 2.0 eq.), Selectfluor (487 mg, 1.37 mmol, 2.0 eq.), and $NaBD_4$ (184 g, 4.40 mmol, 6.4 eq.) for 30 min at 0 °C. After flash column chromatography [SiO_2, pentane] (3-fluoro-3-methylbutyl-2-*d*)benzene (**3d**, 23.7 mg, 142 µmol, 21%) was obtained as a colorless liquid.

TLC: R_f = 0.28 (pentane) [UV, $KMnO_4$].

^{1}H NMR (400 MHz, $CDCl_3$): δ[ppm] = 7.33 – 7.27 (m, 2H), 7.24 – 7.16 (m, 3H), 2.72 (d, ^3J = 8.8 Hz, 2H), 2.00 – 1.82 (m, 1H), 1.42 (d, ^3J = 21.4 Hz, 6H).

^{13}C NMR (101 MHz, $CDCl_3$): δ[ppm] = 142.2, 128.6 (2 C), 128.4, 126.0, 95.4 (d, ^{1}J = 165.6 Hz), 43.1 (m), 30.3 (d, ^{3}J = 5.4 Hz), 26.8 (dd, ^{2}J = 24.8 Hz, ^{4}J = 3.0 Hz).

^{19}F NMR (235 MHz, $CDCl_3$): δ[ppm] = – 139.6 (t, ^3J = 3.1 Hz).

3.4 Synthesis of products unknown to literature

2-(4-*tert*-butyl)benzyl)1,3,5-trimethoxybenzene (13)

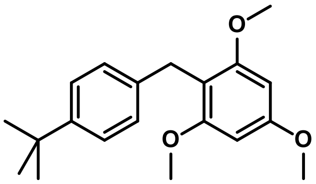

$C_{20}H_{26}O_3$
314.19 g/mol

According to Paquin *et al.*,[16] benzyl fluoride **3** (50 mg, 0.30 mmol, 1.0 eq.) was added to a solution of 1,3,5-Trimethoxybenzene (256 mg, 1.51 mmol, 5.0 eq.) in CH_2Cl_2/hexafluoroisopropanol (9/1, 6 mL) and stirred at r.t. for 18 hours. The reaction mixture was quenched by addition of H_2O, extracted with CH_2Cl_2 (3 ×), washed with brine (1 ×) and dried over Na_2SO_4. After removal of solvents under reduced pressure, column chromatography [SiO_2, toluene] gave benzylated product **13** (76.6 mg, 0.24 mmol, 81%) as a colorless solid.

TLC: R_f = 0.70 (PhMe) [UV].

^{1}H NMR (400 MHz, $CDCl_3$): δ[ppm] = 7.25 – 7.21 (m, 2H), 7.20 – 7.13 (m, 2H), 6.15 (s, 2H), 3.90 (s, 2H), 3.80 (s, 3H), 3.79 (s, 6H), 1.27 (s, 9H).

^{13}C NMR (101 MHz, $CDCl_3$): δ[ppm] = 159.7, 159.0 (2 C), 147.9, 139.3, 128.2 (2 C), 125.0 (2 C), 110.6, 90.8 (2 C), 55.9 (2 C), 55.5, 34.4, 31.6 (3 × CH_3), 27.9.

HRMS (EI): calc. for $C_{20}H_{27}O_3^+$ [$(M+H)^+$]: 315.1955, found: 315.1952.

IR (ATR): $\tilde{\nu}$ [cm^{-1}] = 2948, 1597, 1499, 1463, 1416, 1323, 1269, 1235, 1208, 1192, 1151, 1123, 1064, 1043, 952, 838, 818, 797, 779, 663, 629.

3.5 Synthesis of large substrate **28**

4,4''-di-tert-butyl-5'-(((6-fluoro-2,6-dimethylheptyl)oxy)methyl)-1,1':3',1''-terphenyl (27)

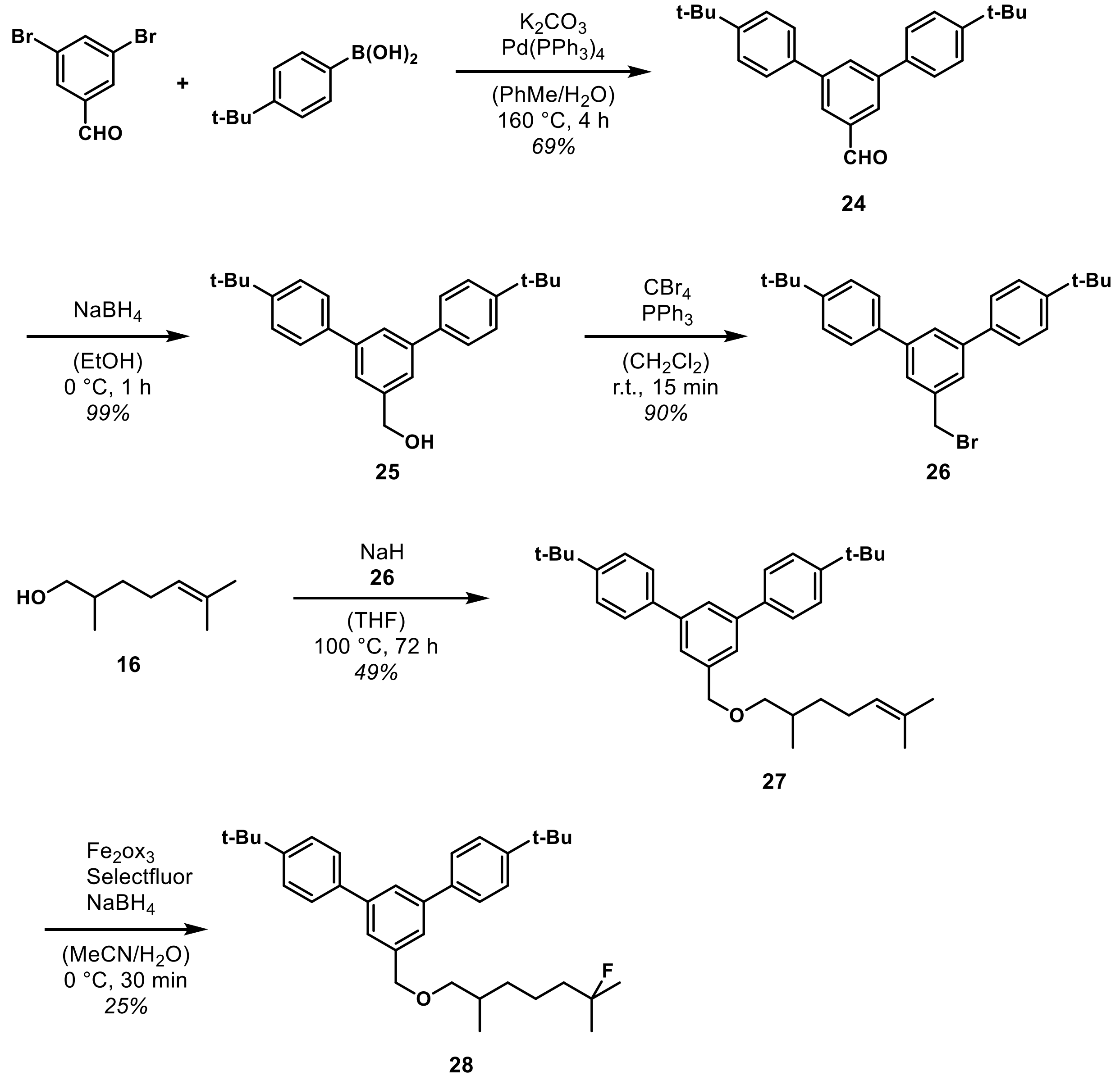

SI-Scheme 5. Synthesis of ether **28**.

4,4’’-di-tert-butyl-[1,1’:3’,1’’-terphenyl]-5’-carbaldehyde (24)

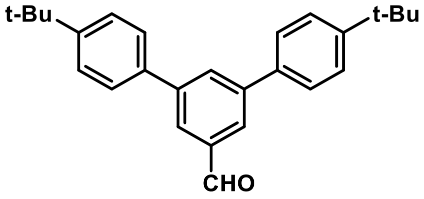

$C_{27}H_{30}O$
370.54 g/mol

According to a modified literature procedure,[Schanze2011 [17]] 3,5-Dibromobenzaldehyde (1.50 g, 5.68 mmol, 1.0 eq.), 4-tert-butylphenylboronic acid (2.64 g, 14.8 mmol, 2.6 eq.), and potassium carbonate (1.02 g, 7.41 mmol, 1.3 eq.) were dissolved in a toluene/water mixture (19 mL, 15:4) and degassed for 10 minutes. Tetrakis(triphenylphosphine)palladium (198 mg, 0.17 mmol, 0.03 eq.) was added and the mixture degassed for an additional 10 minutes. The reaction vial was transferred to a microwave and irradiated at 160 °C for 4 hours. After cooling to r.t. the reaction mixture was diluted with CH2Cl2 (40 mL) and water (20 mL), and extracted with CH2Cl2 (3 ×). The organic phases were washed with brine (100 mL) and dried over Na2SO4. After removal of the solvent under reduced pressure, the residue was dissolved in a minimal amount of CH2Cl2, to which MeOH was added dropwise. The resulting precipitate was filtered off and dried. Aldehyde **24** (1.46 g, 3.93 mmol, 69%) was obtained as colorless flakes that were used without further purification.

^{1}H NMR (500 MHz, $CDCl_3$): δ[ppm] = 10.14 (s, 1H), 8.08 – 8.03 (m, 3H), 7.65 – 7.60 (m, 4H), 7.55 – 7.49 (m, 4H), 1.39 (s, 18H).

^{13}C NMR (101 MHz, $CDCl_3$): δ[ppm] = 192.4, 151.3, 142.5, 137.4, 136.9, 131.5, 126.9, 126.8, 126.0, 34.7, 31.4.

Spectroscopic data were in accordance with values found in literature.[17]

(4,4''-di-tert-butyl-[1,1':3',1''-terphenyl]-5'-yl)methanol (25)

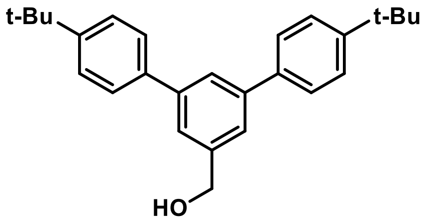

$C_{27}H_{32}O$
372.55 g/mol

According to a literature procedure,[18] aldehyde **24** (1.45 g, 3.91 mmol, 1.0 eq.) was dissolved in anhydrous EtOH (17 mL) and cooled to 0 °C. $NaBH_4$ (74 mg, 1.96 mmol, 0.5 eq.) was added in 2 portions to the reaction mixture and stirred for one hour. The reaction was quenched by addition of water (15 mL) and extracted with Et_2O. The organic phase was dried over Na_2SO_4 and the solvent removed under reduced pressure. Alcohol **25** (1.44 g, 3.89 mmol, 99%) was obtained as an off-white foam that was used without further purification.

^{1}H NMR (500 MHz, $CDCl_3$): δ[ppm] = 7.74 (t, ^3J = 1.8 Hz, 1H), 7.61 – 7.57 (m, 4H), 7.57 – 7.55 (m, 2H), 7.51 – 7.47 (m, 4H), 4.82 (d, ^3J = 4.8 Hz, 2H), 1.38 (s, 18H).

^{13}C NMR (101 MHz, $CDCl_3$): δ[ppm] = 150.7, 142.1, 141.9, 138.3, 127.0, 125.9, 125.4, 124.6, 65.7, 34.7, 31.5.

Spectroscopic data were in accordance with values found in literature.[18]

5'-(bromomethyl)-4,4''-di-tert-butyl-1,1':3',1''-terphenyl (26)

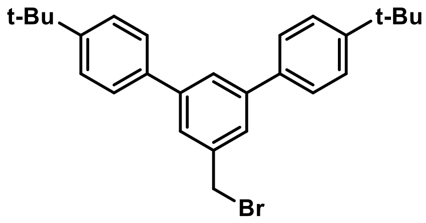

$C_{27}H_{31}Br$
435.45 g/mol

According to a literature procedure,[19] alcohol **25** (1.45 g, 3.89 mmol, 1.0 eq.) was dissolved in dry THF (8 mL), to which CBr_4 (3.23 g, 9.73 mmol, 2.5 eq.) and PPh_3 (2.55 g, 9.73 mmol, 2.5 eq.) were added. The reaction mixture was stirred for 15 min at r.t., after which it was poured onto a mixture of water/CH_2Cl_2 (20 mL, 1:1) and extracted with CH_2Cl_2 (3 ×). The organic phase was dried over Na_2SO_4 and the crude product purified *via* column chromatography [SiO2, pentane → pentane/CH_2Cl_2 = 2/1]. Bromide **26** (1.53 g, 3.51 mmol, *90%*) was obtained as a highly viscous colorless oil.

^{1}H NMR (500 MHz, $CDCl_3$): δ[ppm] = 7.76 – 7.71 (m, 1H), 7.60 – 7.54 (m, 6H), 7.52 – 7.46 (m, 4H), 4.71 (s, 1H), 4.61 (s, 1H), 1.38 (s, 18H).

^{13}C NMR (101 MHz, $CDCl_3$): δ[ppm] = 150.9, 142.3, 138.7, 137.9, 127.0, 126.6, 126.1, 126.0, 46.6, 34.7, 31.5.

Spectroscopic data were in accordance with values found in literature.[19]

4,4''-di-tert-butyl-5'-(((2,6-dimethylhept-5-en-1-yl)oxy)methyl)-1,1':3',1''-terphenyl (27)

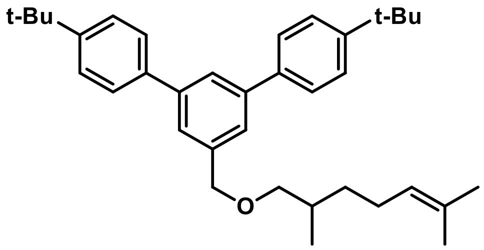

$C_{36}H_{48}O$
496.78 g/mol

NaH (60% dispersion in mineral oil, 67.7 mg, 1.69 mmol, 1.2 eq.) was dissolved in dry THF (12 mL) at r.t., to which alcohol **16** (200 mg, 1.41 mmol, 1.0 eq.) was added dropwise. After stirring for 10 min, a solution of bromide **26** (798 mg, 1.83 mmol, 1.3 eq.) in THF (2 mL) was added dropwise to the reaction mixture. The mixture was stirred for an additional one hour at r.t. and then heated to reflux for 72 hours. After cooling to r.t. sat. NH_4Cl solution (20 mL) was added to quench the reaction and extracted with CH_2Cl_2 (3 ×). The organic phase was separated, dried over Na_2SO_4, and the solvent removed under reduced pressure. Column chromatography [SiO_2, pentane/Et_2O = 49/1] gave ether **27** (346 mg, 0.69 mmol, 49%) as a colorless oil.

TLC: R_f = 0.43 (pentane/EtOAc = 70/1) [UV, $KMnO_4$].

^{1}H NMR (500 MHz, $CDCl_3$): δ[ppm] = 7.71 (t, ^4J = 1.8 Hz, 1H), 7.61 – 7.56 (m, 4H), 7.53 (d, ^4J = 1.8 Hz, 2H), 7.51 – 7.43 (m, 4H), 5.16 – 5.06 (m, 1H), 4.61 (s, 2H), 3.43 – 3.29 (m, 2H), 2.10 – 1.92 (m, 3H), 1.88 – 1.77 (m, 1H), 1.67 (s, 3H), 1.58 (s, 3H), 1.54 – 1.45 (m, 1H), 1.38 (s, 18H, 0.97 (d, ^3J = 6.6 Hz, 3H).

^{13}C NMR (126 MHz, $CDCl_3$): δ[ppm] = 150.5, 141.8, 139.9, 138.5, 131.4, 127.1, 125.9, 125.2, 125.1, 124.9, 76.2, 73.2, 66.0, 34.7, 34.0, 33.3, 31.5, 25.9, 25.8, 25.6, 17.8, 17.3, 15.4.

4,4''-di-tert-butyl-5'-(((6-fluoro-2,6-dimethylheptyl)oxy)methyl)-1,1':3',1''-terphenyl (28)

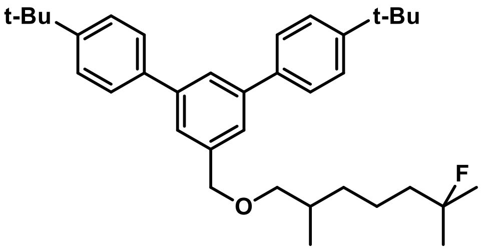

$C_{36}H_{49}FO$
516.38 g/mol

Ether **27** (100 mg, 0.20 mmol, 1.0 eq.) was reacted with iron (III) oxalate hexahydrate (389 mg, 0.80 mmol, 4.0 eq.), Selectfluor (300 mg, 0.80 mmol, 4.0 eq.), and $NaBH_4$ (101 mg, 2.57 mmol, 12.8 eq.) according to modified general procedure B for 30 min at 0 °C in a mixture of THF/MeCN/water (4:2:3, 16 mL). Column chromatography [SiO_2, pentane/Et_2O = 50/1) gave fluoride **28** (26.1 mg, 50 µmol, 25%) as a colorless oil.

TLC: R_f = 0.34 (pentane/Et_2O = 50/1) [UV, $KMnO_4$].

^{1}H NMR (500 MHz, $CDCl_3$): δ[ppm] = 7.71 (t, ^4J = 1.7 Hz, 1H), 7.62 – 7.57 (m, 4H), 7.52 (d, ^4J = 1.7 Hz, 2H), 7.50 – 7.46 (m, 4H), 4.61 (s, 2H), 3.41 – 3.29 (m, 2H), 1.86 – 1.76 (m, 1H), 1.64 – 1.55 (m, 2H), 1.52 – 1.41 (m, 2H), 1.37 (s, 18H), 1.31 (d, ^3J = 21.5 Hz, 6H), 1.17 – 1.06 (m, 1H), 0.96 (d, ^3J = 6.7 Hz, 3H).

^{13}C NMR (126 MHz, $CDCl_3$): δ[ppm] = 150.6, 141.8, 139.8, 138.4, 127.0, 125.9, 125.2, 125.1, 76.2, 73.3, 34.7, 34.2, 33.7, 31.5, 26.9, 26.6, 21.5, 17.3.

^{19}F NMR (471 MHz, $CDCl_3$): δ[ppm]: – 137.7.

4. Elimination of HF: determination of yields

General procedure (small scale, NMR analysis): Substrate stock solution (100 µL, 16.7 µmol, 10 eq.), *C*-Undecylcalix[4]resorcinarene stock solution (100 µL, 10 µmol, 6.0 eq), hexafluorobenzene stock solution (20 µL, 1.7 µmol, 1.0 eq.), and 280 µL of filtered $CDCl_3$ were added to a GC vial (1 mL size). Immediately upon mixing a small aliquot (20 µL) was taken from the reaction mixture and diluted with 0.5 mL of acetone-d_6 and subjected to ^{1}H and ^{19}F NMR. The GC vial was then kept at 40 °C (±1 °C) using a thermostated aluminum heating block. The progress of the reaction was monitored *via* ^{1}H and ^{19}F NMR. For this purpose, the GC vials containing the reaction mixture were removed from the heating block and upon cooling to r.t. a small aliquot (20 µL) was taken, diluted with 0.5 mL of acetone-d_6 and subjected to NMR spectroscopy. The influence of the temperature drop during removal of aliquots was neglected.

Reactions in presence of capsule **I** were conducted using stock solutions of *C*-Undecylcalix[4]resorcinarene and hexafluorobenzene in $CDCl_3$. Prior to usage, $CDCl_3$ was filtered over a basic Al_2O_3 plug (5-6 mL over 2-3 g Al_2O_3) to remove trace amounts of HCl/DCl, potentially generated by photodegradation of $CDCl_3$. The stock solution for *C*-Undecylcalix[4]resorcinarene was prepared by weighing in monomer (111 mg, 0.10 mmol) in a 1 mL volumetric flask, which was then filled to less than full capacity with filtered $CDCl_3$ and homogenized in an ultrasonic water bath. In case of incomplete dissolution, the flask was heated to approx. 40 °C with a heat gun under agitation. After complete dissolution, the flask was then filled to the calibration mark with filtered $CDCl_3$ and again agitated to give a clear yellow solution.

The stock solution for the internal ^{19}F NMR standard was prepared by weighing hexafluorobenzene (3.11 mg, 0.017 mmol) into a GC vial and filling up with 200 µL of filtered $CDCl_3$. The small contribution of the internal standard to the total volume of the stock solution was neglected. Substrates were added as stock solutions in filtered $CDCl_3$ (100 µL, 167 mmol/L, 16.7 µmol). In all cases the amount of added $CDCl_3$ was adjusted to maintain an overall volume of 0.5 mL.

For NMR analysis, the yields were calculated by employing the following equations (2.1 – 2.3).

$$n(sm)_0 = \frac{(I_{sm})_0}{(I_{sm})_{0,exp}} = x \tag{2.1}$$

$$n(p)_n = \frac{(I_p)_n}{(I_p)_{exp}} = z \tag{2.2}$$

$$yield(p) = \left(\frac{z}{x}\right) \cdot 100\% \tag{2.3}$$

$n(sm)_0$ = amount of starting material in the initial measurement; $n(p)_n$ = amount of product in the n-th measurement (full conversion/equilibrium reached); $(I_{sm})_0$ = integral of a characteristic starting material resonance in the initial measurement, after normalizing an integral value of a *C*-Undecylcalix[4]resorcinarene resonance (bridging CH group = 24H, δ = 4.31 ppm); $(I_{sm})_{0,exp}$ = expected integral of the corresponding resonance assuming 10.0 eq. of starting material; $(I_p)_n$ = integral of a characteristic product resonance (usually olefinic proton resonance) in the n-th resonance measurement, after normalizing an integral value of C-Undecylcalix[4]resorcinarene resonance; $(I_p)_{n,exp}$ = expected integral of the corresponding resonance assuming complete and selective conversion of 10.0 eq. substrate.

If possible, multiple resonances were taken into account. The calculated mean values were then used for the equations. Results are summarized in table 1.

SI-Table 1. Experimental results in presence of hexamer **I**.

#	Substrate	Products	Background
1	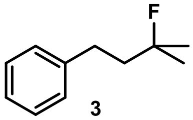 **3**	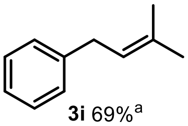 **3i** 69%[a] 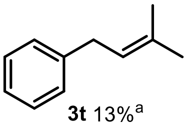 **3t** 13%[a]	0[b]<br>0[c]
2	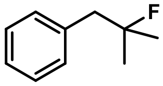 **4**	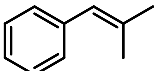 **4i** 45%[a] 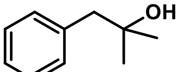 **4a** 35%[a] 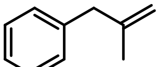 **4t** 16%[a]	0[b]<br>0[c]
3	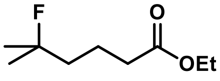 **5**	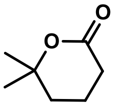 **5l** 60%[a] 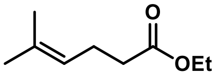 **5i** 29%[a] 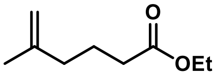 **5t** 3%[a]	0[b]<br>0[c]
4	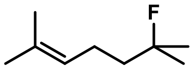 **6**	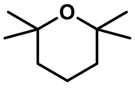 **6e** 81%[a] 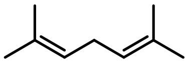 **6i** 11%[a]	0[b]<br>0[c]
5	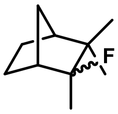 **7**	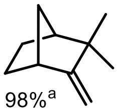 98%[a]	98%[b] (20 h)<br>0[c]
6	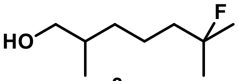 **8**	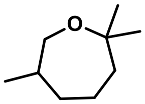 **8e** 72%[a]	**8e** 71%[b],<br>0[c]
7	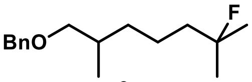 **9**	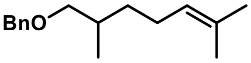 **9i** 65%[a] 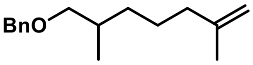 **9t** 6%[a]	**9i** 5%[b]<br>0[c]
8	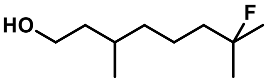 **10**	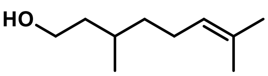 **10i** 60%[a] 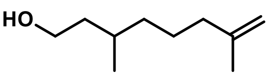 **10t** 6%[a] 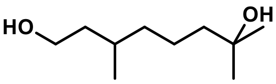 **10a** 16%[a]	**10i** 52%[b]<br>**10t** 6%[b]<br>**10a** 13%[a]<br>0[c]

all reactions run in $CDCl_3$ (filtered over basic Al_2O_3 prior to use) at 40 °C, [a]determined *via* ^{1}H NMR, [b]addition of TBAB (1.5 eq. rel. to capsule **I**), reaction run for 7 d at 40 °C [c]addition of 10 mol% HOAc, no hexamer present, 7 d, 40 °C.

5. Mechanistic investigations

5.1 Reaction kinetics

Kinetic measurements of the reaction of substrate **3** inside hexamer **I** were performed to gain further insight into the reaction mechanism. To this end, a standard NMR tube (ø = 5 mm) was filled with a solution of **3** in $CDCl_3$ (filtered over basic Al_2O_3 to remove acid traces) containing 10 mol% of **I** and 10 mol% of C_6F_6 as internal standard for ^{19}F-NMR spectroscopy. The tube was then inserted into the magnet of a *Bruker* AV 600 NMR machine and kept at the indicated temperature using the integrated thermostat of the NMR magnet. The sample was kept inside the magnet for the duration of the reaction. The reaction progress was monitored at precise intervals using ^{19}F-NMR spectroscopy, the results are plotted in SI-Figure 1.

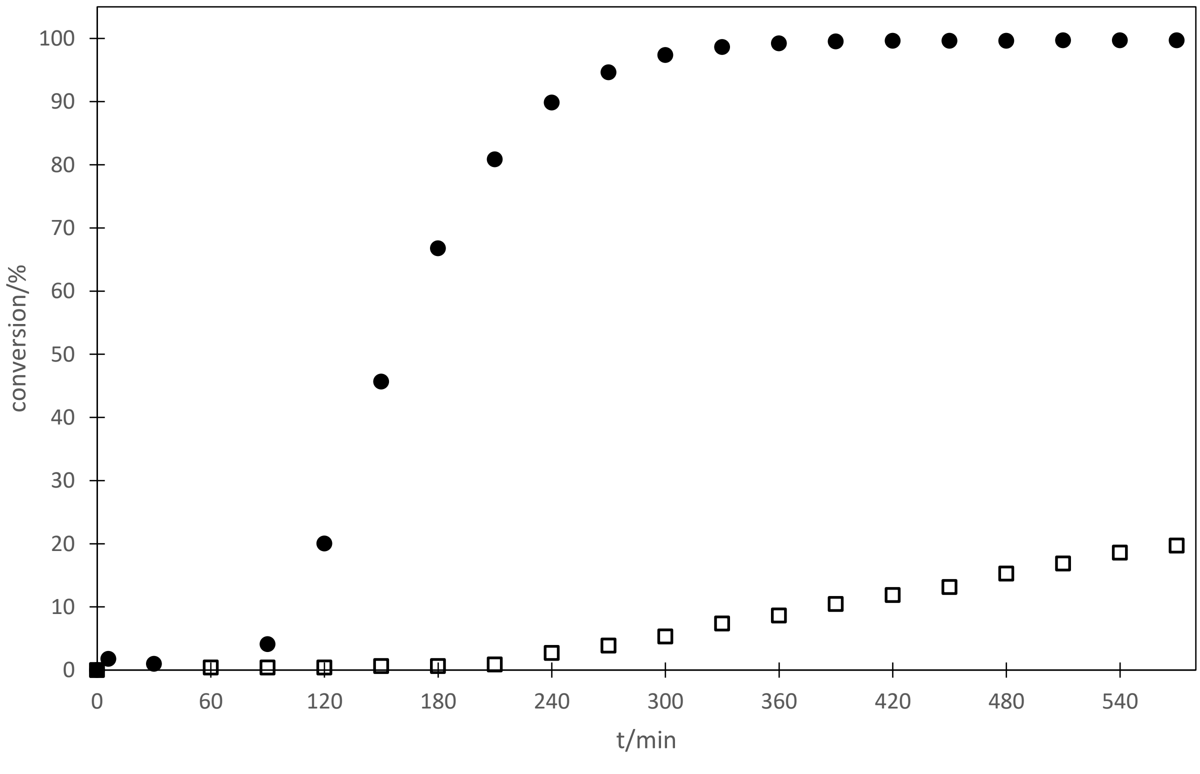

SI-Figure 1. Reaction progress for the elimination of HF from **3** in presence of 10 mol% capsule **I**; 30 °C (□); 40 °C (●).

A sigmoidal progression of the reaction can be observed for the reaction at 40 °C. At 30 °C the reaction proceeds in a linear fashion.

5.2 Experiments in plastic vials

A plastic *Eppendorf* vial (1 mL) was filled with a solution of substrate **3** (in filtered $CDCl_3$) containing 10 mol% of each HF (added as stock solution in MeOH-d_4, prepared by diluting aq. HF (48 – 52%) with MeOH-d_4; total volume added: 1.0 µL) and C_6F_6 (internal standard for ^{19}F-NMR spectroscopy). The vial was kept at 40 °C in a thermostated aluminum heating block. In regular intervals, small aliquots (20 µL) were taken from the reaction mixture, diluted to 500 µL with acetone-d_6 and monitored via ^{19}F-NMR. Another reaction was set up in parallel with additonal 10 mol% of hexamer **I**. The reaction profiles are displayed in SI-Figure 2.

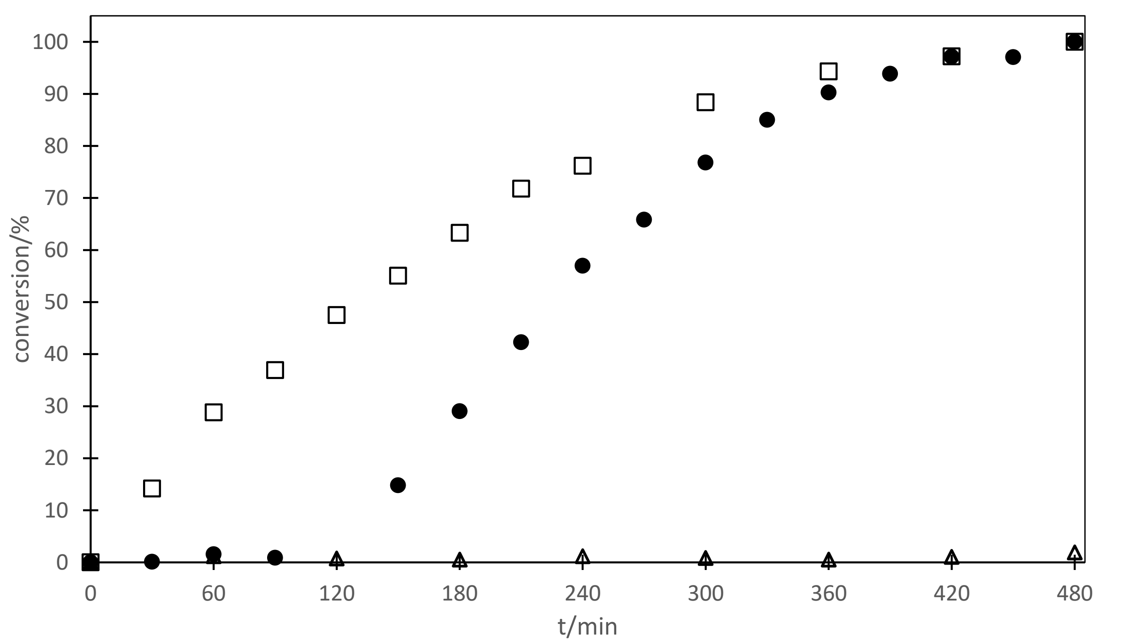

SI-Figure 2. Reaction progress for the reaction of **3** in presence of 10 mol% **I** at 40 °C (●), at 40 °C with 10 mol% HF in absence of hexamer **I** (∆), at 40 °C with 10 mol% **I** and 10 mol% HF (□).

Contrary to our expectation, these conditions did not reduce the induction phase that was observed at 40 °C, and the reaction in absence of **I** never advanced to the logarithmic linear phase. Reaction progress remained sluggish over the course of the whole observation period, reaching 2% conversion after 8 hours.

When 10 mol% HF (stock solution in MeOH-d_4) were added to a GC vial containing **3** and 10 mol% **I**, an immediate start of the reaction was observed. This might indicate a beneficial synergy between HF and capsule **I**.

5.3 KIE experiments

A competition experiment between **3** and its deuterated analogue **3d** was conducted to determine a kinetic isotope effect. To this end, as was described above, a 1:1 mixture of **3** and **3d** with 10 mol% **I** and 1 eq. C_6F_6 was continually measured in a AV 600 *Bruker* NMR machine at 40 °C and the reaction progress monitored via ^{19}F-NMR spectroscopy (see SI-Figure 3). The results are summarized in table 3.

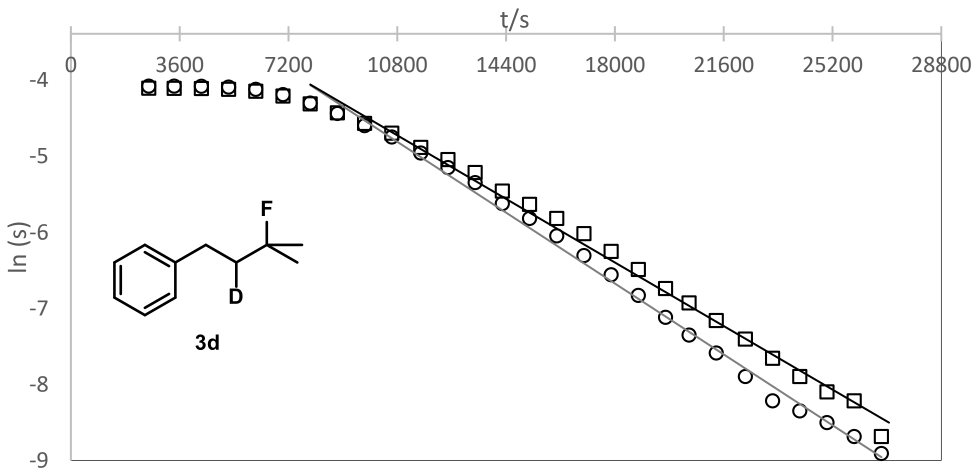

SI-Figure 3. First-order plot for the parallel conversion of **3** (○) and **3d** (□) in presence of 10 mol% I at 40 °C.

Table 2. Experimentally determined values for the reactions rates of **3** and **3d**.

substrate	rate [1/s]	R^2
**3**	$2.54 \cdot 10^{-4} \pm 5.95 \cdot 10^{-6}$	0.99
**3d**	$2.19 \cdot 10^{-4} \pm 5.09 \cdot 10^{-6}$	0.99

Calculating from these values, a KIE of 1.16 ± 0.03 could be determined. This is in agreement with a secondary KIE arising from stabilization of the tertiary cation *via* hyperconjugation.

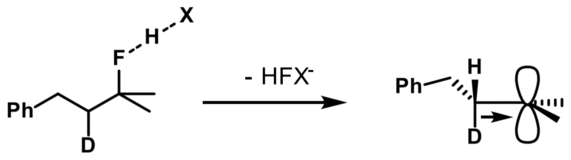

SI-Scheme 6. Rate-determining step of the reaction of **3** inside capsule **I**; X = hexameric assembly or HF.

5.4 Substrate uptake

Compelling evidence for the uptake of fluorinated species was obtained via ^{1}H NMR (see SI-Fig. 4 and 5). High-field shifted peaks at δ = 0.2 ppm to δ = – 0.5 ppm appeared immediately after addition of 3 eq. of substrate **8** and 10 eq. of **3** resp. to a solution of capsule **I** (3.33 mM in filtered $CDCl_3$).

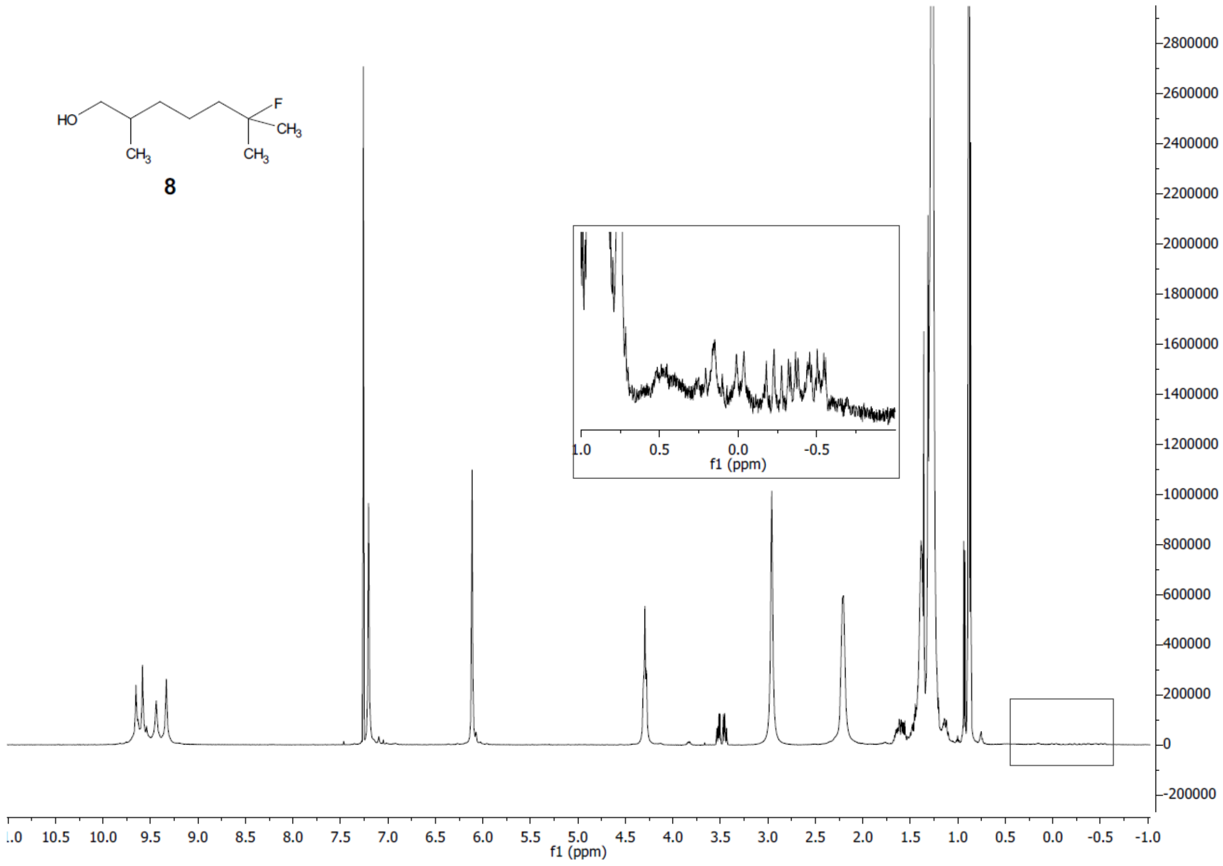

SI-Figure 4. ^{1}H NMR spectrum of capsule **I** in presence of alcohol **8** (3 eq. relative to **I**).

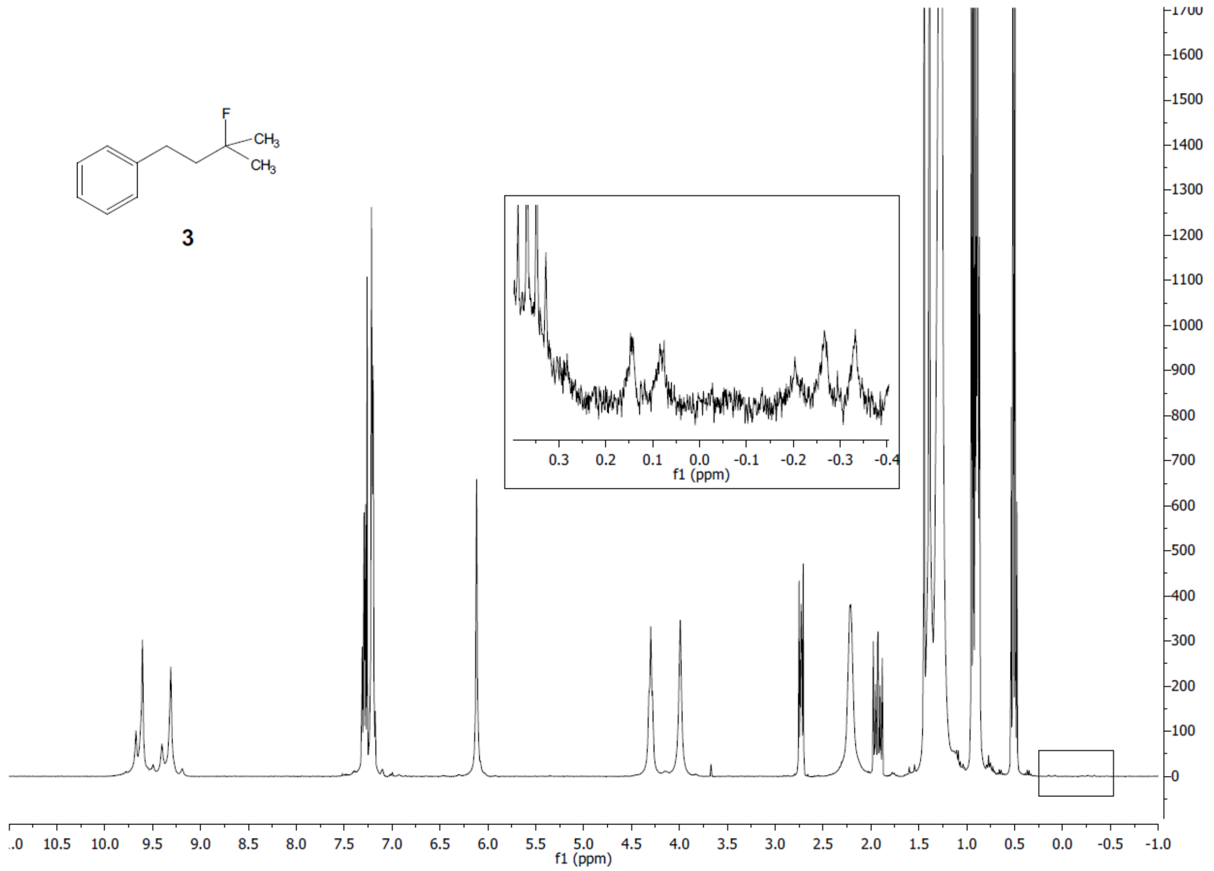

SI-Figure 5.^{1}H NMR spectrum of capsule **I** in presence of fluoride **3** (10 eq. relative to **I**).

5.5 Reaction of large substrate **28**

A control reaction was performed with large ether **28** to test whether the elimination of HF could be observed from a substrate that is too large for encapsulation. After 96 h at 40 °C no conversion was observed. This strongly implies that substrates need to be encapsulated in order to undergo rapid elimination to the respective products.

However, when a competition experiment was conducted with benzyl ether **9** and the large substrate **28** in presence of 10 mol% capsule **I**, both substrates were fully converted within 4 h (see SI-Figure 7). As mentioned above, ether **28** is stable in presence of hexamer **I** over the course of multiple days. This implies that the conversion of the smaller benzyl ether **9** releases HF, which then catalyzes the conversion of substrate **28**, outside of the capsule.

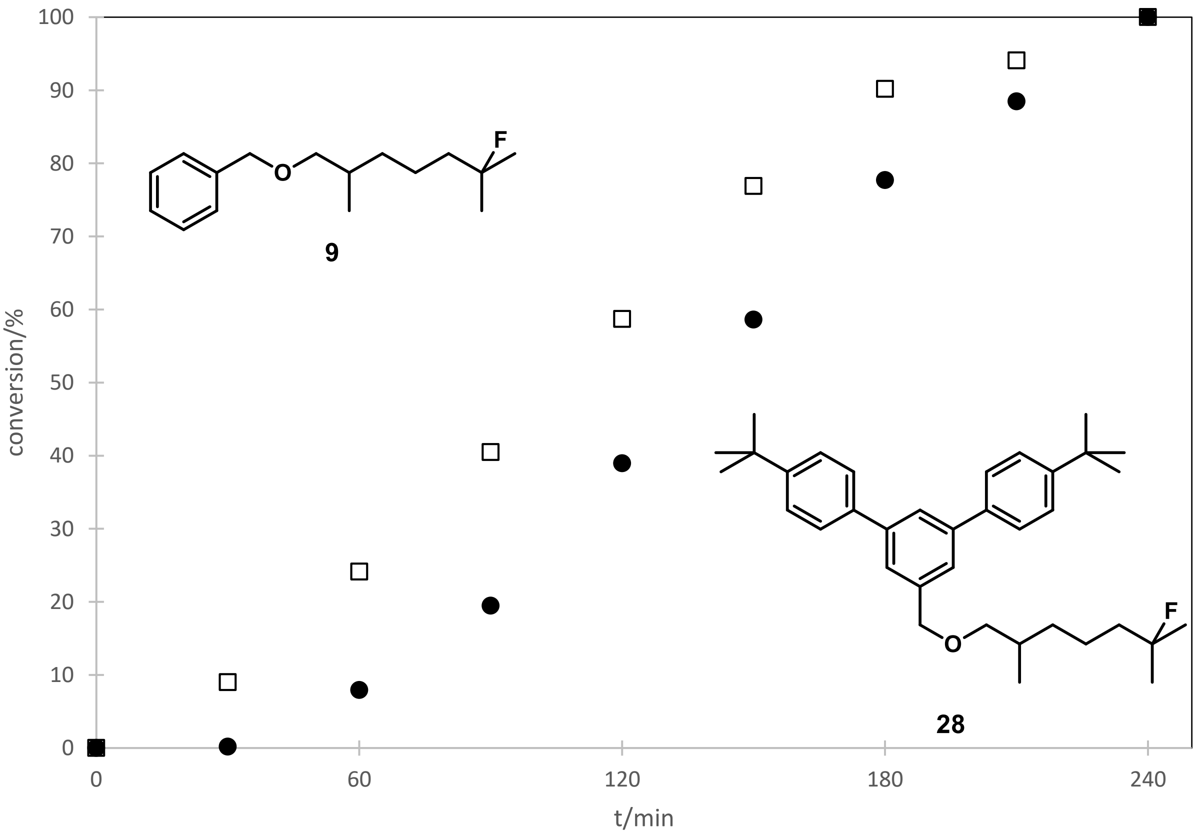

SI-Figure 6. Reaction of fluorides **9** (□) and **28** (●) at 40 °C with 10 mol% capsule **I**, reaction performed in a GC vial, aliquots were taken from the reaction mixture every 30 min.

5.6 Influence of reaction vessel

To assess the influence of different reaction vessels and monitoring methods, the elimination reaction of substrate **3** was performed in an NMR tube (see kinetic experiments above) and in a GC vial, from which aliquots were taken, diluted with acetone-d_6 and then subjected to ^{19}F NMR spectroscopy.

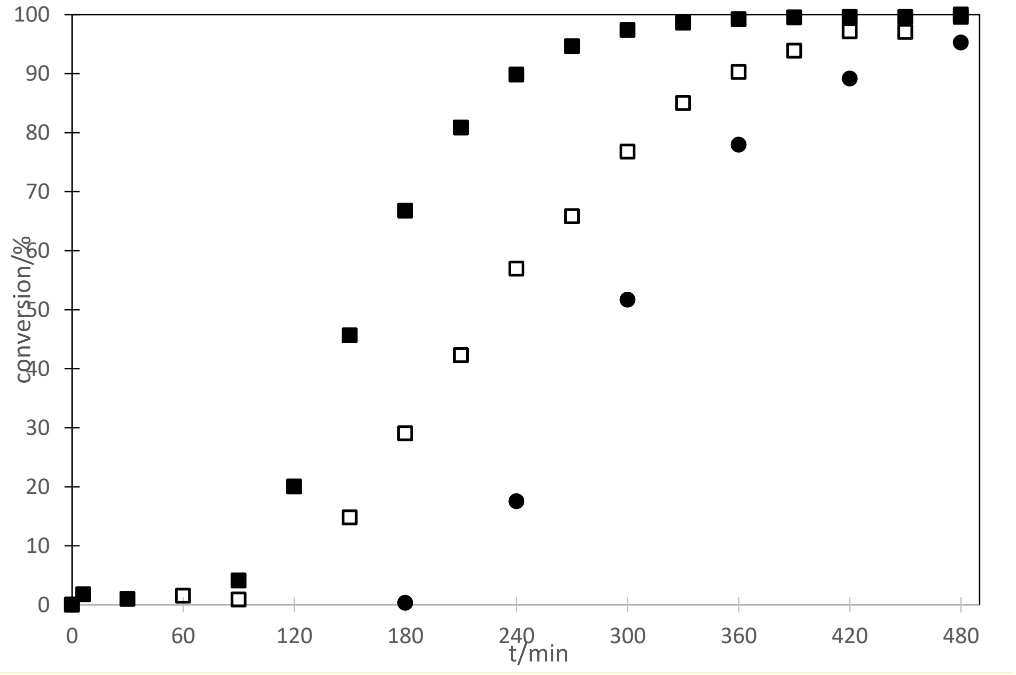

SI-Figure 7. Plot of conversion against time of substrate **3** at 40 °C, reaction performed in NMR tube (■), in GC vial (□), GC vial equipped with stir bar, 180 rpm (●).

Comparing the two reactions (see SI-Figure 7) it immediately becomes apparent the vessel has a non-negligible effect on reaction progress. The reaction performed in a NMR tube shows a steeper conversion rate and reached full conversion earlier than the reaction run in a GC vial. This could be explained by the fact that the reaction in the NMR tube is constantly kept inside the magnet whereas the GC vial is frequently taken out of the heating block, cooled to r.t. and agitated while aliquots are taken. To further assess the influence of agitation upon the reaction progress the reaction was performed in a GC vial and constantly stirred at 180 rpm. A longer induction period was observed, after which the conversion showed a very similar profile to the non-stirred reaction.

Considering these findings reaction kinetics not performed in a NMR magnet are not suited to determine reaction rates and should only be consulted for qualitative statements.

Furthermore, a different reaction profile was obtained when the reaction of **3** was monitored in a NMR tube that had previously been subjected to the reaction, i.e. the tube walls had already been etched by released HF. A significantly slower reaction progress was observed, likely caused by the increased surface area of the etched tube walls, leading to more efficient quenching of released HF.

6. Control experiments

6.1 Blocked cavity

To verify that the reactions proceed inside the cavity of the hexamer, the conversion and yield of all substrates were determined in the presence of a high affinity guest that would act as a competitive inhibitor by blocking the cavity. On the basis of previous reports,[18] tetrabutylammonium bromide (Bu_4NBr) was chosen as a suitable inhibitor. Additionally, it has been demonstrated that the acidity of the hexamer is increased upon encapsulation of Bu_4NBr.[18] This enhances the quality of the control experiment, because the background reaction outside the cavity may even be favored when the inhibitor is encapsulated. For the control reactions, a small excess (1.5 eq. relative to hexamer **I**) of guest **2** was utilized in order to ensure complete blocking of all cavities.

General procedure for the control reaction with inhibitor 2:

Aliquots of substrate stock solution (100 µL, 16.7 µmol, 10 eq.), hexafluorobenzene stock solution (20 µL, 1.7 µmol, 1.0 eq.), Bu_4NBr stock solution (100 µL, 2.51 µmol, 1.5 eq.), and *C*-Undecylcalix[4]resorcinarene stock solution (100 µL, 10.0 µmol, 6 eq.) – prepared as described above – were added to a 1 mL GC vial. After addition of 180 µL $CDCl_3$ a small aliquot (20 µL) was taken of the reaction mixture, diluted with 0.5 mL acetone-d_6, and subjected to ^1H and ^{19}F NMR spectroscopy. The GC vial was then kept at 40 °C (±1 °C) using a thermostated aluminum heating block. A new measurement was taken after theindicated time required for optimum yield in the absence of inhibitor. In case no reaction could be observed, a final measurement was taken after seven days. The conversion and yield were then determined by employing the equations above (2.1 – 2.3). To ensure identical reaction conditions, the same stock solution of *C*-Undecylcalix[4]resorcinarene (**1**) in filtered $CDCl_3$ was used as for the yield determination in the absence of inhibitor.

6.2 Blocked cavity after induction period

Kinetic measurements of the reaction of **3** in presence of hexamer **I** showed the existence of a slow induction phase and a linear logarithmic phase. To verify that both phases are characterized by the conversion of the fluoride inside the cavity, a control reaction was conducted. To a reaction of **3** in a GC vial with 10 mol% of **I** at 40 °C, the high-affinity guest TBAB (**2**) was added after 3 hours, which marks the previously observed transition to rapid conversion of the substrate (see SI-Figure 3).

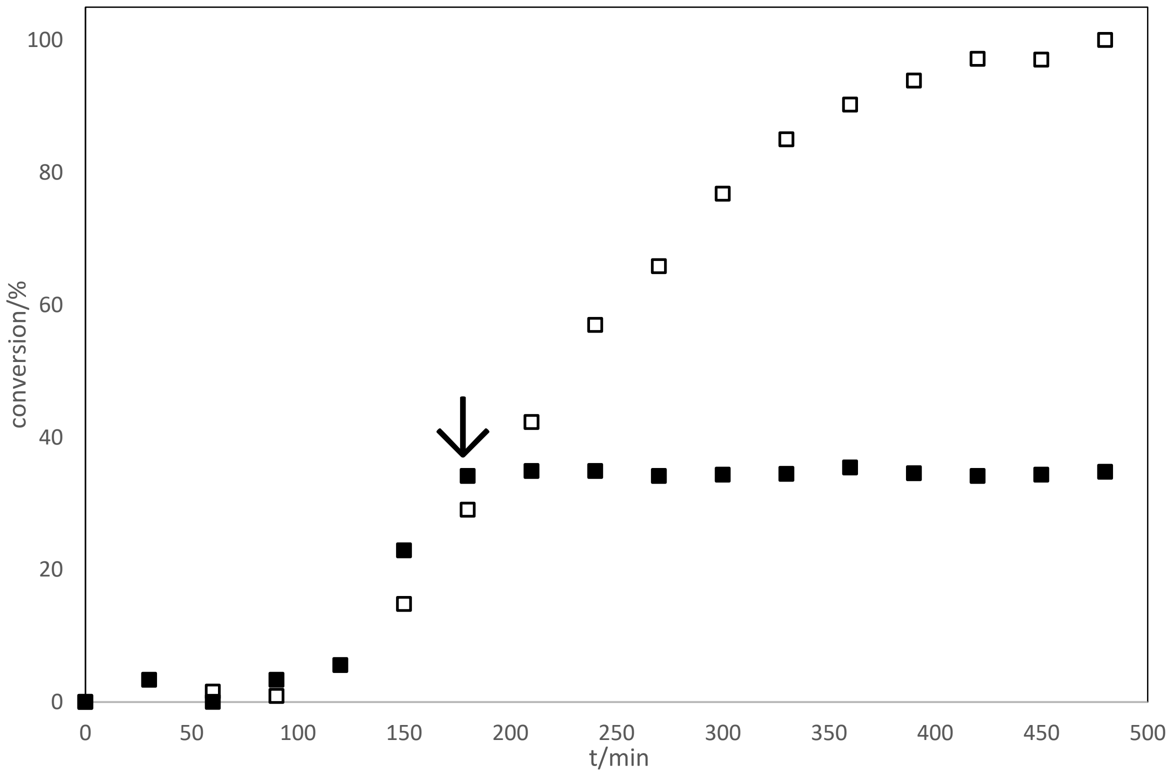

SI-Figure 8. Conversion of **3** inside hexamer **I** at 40 °C (□) and with addition of TBAB (■, arrow marks point of addition of TBAB).

The addition of TBAB led to a complete stop of the reaction at a conversion of <40%. This strongly indicates that the elimination reaction takes place inside the cavity of capsule **I**.

6.3 Without catalyst

Control experiments in the absence of catalyst were performed to exclude a background reaction in filtered $CDCl_3$. The substrate (16.7µmol, 10.0 eq.) was added to an NMR tube and dissolved in $CDCl_3$ (0.50 mL). The sample was subjected to NMR spectroscopy and then kept in a thermostated aluminum heating block at 40 °C (±1 °C). After seven days, the sample was measured again. NMR spectroscopy indicated no reaction in all cases after seven days.

6.4 With catalytic amounts of acetic acid in solution

Aliquots of stock solution of substrate (100 µL, 16.7 µmol, 10.0 eq.), hexafluorobenzene (20 µL, 1.67 µmol, 1.0 eq.) and acetic acid (100 µL, 1.67 µmol, 1.0 eq.) were added to a 1 mL GC vial. After filling up with 280 µL filtered $CDCl_3$ and mixing, small aliquots – as described above – were taken and subjected to NMR spectroscopy. The vial was then kept in a thermostated aluminum heating block at 40 °C (±1 °C). After seven days the samples were measured *via* ^{1}H and ^{19}F NMR, which showed no reaction for all substrates **3** – **10** and **28**.

7. References

[1] I. Elidrisi, S. Negin, P. V. Bhatt, T. Govender, H. G. Kruger, G. W. Gokel, G. E. M. Maguire, *Org. Biomol. Chem.* **2011**, *9*, 4498-4506.

[2] a) T. J. Barker, D. L. Boger, *J. Am. Chem. Soc.* **2012**, *134*, 13588-13591; b) D. F. Taber, R. P. Meagley, D. J. Doren, *J. Org. Chem.* **1996**, *61*, 5723-5728.

[3] W. J. Middleton, *J. Org. Chem.* **1975**, *40*, 574-578.

[4] T. Umemoto, R. P. Singh, Y. Xu, N. Saito, *J. Am. Chem. Soc.* **2010**, *132*, 18199-18205.

[5] M. T. Reetz, S. H. Kyung, M. Hüllmann, *Tetrahedron* **1986**, *42*, 2931-2935.

[6] X. Xie, S. S. Stahl, *J. Am. Chem. Soc.* **2015**, *137*, 3767-3770.

[7] L. Catti, K. Tiefenbacher, *Chem. Commun.* **2015**, *51*, 892-894.

[8] P. R. Krishna, K. Anitha, G. Raju, *Tetrahedron* **2013**, *69*, 1649-1657.

[9] P. A. Champagne, J. Pomarole, M.-È. Thérien, Y. Benhassine, S. Beaulieu, C. Y. Legault, J.-F. Paquin, *Org. Lett.* **2013**, *15*, 2210-2213.

[10] A. Riswoko, Y. Aoki, T. Hirose, H. Nohira, *Enantiomer* **2002**, *7*, 33-39.

[11] Frans J. Jansen, J. Lugtenburg, *Eur. J. Org. Chem.* **2000**, *2000*, 829-836.

[12] A. Martin-Mingot, G. Compain, F. Liu, M.-P. Jouannetaud, C. Bachmann, G. Frapper, S. Thibaudeau, *J. Fluor. Chem.* **2012**, *134*, 56-62.

[13] G. S. Lal, G. P. Pez, R. J. Pesaresi, F. M. Prozonic, H. Cheng, *J. Org. Chem.* **1999**, *64*, 7048-7054.

[14] S. Rozen, D. Zamir, *J. Org. Chem.* **1991**, *56*, 4695-4700.

[15] K. G. Kulkarni, B. Miokovic, M. Sauder, G. K. Murphy, *Org. Biomol. Chem.* **2016**, *14*, 9907-9911.

[16] P. A. Champagne, Y. Benhassine, J. Desroches, J. F. Paquin, *Angew. Chem.* **2014**, *126*, 14055-14059.

[17] J. R. Sommer, A. H. Shelton, A. Parthasarathy, I. Ghiviriga, J. R. Reynolds, K. S. Schanze, *Chem. Mater.* **2011**, *23*, 5296-5304.

[18] Q. Zhang, K. Tiefenbacher, *J. Am. Chem. Soc.* **2013**, *135*, 16213-16219.

[19] M. Kimura, T. Shiba, M. Yamazaki, K. Hanabusa, H. Shirai, N. Kobayashi, *J. Am. Chem. Soc.* **2001**, *123*, 5636-5642.

8. NMR spectra

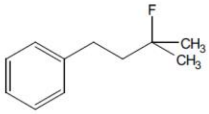

3

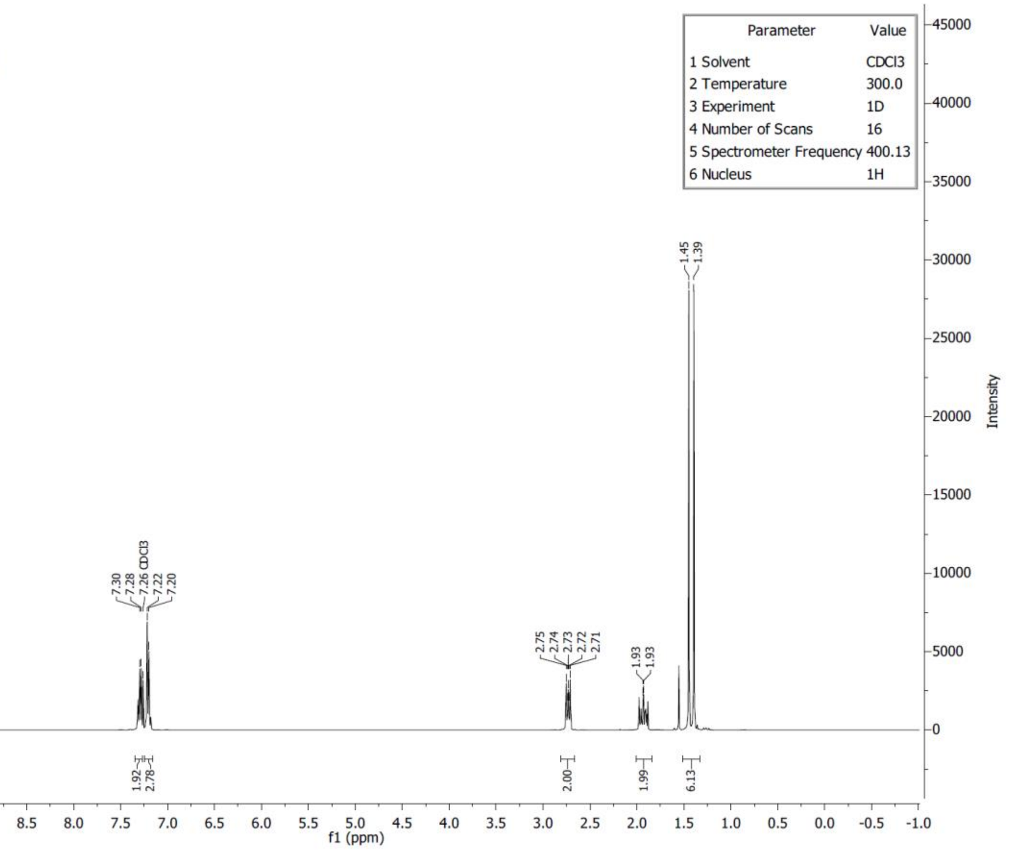

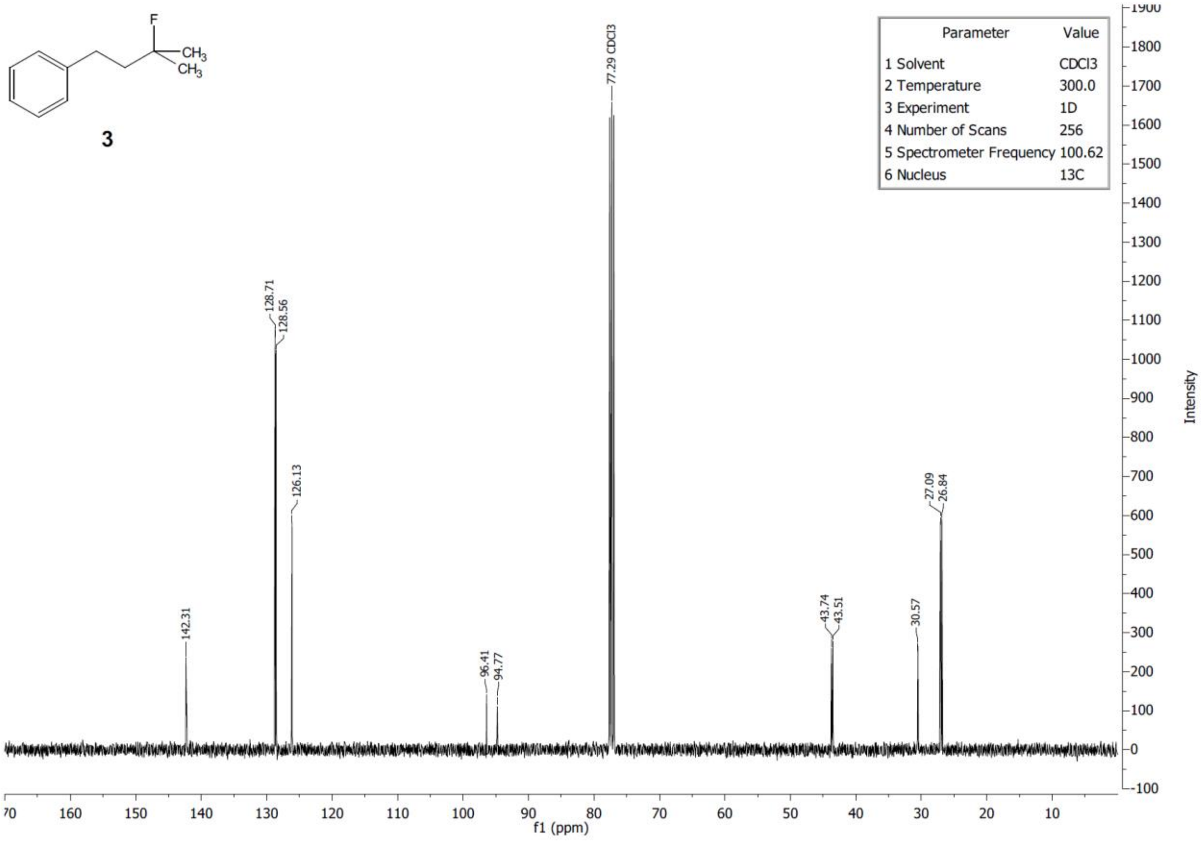
F
CH3
CH3
3
Parameter Value
1 Solvent CDCl3
2 Temperature 300.0
3 Experiment 1D
4 Number of Scans 256
5 Spectrometer Frequency 100.62
6 Nucleus 13C
142.31
128.71
128.56
126.13
96.41
94.77
77.29 CDCl3
43.74
43.51
30.57
27.09
26.84
f1 (ppm)
Intensity

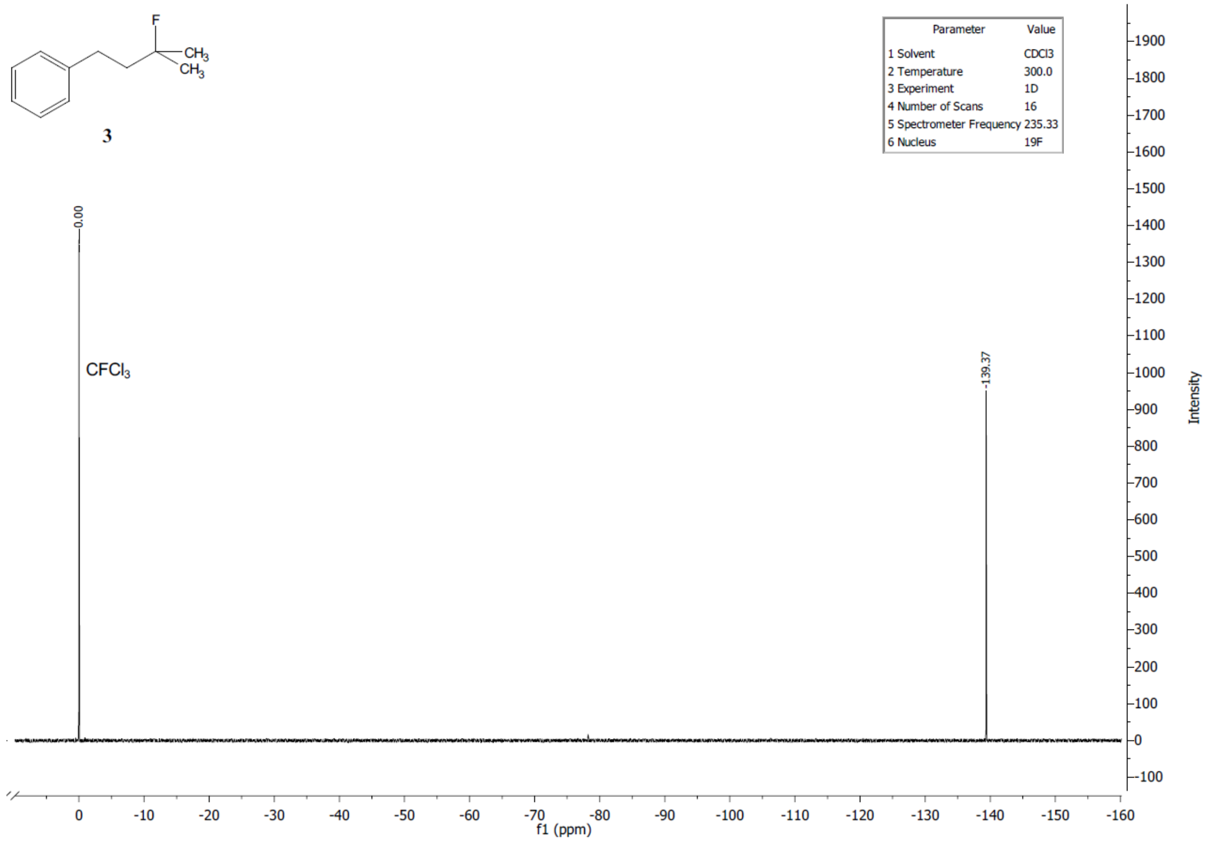
F
CH3
CH3
3
Parameter Value
1 Solvent CDCl3
2 Temperature 300.0
3 Experiment 1D
4 Number of Scans 16
5 Spectrometer Frequency 235.33
6 Nucleus 19F
0.00
CFCl3
-139.37
f1 (ppm)
Intensity

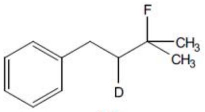
F
CH3
CH3
D

3d

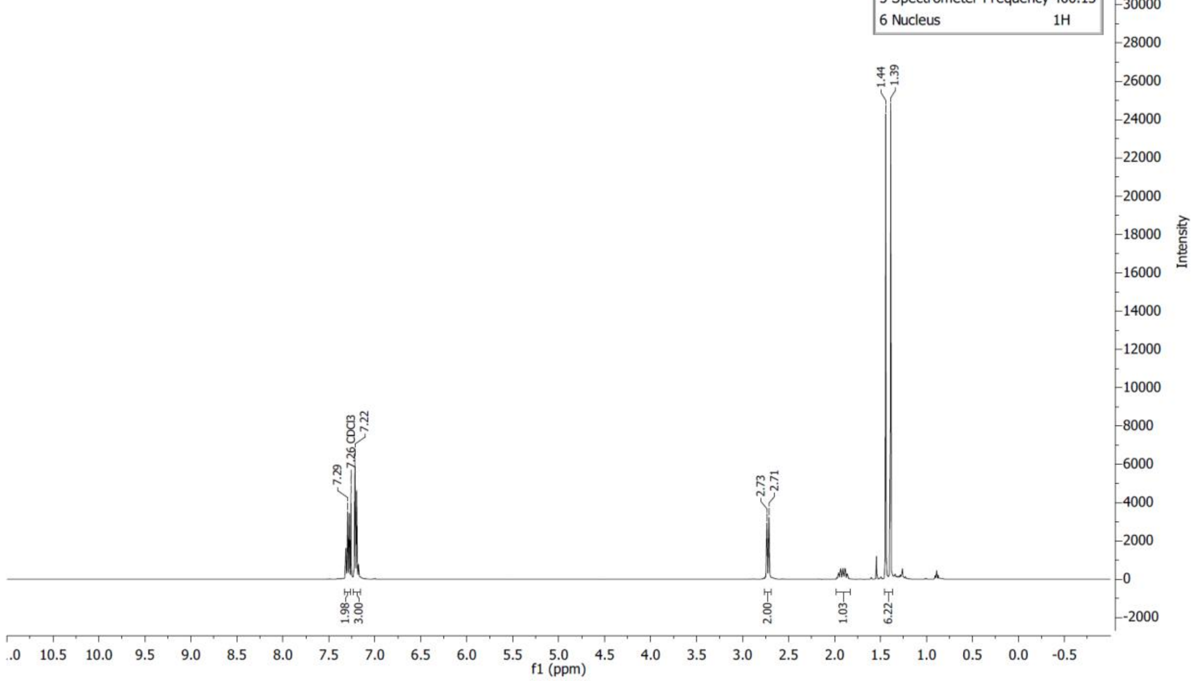
Parameter Value
1 Solvent CDCl3
2 Temperature 300.0
3 Experiment 1D
4 Number of Scans 16
5 Spectrometer Frequency 400.13
6 Nucleus 1H
7.29
7.26 CDCl3
7.22
2.73
2.71
1.44
1.39
1.98
3.00
2.00
1.03
6.22
f1 (ppm)
Intensity

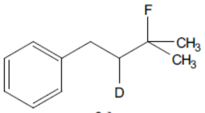
F
CH3
CH3
D

3d

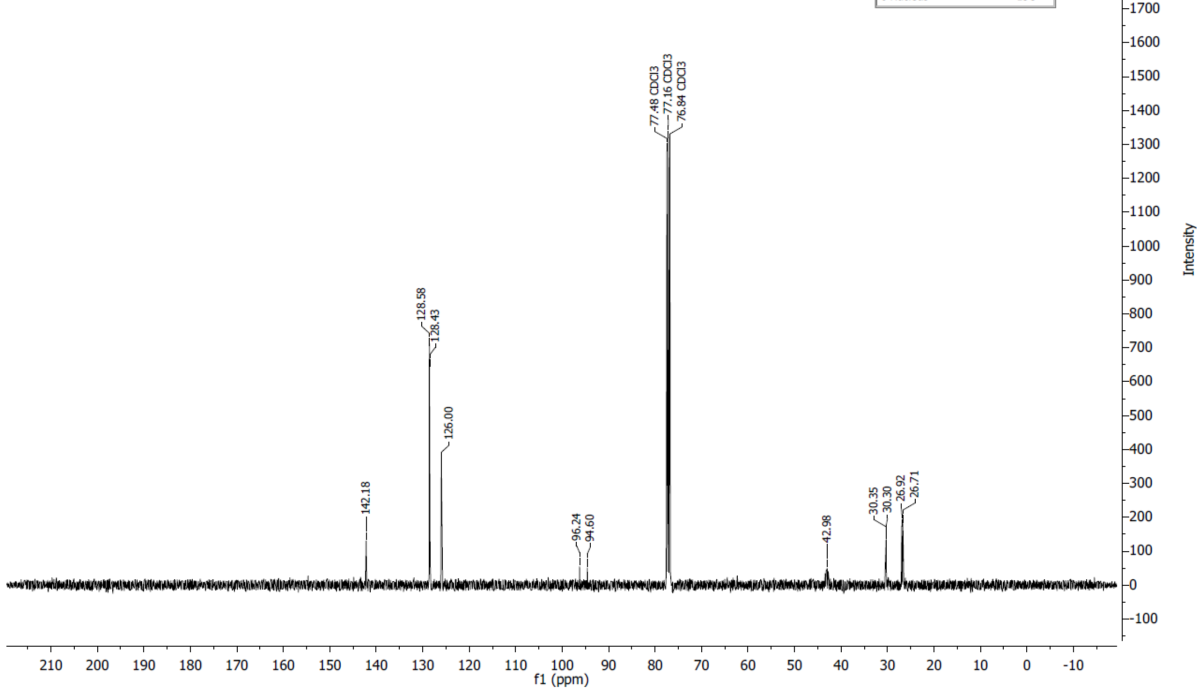
Parameter Value
1 Solvent CDCl3
2 Temperature 300.0
3 Experiment 1D
4 Number of Scans 256
5 Spectrometer Frequency 100.62
6 Nucleus 13C
142.18
128.58
128.43
126.00
96.24
94.60
77.48 CDCl3
77.16 CDCl3
76.84 CDCl3
42.98
30.35
30.30
26.92
26.71
f1 (ppm)
Intensity

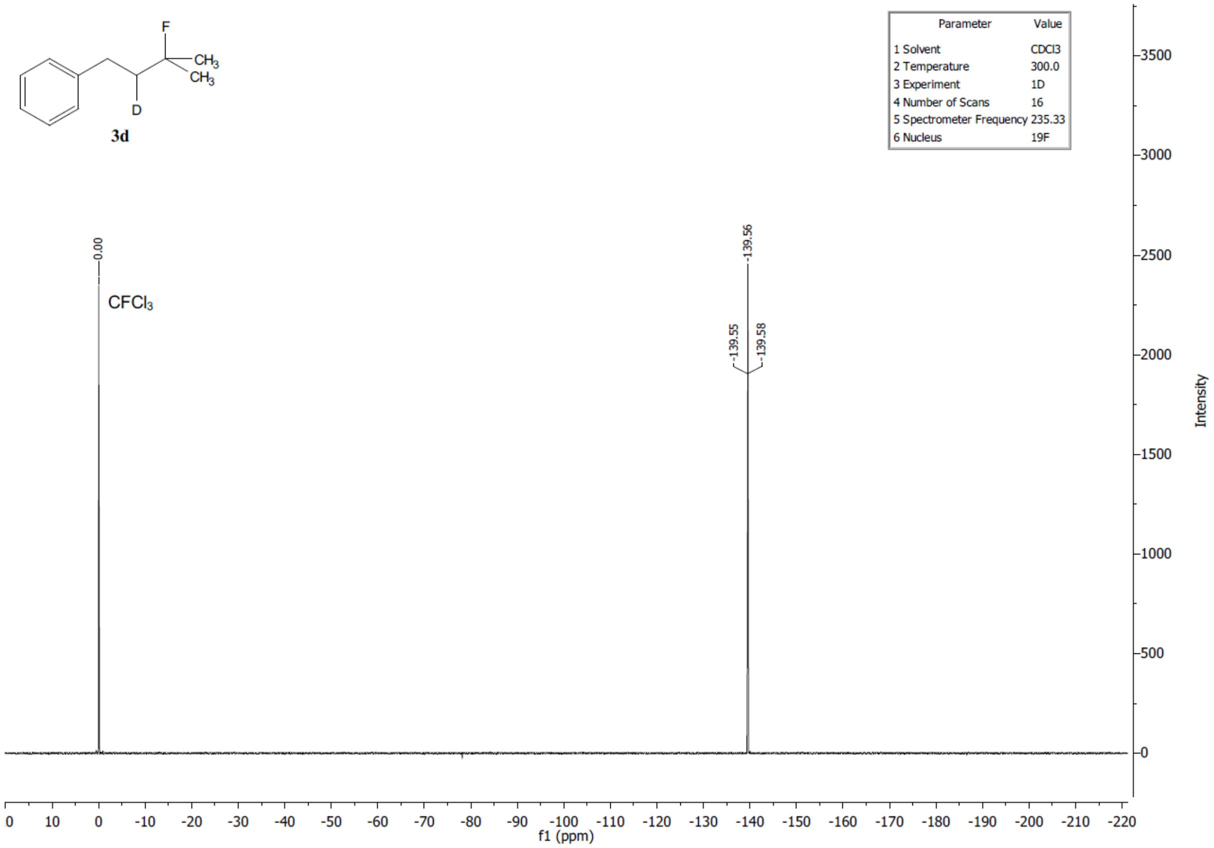
3d
CFCl3
0.00
-139.56
-139.55
-139.58
Parameter Value
1 Solvent CDCl3
2 Temperature 300.0
3 Experiment 1D
4 Number of Scans 16
5 Spectrometer Frequency 235.33
6 Nucleus 19F
f1 (ppm)
Intensity

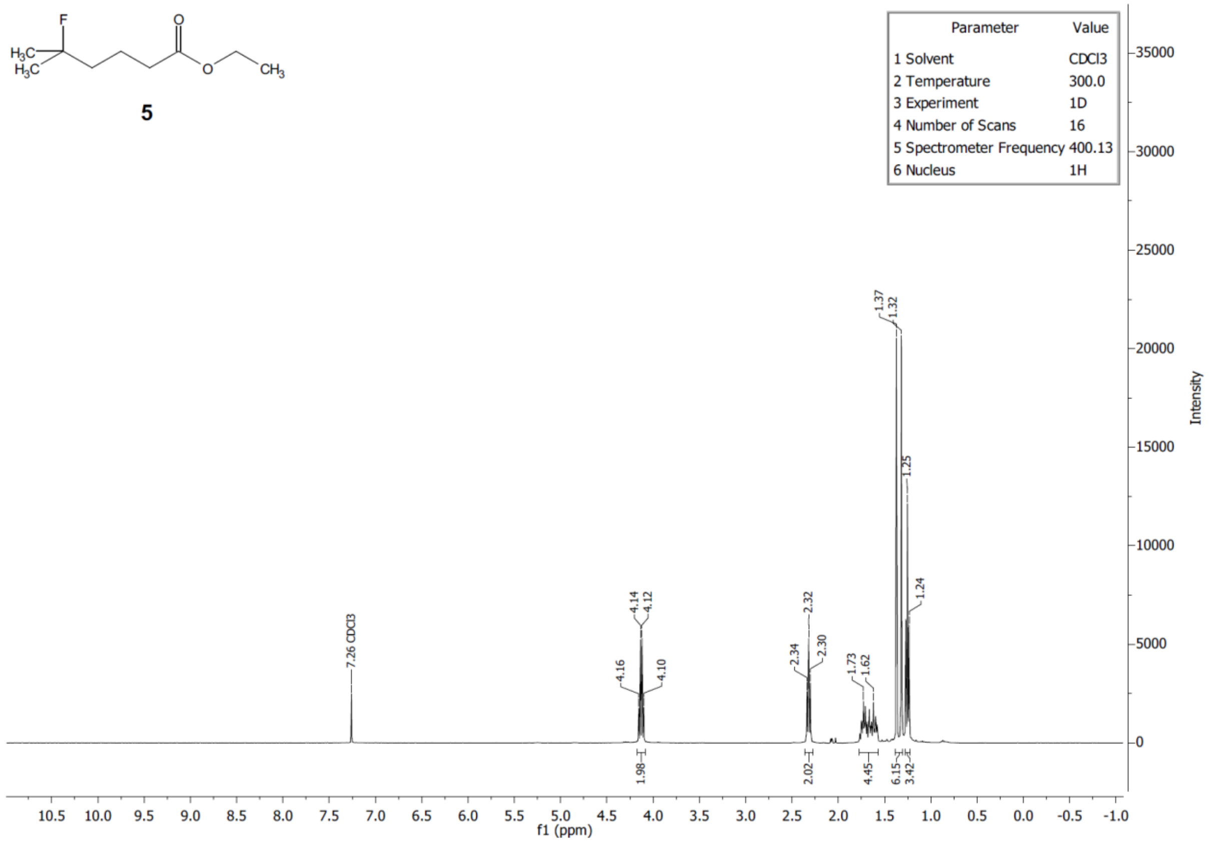
5
Parameter Value
1 Solvent CDCl3
2 Temperature 300.0
3 Experiment 1D
4 Number of Scans 16
5 Spectrometer Frequency 400.13
6 Nucleus 1H
7.26 CDCl3
4.16
4.14
4.12
4.10
2.34
2.32
2.30
1.73
1.62
1.37
1.32
1.25
1.24
1.98
2.02
4.45
6.15
3.42
f1 (ppm)
Intensity

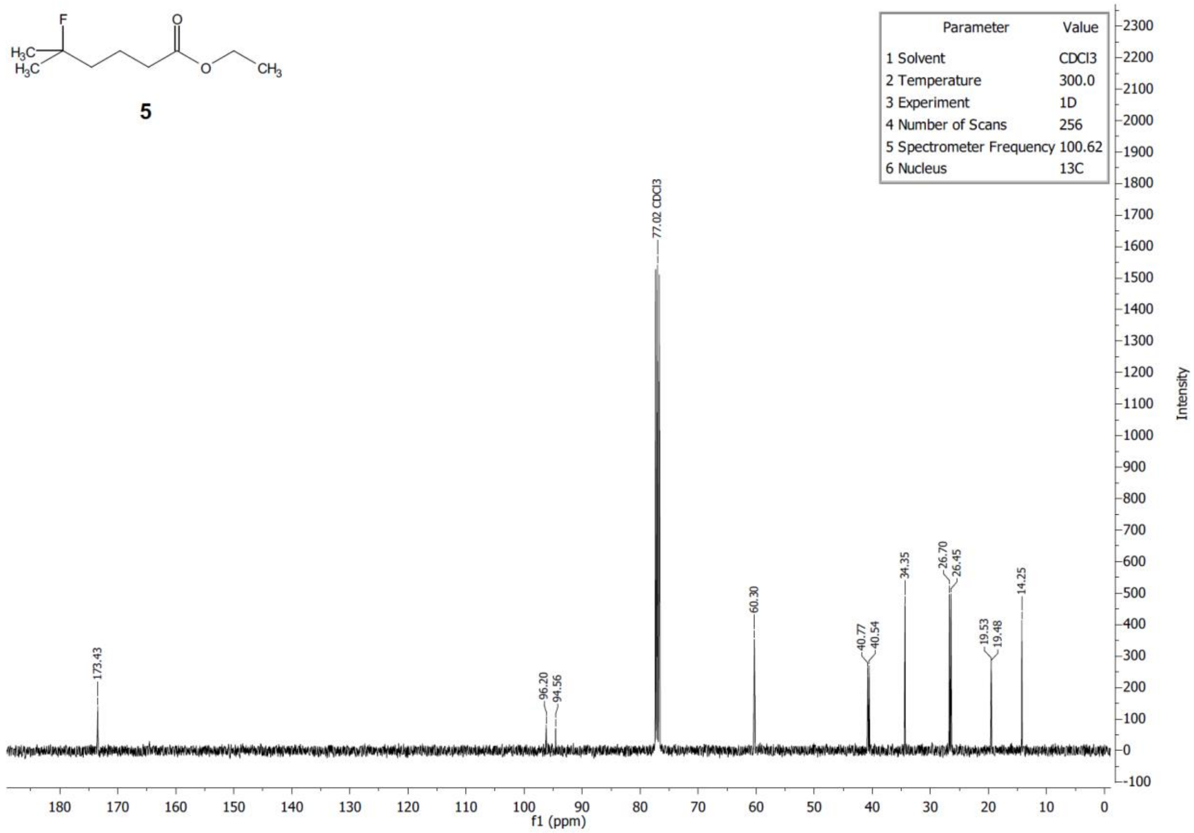
5
Parameter Value
1 Solvent CDCl3
2 Temperature 300.0
3 Experiment 1D
4 Number of Scans 256
5 Spectrometer Frequency 100.62
6 Nucleus 13C
173.43
96.20
94.56
77.02 CDCl3
60.30
40.77
40.54
34.35
26.70
26.45
19.53
19.48
14.25
Intensity
f1 (ppm)

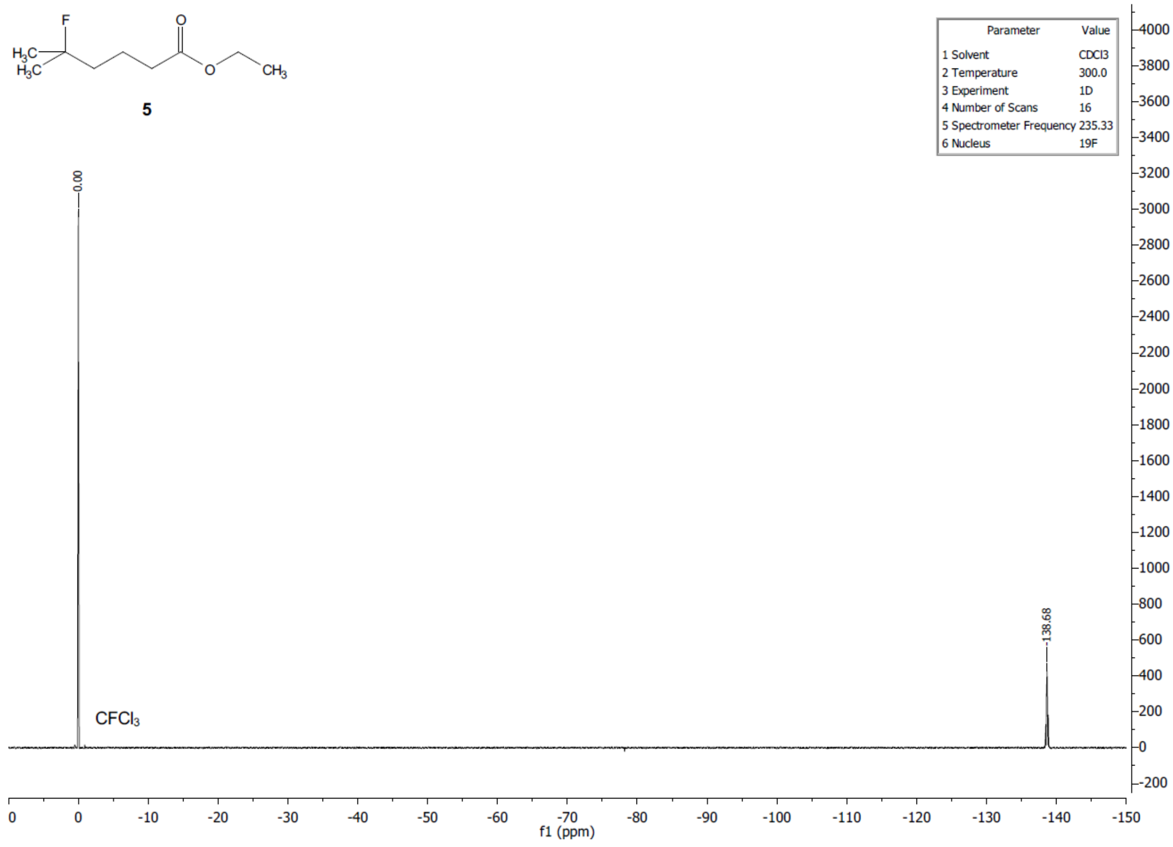
5
Parameter Value
1 Solvent CDCl3
2 Temperature 300.0
3 Experiment 1D
4 Number of Scans 16
5 Spectrometer Frequency 235.33
6 Nucleus 19F
0.00
CFCl3
-138.68
f1 (ppm)

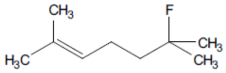

6

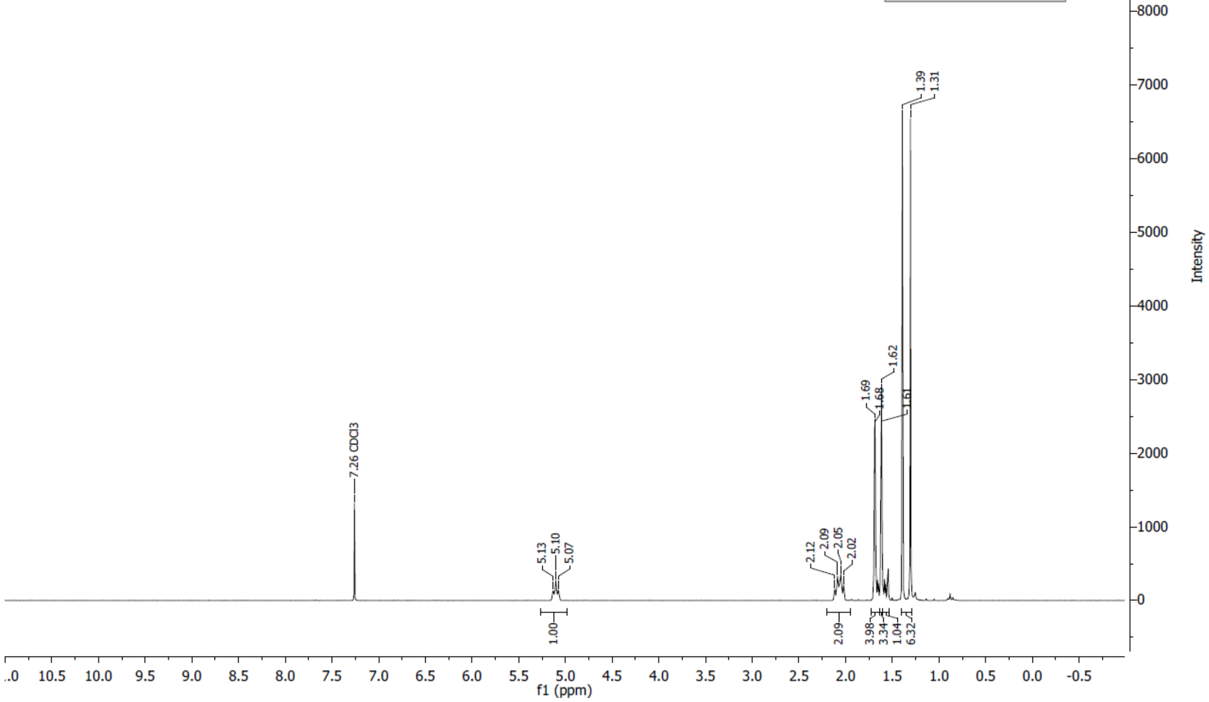
Parameter | Value
1 Solvent CDCl3
2 Temperature 300.0
3 Experiment 1D
4 Number of Scans 8
5 Spectrometer Frequency 250.13
6 Nucleus 1H
7.26 CDCl3
5.13 5.10 5.07
2.12 2.09 2.05 2.02
1.69 1.68 1.62 1.61 1.39 1.31
1.00 2.09 3.98 3.34 1.04 6.32
f1 (ppm)
Intensity

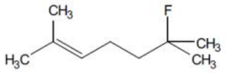

6

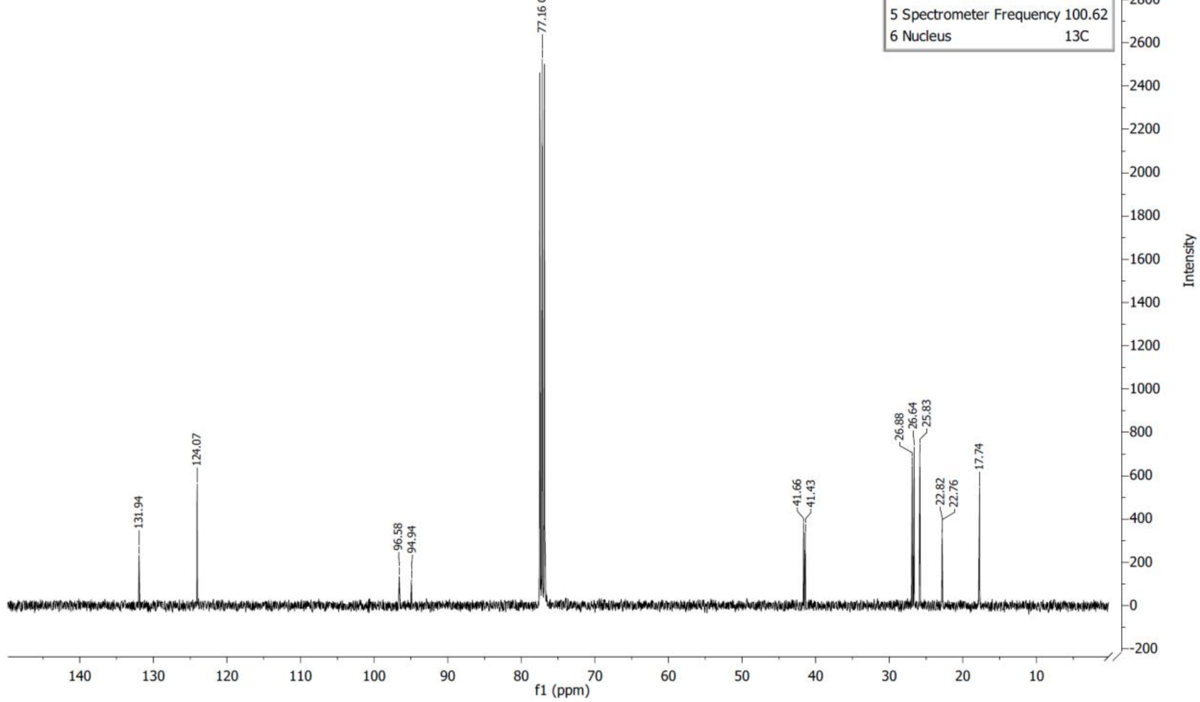
Parameter | Value
1 Solvent CDCl3
2 Temperature 300.0
3 Experiment 1D
4 Number of Scans 512
5 Spectrometer Frequency 100.62
6 Nucleus 13C
77.16 CDCl3
131.94
124.07
96.58 94.94
41.66 41.43
26.88 26.64 25.83
22.82 22.76
17.74
f1 (ppm)
Intensity

k

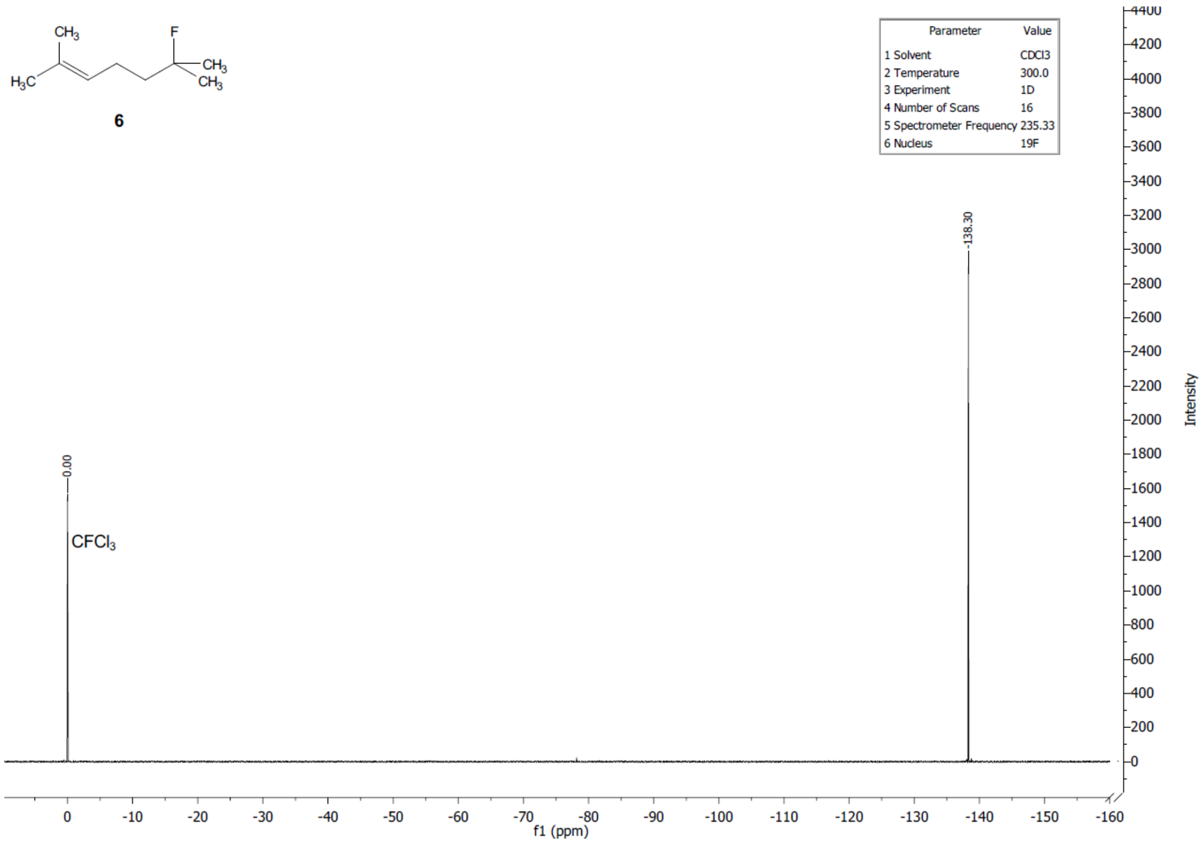
6
Parameter Value
1 Solvent CDCl3
2 Temperature 300.0
3 Experiment 1D
4 Number of Scans 16
5 Spectrometer Frequency 235.33
6 Nucleus 19F
-138.30
0.00
CFCl3
f1 (ppm)
Intensity

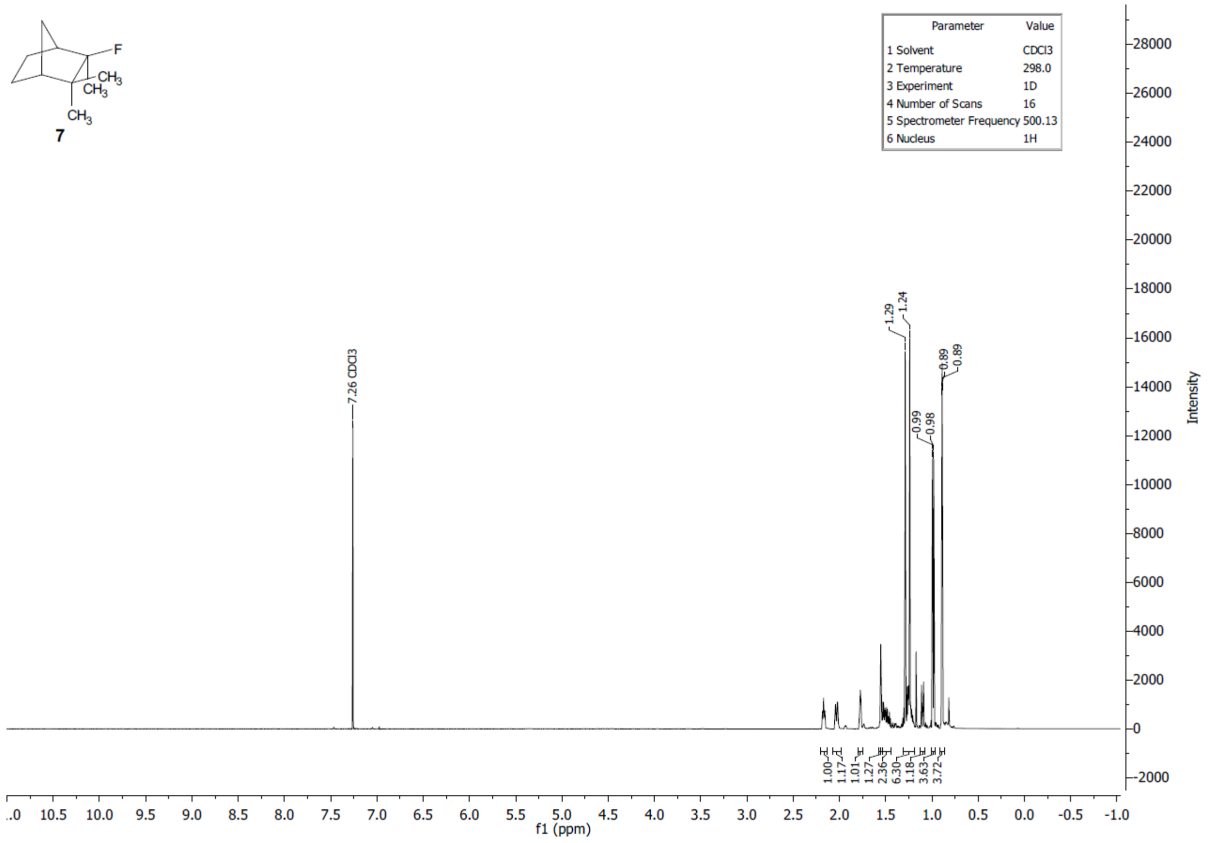
7
Parameter Value
1 Solvent CDCl3
2 Temperature 298.0
3 Experiment 1D
4 Number of Scans 16
5 Spectrometer Frequency 500.13
6 Nucleus 1H
7.26 CDCl3
1.29
1.24
0.99
0.98
0.89
0.89
1.00
1.17
1.01
1.27
2.36
6.30
1.18
3.63
3.72
f1 (ppm)
Intensity

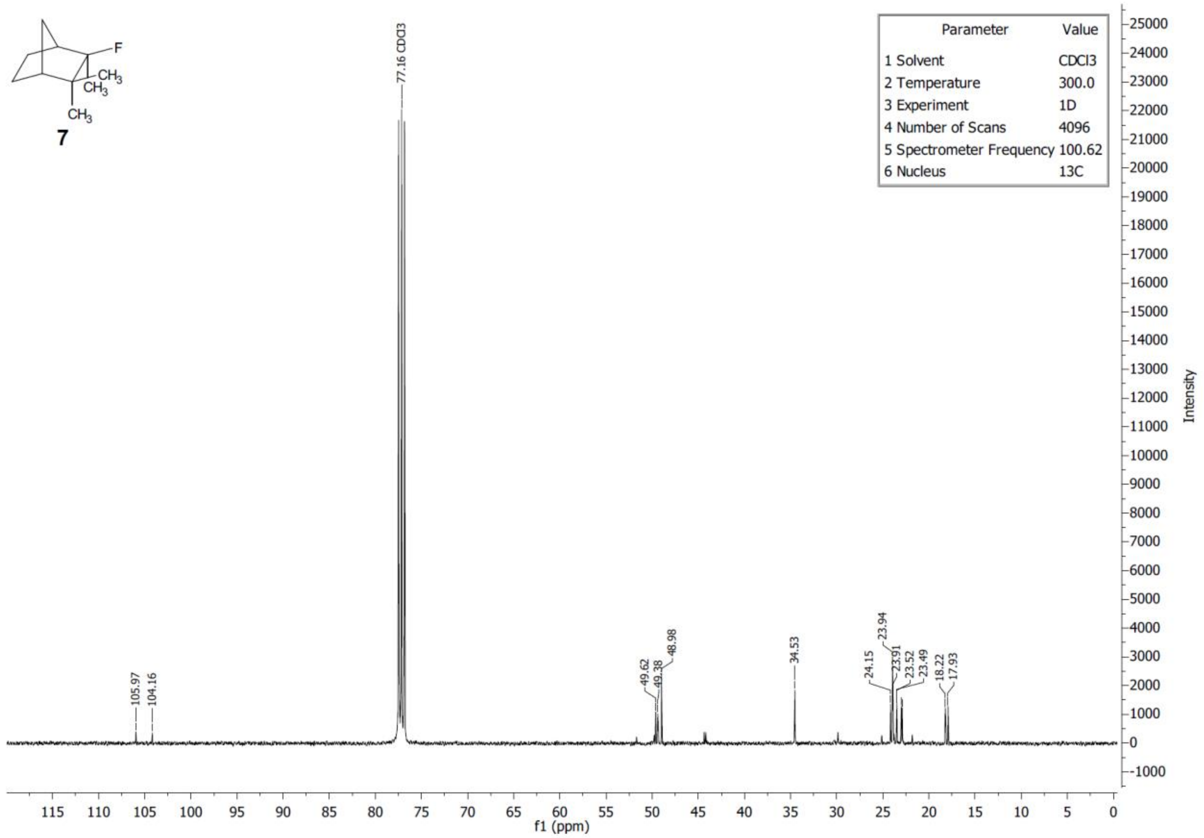
F
CH3
CH3
CH3
7
77.16 CDCl3
Parameter Value
1 Solvent CDCl3
2 Temperature 300.0
3 Experiment 1D
4 Number of Scans 4096
5 Spectrometer Frequency 100.62
6 Nucleus 13C
105.97
104.16
49.62
49.38
48.98
34.53
24.15
23.94
23.91
23.52
23.49
18.22
17.93
f1 (ppm)
Intensity

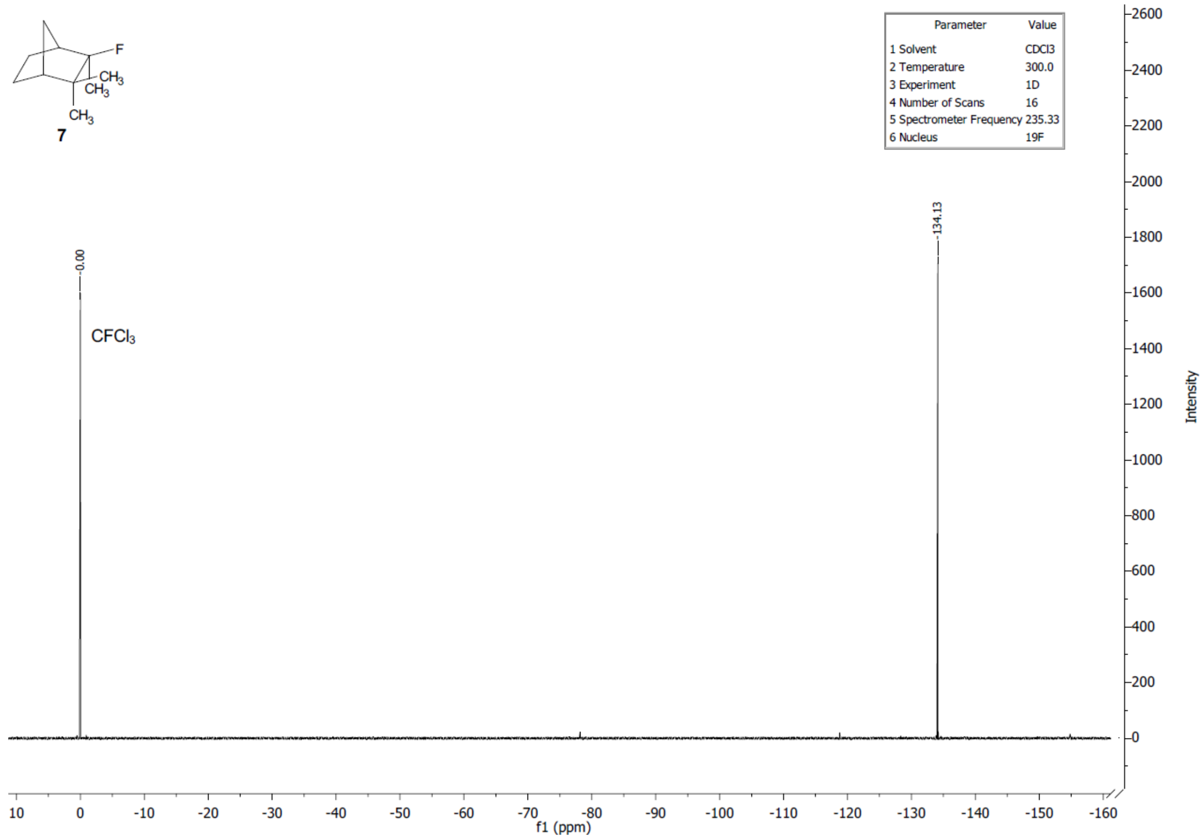
F
CH3
CH3
CH3
7
Parameter Value
1 Solvent CDCl3
2 Temperature 300.0
3 Experiment 1D
4 Number of Scans 16
5 Spectrometer Frequency 235.33
6 Nucleus 19F
-0.00
CFCl3
-134.13
f1 (ppm)
Intensity

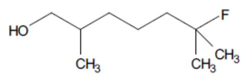

8

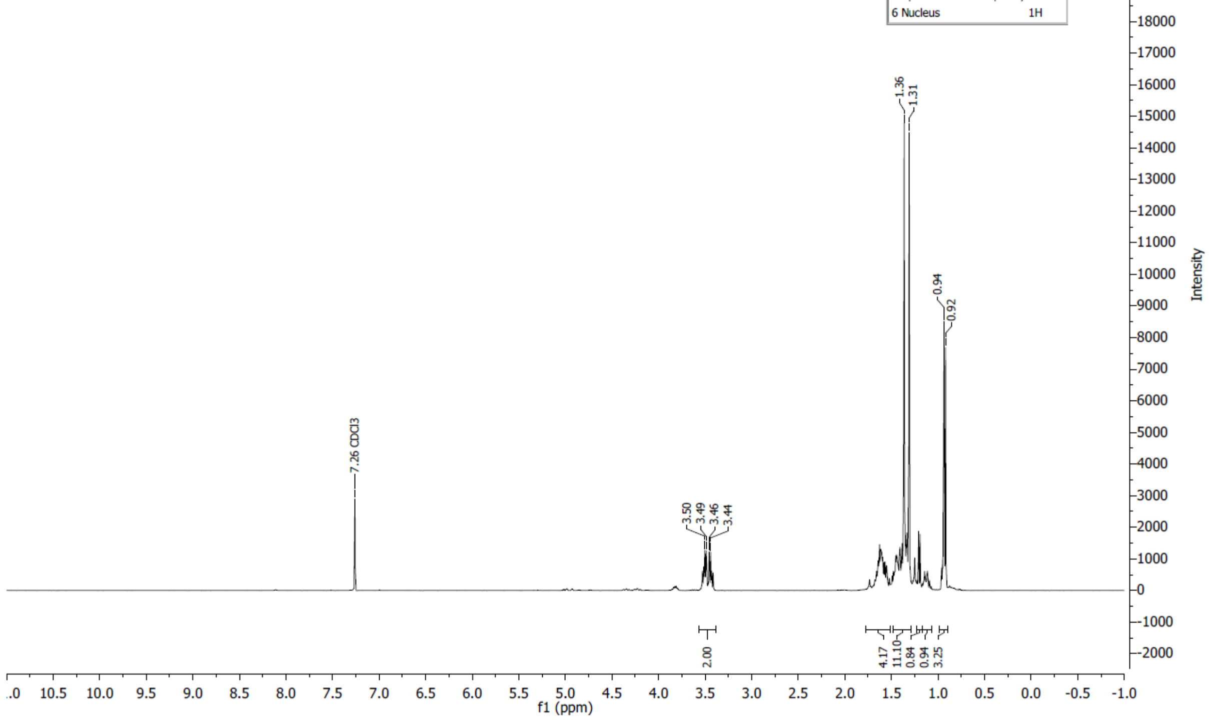

Parameter Value
1 Solvent CDCl3
2 Temperature 300.0
3 Experiment 1D
4 Number of Scans 16
5 Spectrometer Frequency 400.13
6 Nucleus 1H
7.26 CDCl3
3.50 3.49 3.46 3.44
1.36 1.31
0.94 0.92
2.00
4.17 11.10 0.84 0.94 3.25
f1 (ppm)
Intensity

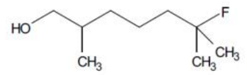

8

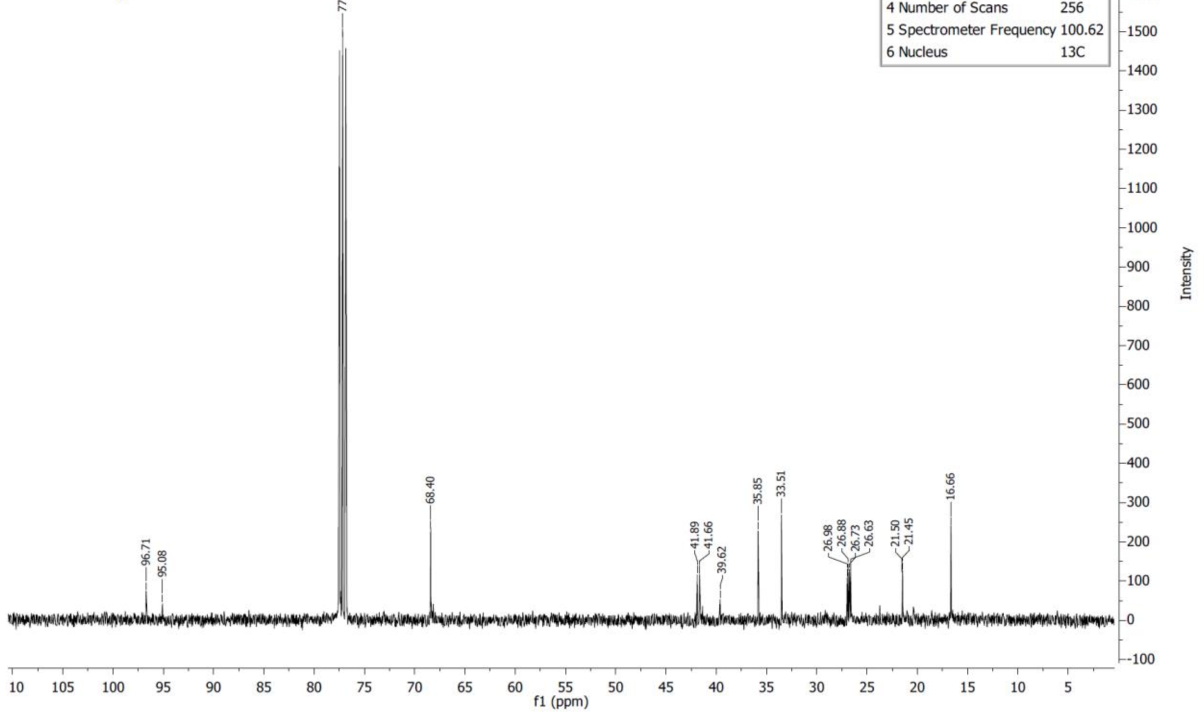

Parameter Value
1 Solvent CDCl3
2 Temperature 300.0
3 Experiment 1D
4 Number of Scans 256
5 Spectrometer Frequency 100.62
6 Nucleus 13C
77.16 CDCl3
96.71 95.08
68.40
41.89 41.66 39.62
35.85 33.51
26.98 26.88 26.73 26.63
21.50 21.45
16.66
f1 (ppm)
Intensity

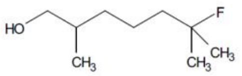
HO
F
CH3
CH3
CH3

8

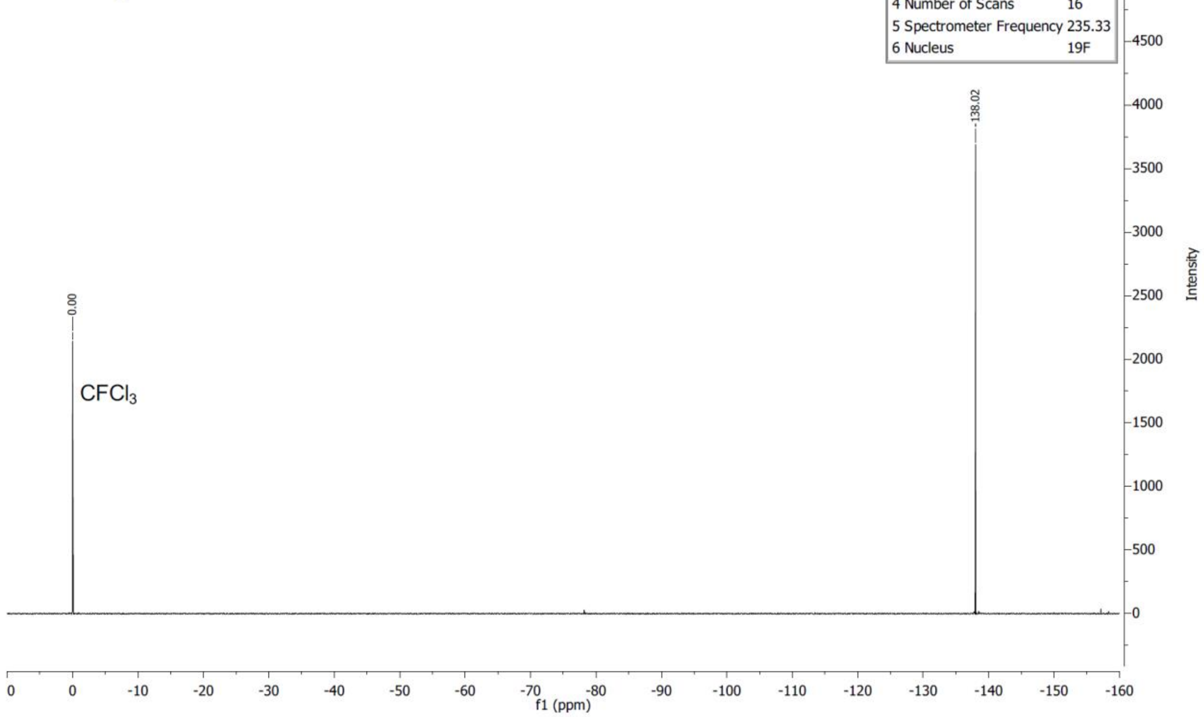
Parameter Value
1 Solvent CDCl3
2 Temperature 300.0
3 Experiment 1D
4 Number of Scans 16
5 Spectrometer Frequency 235.33
6 Nucleus 19F
0.00
CFCl3
-138.02
Intensity
f1 (ppm)

q

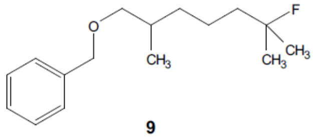
O
F
CH3
CH3
CH3

9

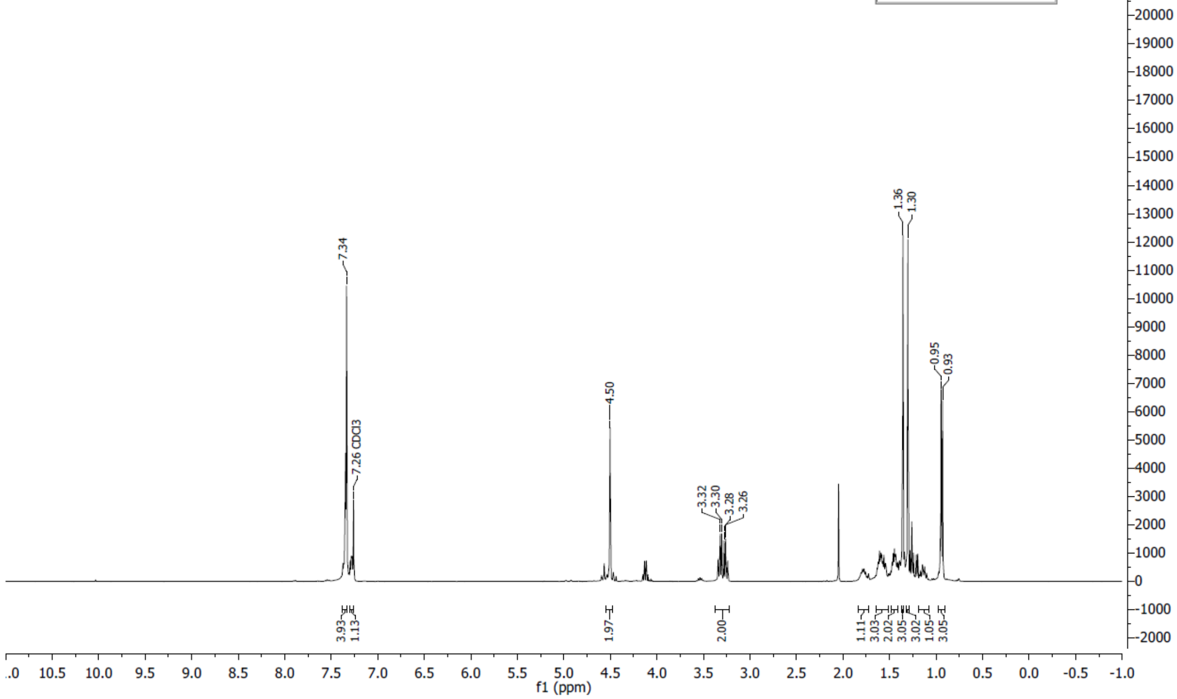
Parameter Value
1 Solvent CDCl3
2 Temperature 300.0
3 Experiment 1D
4 Number of Scans 16
5 Spectrometer Frequency 400.13
6 Nucleus 1H
7.34
7.26 CDCl3
4.50
3.32
3.30
3.28
3.26
1.36
1.30
0.95
0.93
3.93
1.13
1.97
2.00
1.11
3.03
2.02
3.05
3.02
1.05
3.05
f1 (ppm)

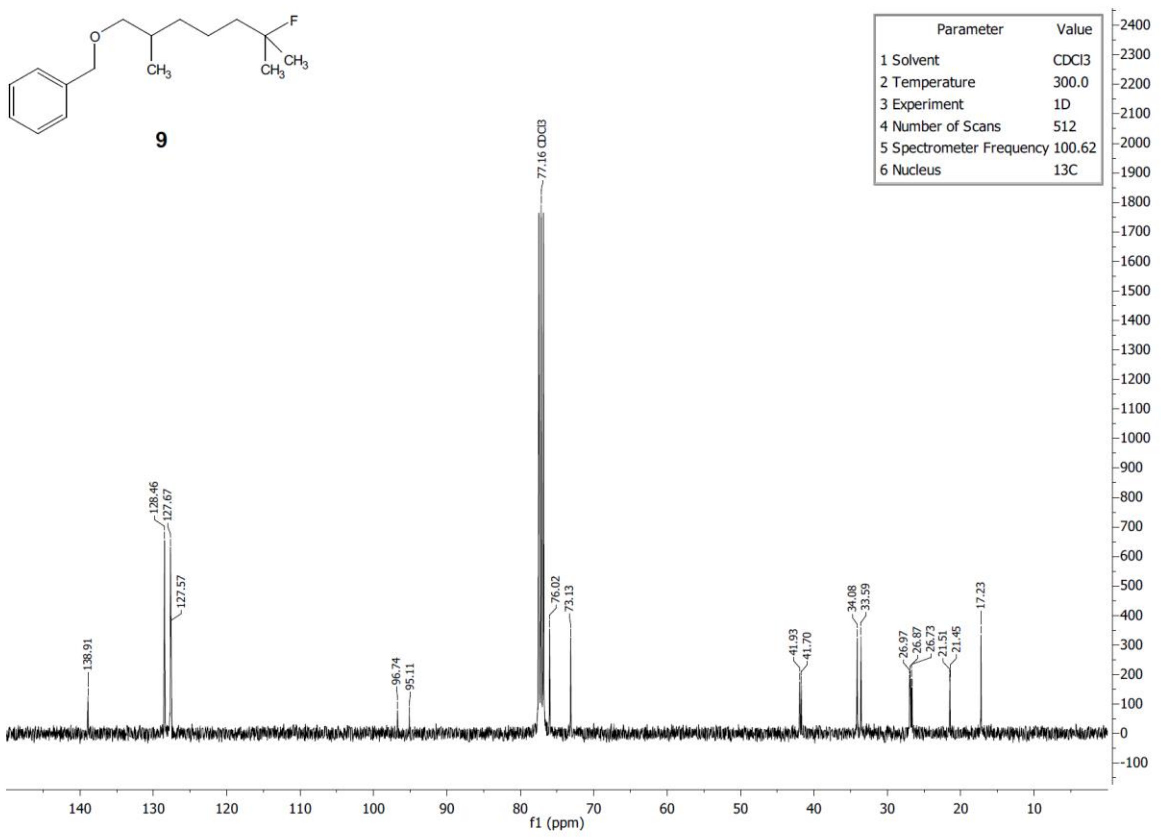

9
Parameter Value
1 Solvent CDCl3
2 Temperature 300.0
3 Experiment 1D
4 Number of Scans 512
5 Spectrometer Frequency 100.62
6 Nucleus 13C
77.16 CDCl3
128.46
127.67
127.57
138.91
96.74
95.11
76.02
73.13
41.93
41.70
34.08
33.59
26.97
26.87
26.73
21.51
21.45
17.23
f1 (ppm)
Intensity

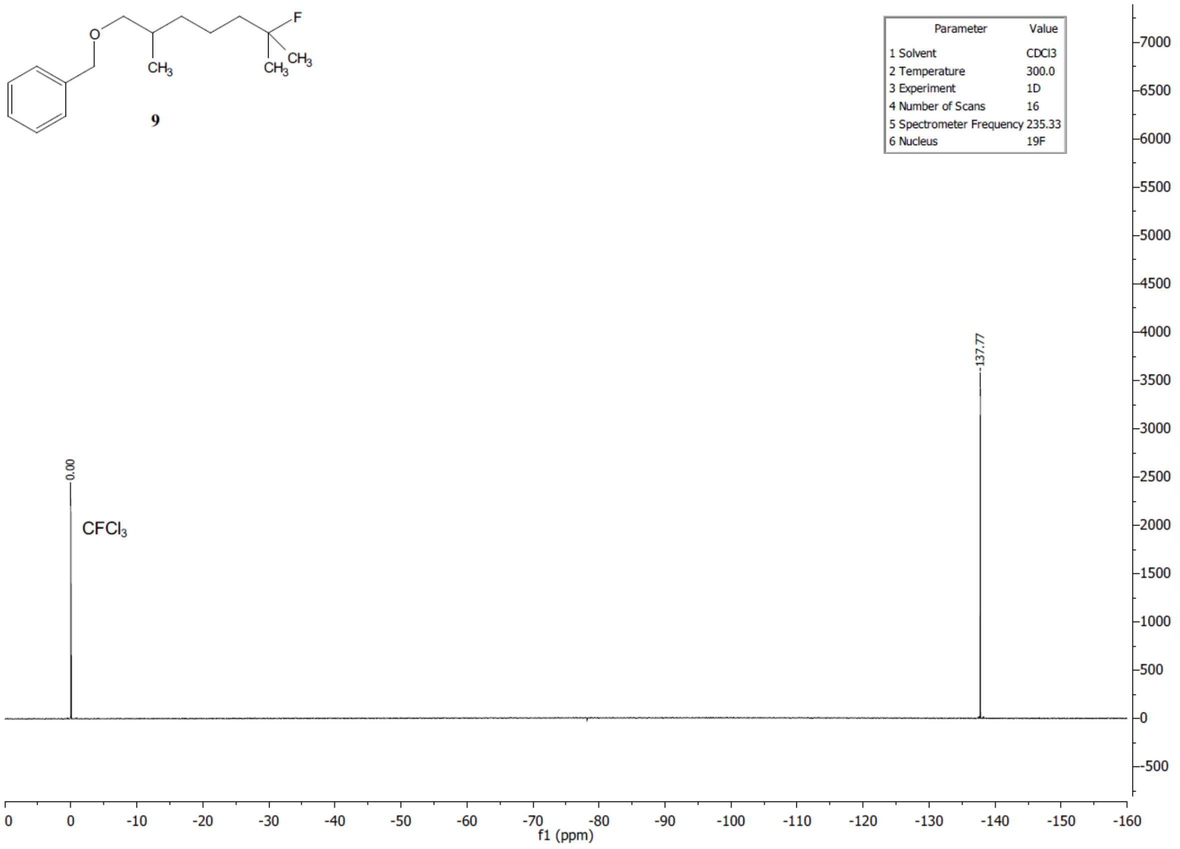

9
Parameter Value
1 Solvent CDCl3
2 Temperature 300.0
3 Experiment 1D
4 Number of Scans 16
5 Spectrometer Frequency 235.33
6 Nucleus 19F
0.00
CFCl3
-137.77
f1 (ppm)
Intensity

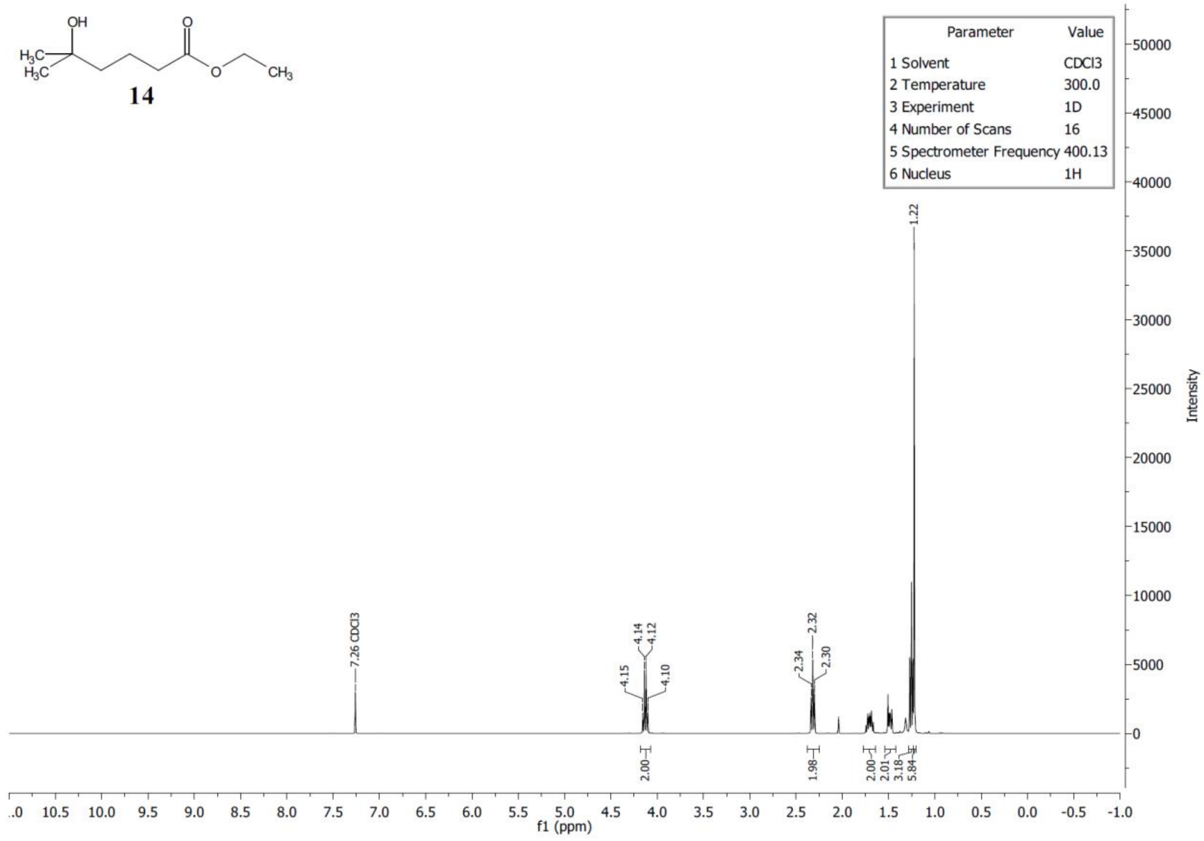
14
Parameter Value
1 Solvent CDCl3
2 Temperature 300.0
3 Experiment 1D
4 Number of Scans 16
5 Spectrometer Frequency 400.13
6 Nucleus 1H
7.26 CDCl3
4.15
4.14
4.12
4.10
2.34
2.32
2.30
1.22
2.00
1.98
2.00
2.01
3.18
5.84
f1 (ppm)
Intensity

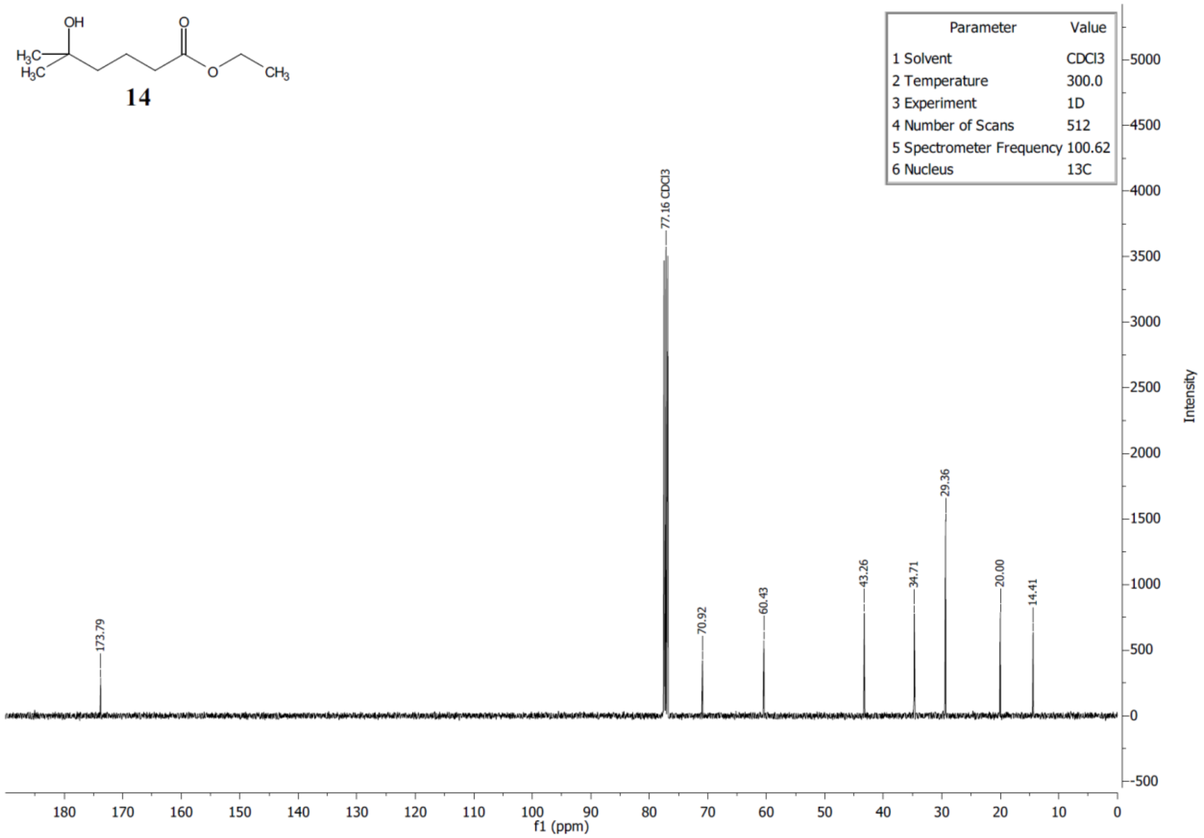
14
Parameter Value
1 Solvent CDCl3
2 Temperature 300.0
3 Experiment 1D
4 Number of Scans 512
5 Spectrometer Frequency 100.62
6 Nucleus 13C
173.79
77.16 CDCl3
70.92
60.43
43.26
34.71
29.36
20.00
14.41
f1 (ppm)
Intensity

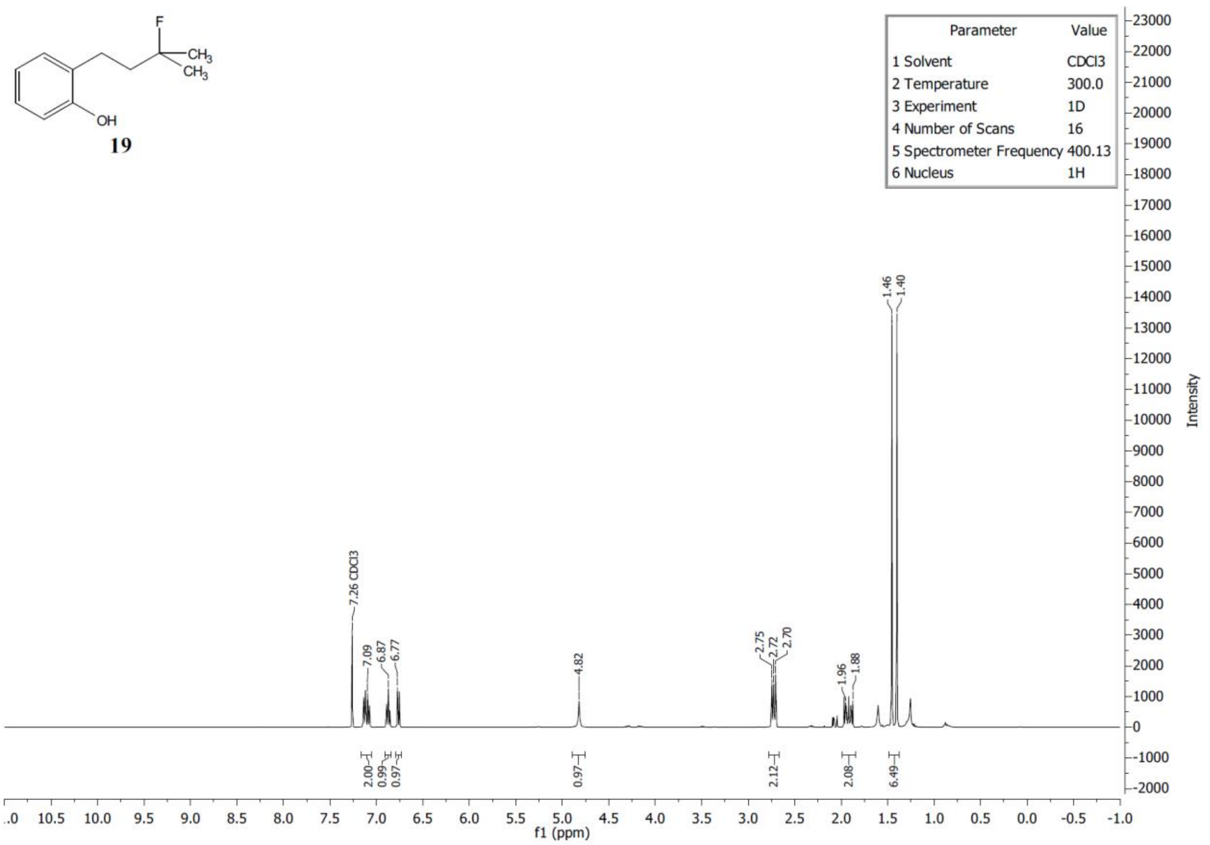
F
CH3
CH3
OH
19
Parameter Value
1 Solvent CDCl3
2 Temperature 300.0
3 Experiment 1D
4 Number of Scans 16
5 Spectrometer Frequency 400.13
6 Nucleus 1H
7.26 CDCl3
7.09
6.87
6.77
4.82
2.75
2.72
2.70
1.96
1.88
1.46
1.40
2.00
0.99
0.97
0.97
2.12
2.08
6.49
f1 (ppm)
Intensity

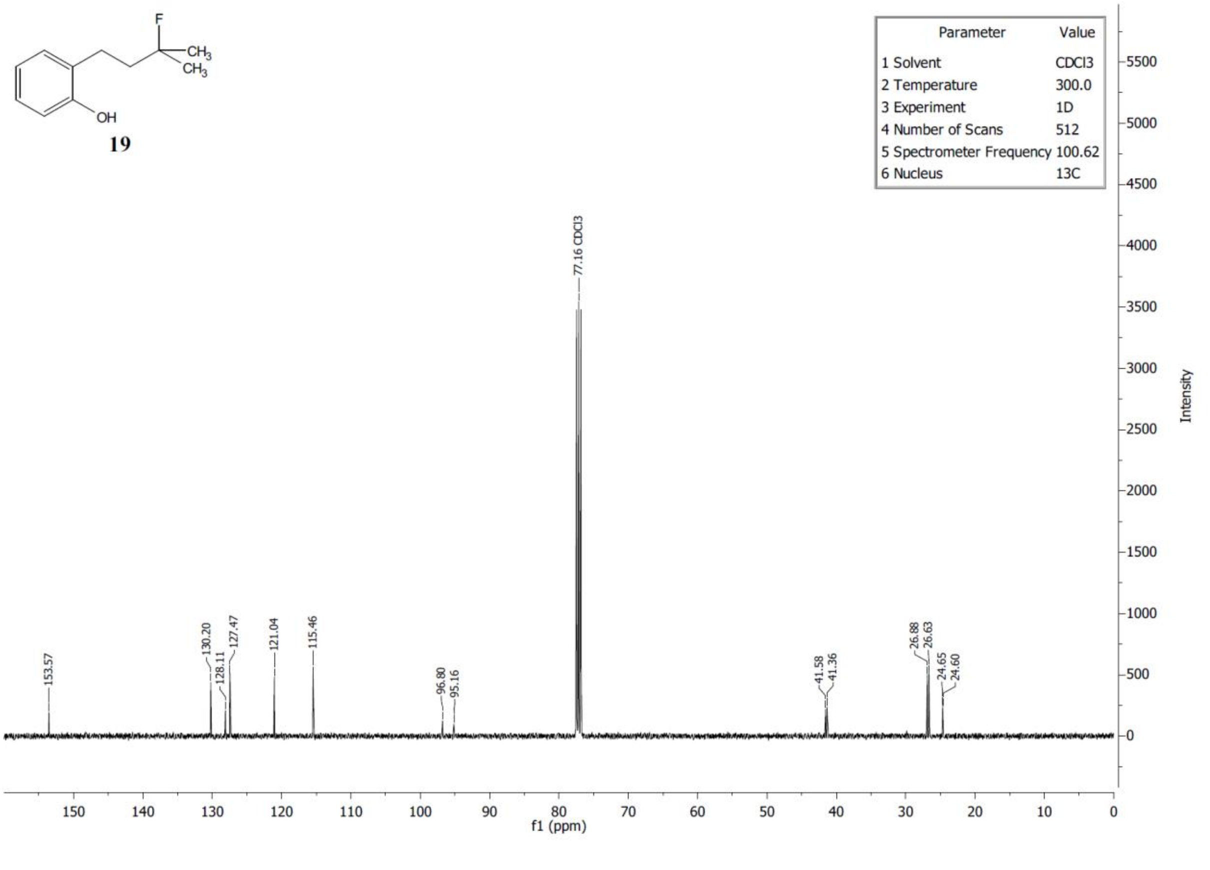
F
CH3
CH3
OH
19
Parameter Value
1 Solvent CDCl3
2 Temperature 300.0
3 Experiment 1D
4 Number of Scans 512
5 Spectrometer Frequency 100.62
6 Nucleus 13C
77.16 CDCl3
153.57
130.20
128.11
127.47
121.04
115.46
96.80
95.16
41.58
41.36
26.88
26.63
24.65
24.60
f1 (ppm)
Intensity

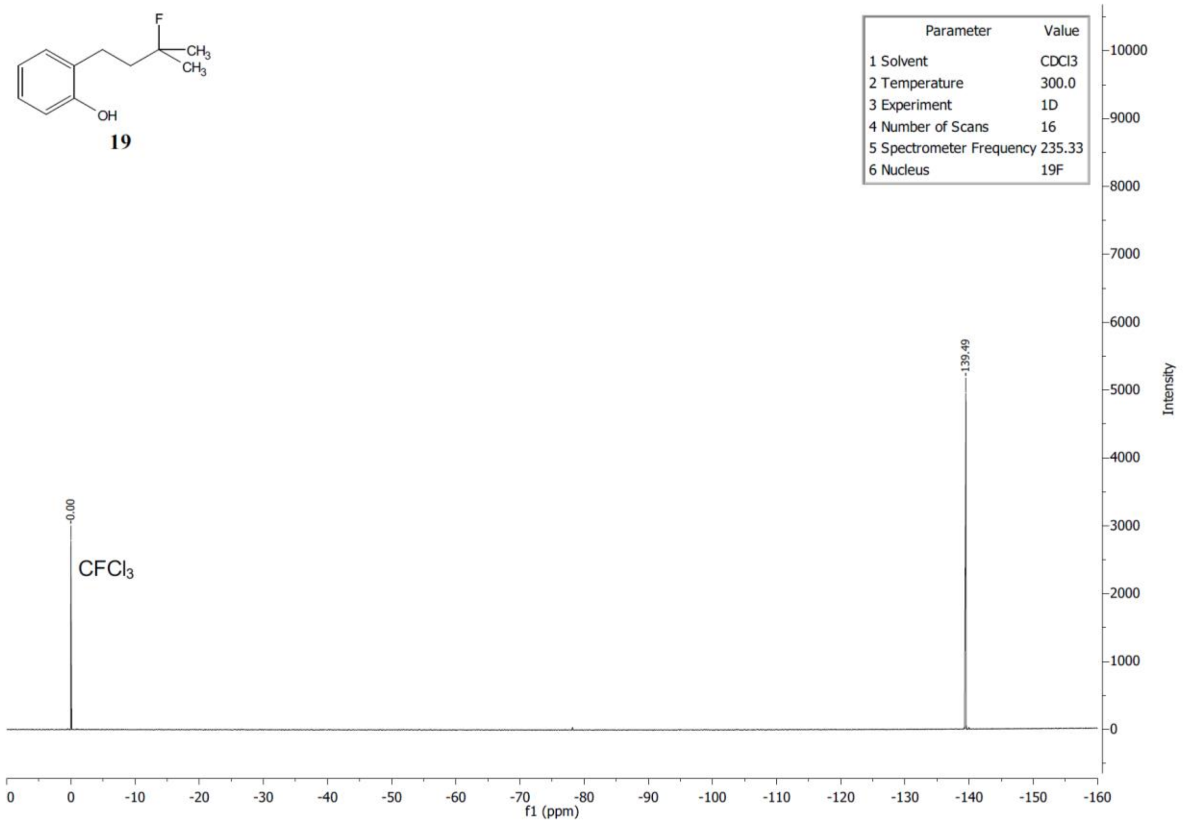

OH
Parameter Value
1 Solvent CDCl3
2 Temperature 300.0
3 Experiment 1D
4 Number of Scans 16
5 Spectrometer Frequency 235.33
6 Nucleus 19F
-0.00
CFCl3
-139.49
Intensity
f1 (ppm)

19

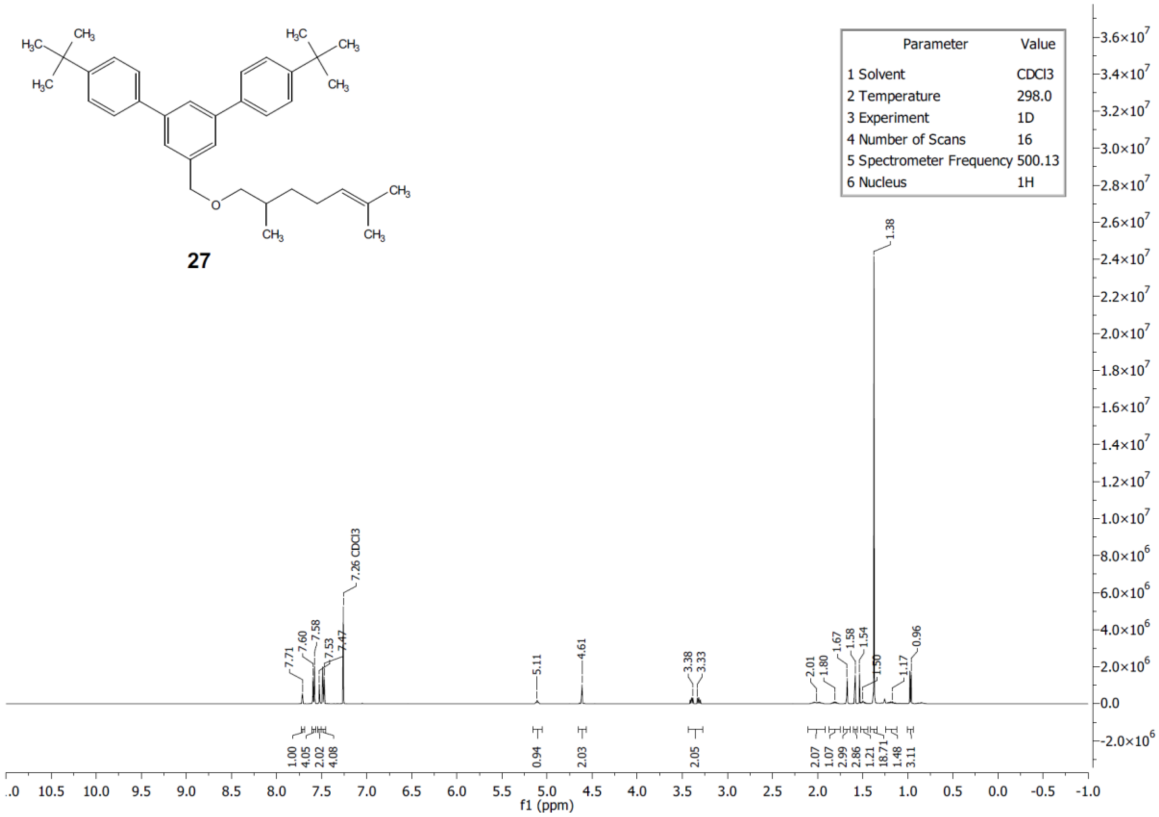

Parameter Value
1 Solvent CDCl3
2 Temperature 298.0
3 Experiment 1D
4 Number of Scans 16
5 Spectrometer Frequency 500.13
6 Nucleus 1H
7.26 CDCl3
7.71 7.60 7.58 7.53 7.47
5.11
4.61
3.38 3.33
2.01 1.80 1.67 1.58 1.54 1.50 1.38 1.17 0.96
1.00 4.05 2.02 4.08
0.94
2.03
2.05
2.07 1.07 2.99 2.86 1.21 18.71 1.48 3.11
f1 (ppm)

27

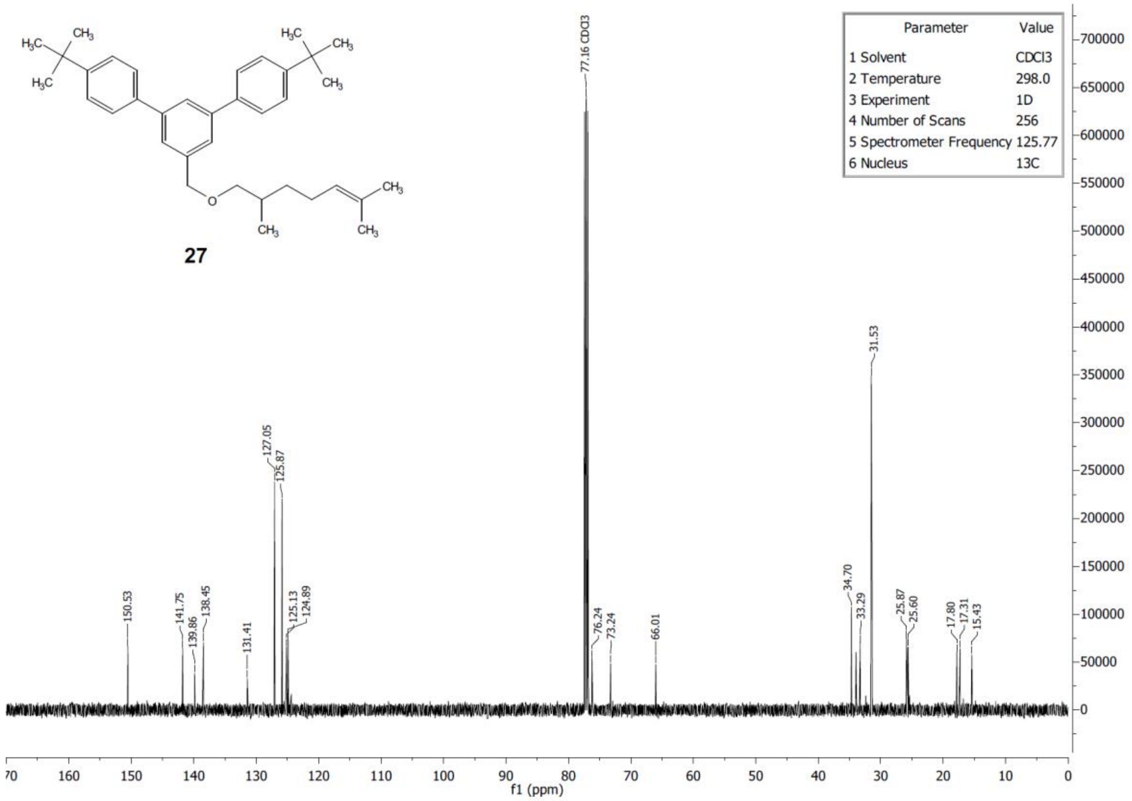
27
Parameter Value
1 Solvent CDCl3
2 Temperature 298.0
3 Experiment 1D
4 Number of Scans 256
5 Spectrometer Frequency 125.77
6 Nucleus 13C
77.16 CDCl3
150.53
141.75
139.86
138.45
131.41
127.05
125.87
125.13
124.89
76.24
73.24
66.01
34.70
33.29
31.53
25.87
25.60
17.80
17.31
15.43
f1 (ppm)

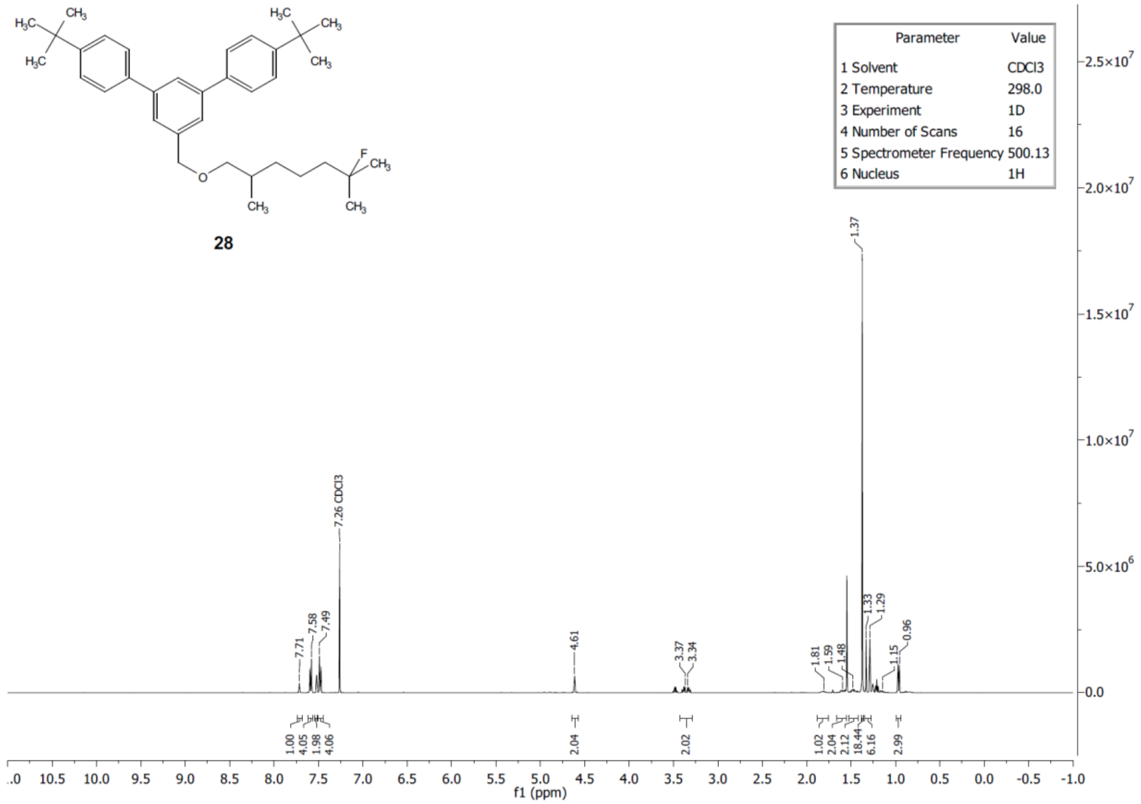
28
Parameter Value
1 Solvent CDCl3
2 Temperature 298.0
3 Experiment 1D
4 Number of Scans 16
5 Spectrometer Frequency 500.13
6 Nucleus 1H
7.71
7.58
7.49
7.26 CDCl3
4.61
3.37
3.34
1.81
1.59
1.48
1.37
1.33
1.29
1.15
0.96
1.00
4.05
1.98
4.06
2.04
2.02
1.02
2.04
2.12
18.44
6.16
2.99
f1 (ppm)

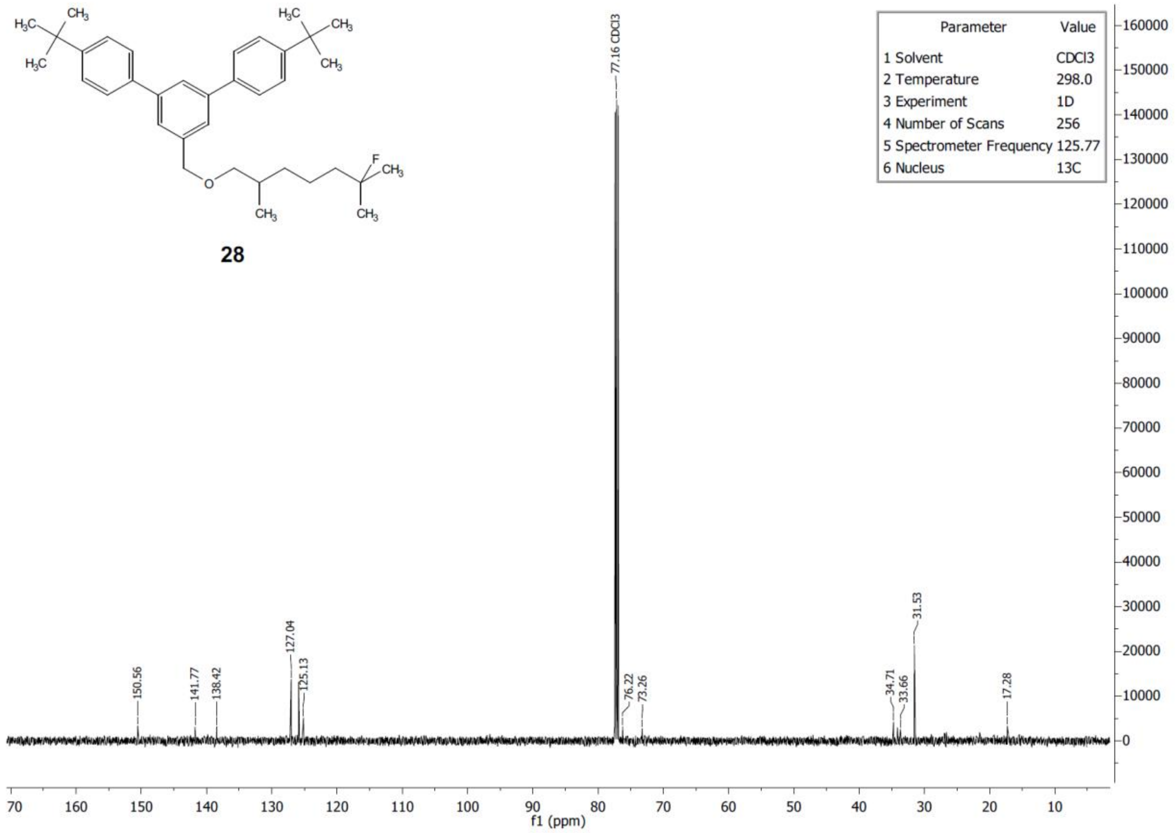
28
Parameter Value
1 Solvent CDCl3
2 Temperature 298.0
3 Experiment 1D
4 Number of Scans 256
5 Spectrometer Frequency 125.77
6 Nucleus 13C
77.16 CDCl3
150.56
141.77
138.42
127.04
125.13
76.22
73.26
34.71
33.66
31.53
17.28
f1 (ppm)

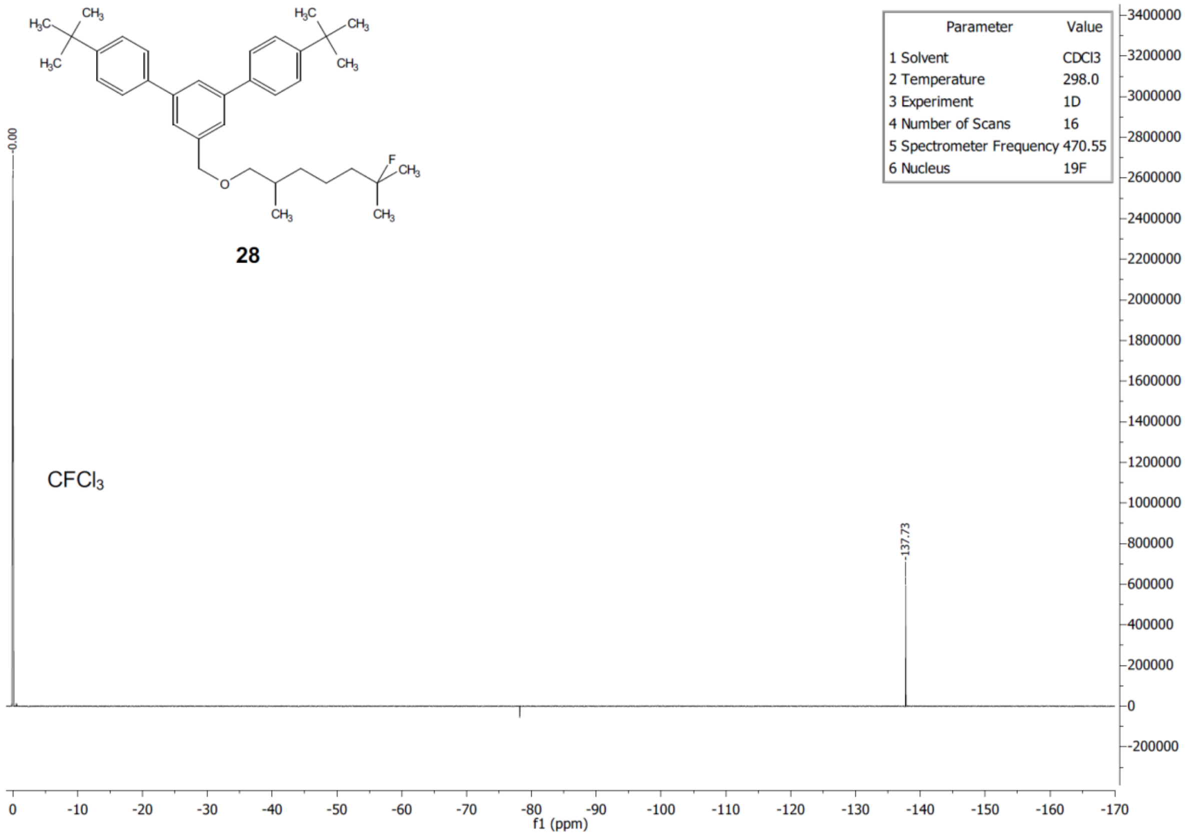
28
Parameter Value
1 Solvent CDCl3
2 Temperature 298.0
3 Experiment 1D
4 Number of Scans 16
5 Spectrometer Frequency 470.55
6 Nucleus 19F
-0.00
CFCl3
-137.73
f1 (ppm)
